# Cannabinoids modulate the microbiota–gut–brain axis in HIV/SIV infection by reducing neuroinflammation and dysbiosis while concurrently elevating endocannabinoid and indole-3-propionate levels

**DOI:** 10.1186/s12974-023-02729-6

**Published:** 2023-03-08

**Authors:** Marina McDew-White, Eunhee Lee, Lakmini S. Premadasa, Xavier Alvarez, Chioma M. Okeoma, Mahesh Mohan

**Affiliations:** 1 Southwest National Primate Research Center, Texas Biomedical Research Institute, 8715 West Military Drive, San Antonio, TX 78227-5302 USA; 2 Department of Pathology, Microbiology, and Immunology, New York Medical College, Valhalla, NY 10595-1524 USA

**Keywords:** THC, SIV, Rhesus macaque, Neuroinflammation, Type-I interferon, Microbiota–gut–brain axis, Endocannabinoids, Indole-3-propionate, Microbiome, *Clostridia*

## Abstract

**Background:**

Although the advent of combination anti-retroviral therapy (cART) has transformed HIV into a manageable chronic disease, an estimated 30–50% of people living with HIV (PLWH) exhibit cognitive and motor deficits collectively known as HIV-associated neurocognitive disorders (HAND). A key driver of HAND neuropathology is chronic neuroinflammation, where proinflammatory mediators produced by activated microglia and macrophages are thought to inflict neuronal injury and loss. Moreover, the dysregulation of the microbiota–gut–brain axis (MGBA) in PLWH, consequent to gastrointestinal dysfunction and dysbiosis, can lead to neuroinflammation and persistent cognitive impairment, which underscores the need for new interventions.

**Methods:**

We performed RNA-seq and microRNA profiling in basal ganglia (BG), metabolomics (plasma) and shotgun metagenomic sequencing (colon contents) in uninfected and SIV-infected rhesus macaques (RMs) administered vehicle (VEH/SIV) or delta-9-tetrahydrocannabinol (THC) (THC/SIV).

**Results:**

Long-term, low-dose THC reduced neuroinflammation and dysbiosis and significantly increased plasma endocannabinoid, endocannabinoid-like, glycerophospholipid and indole-3-propionate levels in chronically SIV-infected RMs. Chronic THC potently blocked the upregulation of genes associated with type-I interferon responses (*NLRC5*, *CCL2*, *CXCL10*, *IRF1*, *IRF7*, *STAT2*, *BST2*), excitotoxicity (*SLC7A11*), and enhanced protein expression of WFS1 (endoplasmic reticulum stress) and CRYM (oxidative stress) in BG. Additionally, THC successfully countered miR-142-3p-mediated suppression of WFS1 protein expression via a cannabinoid receptor-1-mediated mechanism in HCN2 neuronal cells. Most importantly, THC significantly increased the relative abundance of *Firmicutes* and *Clostridia* including indole-3-propionate (*C. botulinum*, *C. paraputrificum*, and *C. cadaveris*) and butyrate (*C. butyricum*, *Faecalibacterium prausnitzii* and *Butyricicoccus pullicaecorum*) producers in colonic contents.

**Conclusion:**

This study demonstrates the potential of long-term, low-dose THC to positively modulate the MGBA by reducing neuroinflammation, enhancing endocannabinoid levels and promoting the growth of gut bacterial species that produce neuroprotective metabolites, like indole-3-propionate. The findings from this study may benefit not only PLWH on cART, but also those with no access to cART and more importantly, those who fail to suppress the virus under cART.

**Supplementary Information:**

The online version contains supplementary material available at 10.1186/s12974-023-02729-6.

## Background

Despite viral suppression by combination anti-retroviral therapy (cART), people living with HIV (PLWH) experience numerous chronic immune activation-driven comorbidities, such as gastrointestinal (GI) dysfunction, cardiovascular and kidney disease, HIV-associated neurocognitive disorders (HAND), etc. [1]. HAND, in particular, is a collective terminology that represents a spectrum of neurocognitive deficits reported in about 40–50% of PLWH. Although the factors triggering HAND are not completely established [2], chronic ongoing neuroinflammation/neuroimmune activation in the face of suppressed viremia is considered to be a significant contributor [3, 4]. The failure of anti-retroviral therapy to fully restore gastrointestinal tract (GIT) function leads to persistence of dysbiosis and epithelial barrier permeability defects, which can facilitate intestinal microbial and by-product translocation into the systemic circulation and impact the functions of central nervous system (CNS) resident cells, such as microglia [5]. Specifically, translocating microbial products may directly contribute to HAND through chronic activation of brain microglia that release increased amounts of proinflammatory cytokines and chemokines leading to neuronal injury and cell death. Hence, there remains an urgent need for therapeutic support to PLWH with HAND symptoms for whom cART alone will not suffice [3, 4].

The GIT and the brain are major targets of HIV/SIV and significantly impacted very early in infection [6, 7]. Infection of the GIT results in severe CD4^+^ T cell depletion, immune dysfunction, dysbiosis, and significant structural and functional damage [6, 8, 9]. Similarly, HIV/SIV enters the CNS within 2 weeks post-infection, primarily via infected CD4^+^ T cells [10] and monocytes/macrophages that transport the virus across the blood brain barrier (BBB) and facilitate infection of CNS resident cells, perivascular macrophages, and microglia [7, 11]. Simultaneous infection of both organs can impair signaling from the gut to the brain and vice versa, thereby leading to dysregulation of the gut–brain axis, a bidirectional communication network between the GIT and the brain.

During the past decade, the microbiota has emerged as a key player in the bidirectional communication between the gut and the brain and its ability to modulate this crosstalk has garnered increased scientific interest, establishing the microbiota–gut–brain axis (MGBA) as a major research field [12–14]. Signaling in the MGBA can occur via various routes including the immune system, neurochemical signaling, tryptophan metabolism, enteric nervous system pathways and the vagus nerve, and via the production of bacterial metabolites such as bioactive peptides, short chain fatty acids (SCFAs), branched-chain amino acids, peptidoglycans, etc. [12–14]. Although importance of the MGBA in HIV infection has been highlighted [15, 16], detailed studies to investigate the impact of HIV on the MGBA are lacking and needed immediately.

Despite significant research efforts invested in understanding HAND pathogenesis, these findings are yet to translate into therapeutic results. Accordingly, the identification and development of feasible, safe, and inexpensive disease-modifying strategies to dampen residual CNS inflammation and improve the overall quality of life of PLWH are desperately needed. Cannabinoid based drugs such as dronabinol (Marinol® and Syndros®), an orally administered cannabinoid agonist [synthetic delta-9-tetrahydrocannabinol; (THC)] is FDA approved to increase appetite, weight gain, and potentially reduce the gastrointestinal adverse effects of anti-retroviral drugs [17]. Recently, Watson et al. [18] reported a reduced likelihood of neurocognitive impairment in PLWH, who had prior exposure to cannabis/cannabinoids. Nevertheless, the molecular mechanisms underlying potential neuroprotective effects of cannabinoids and its impact on gut microbiome signaling, remains unknown and unaddressed. Such studies are challenging to perform in humans because of the very limited or unavailability of post-mortem brain tissues for molecular studies. Similarly, since it is impossible to collect colon contents, almost all human microbiome profiling studies have and continue to focus on the fecal microbiota. It is important to note that the colon contains several 100-fold more microbes than any other intestinal segment and is a major source of microbial metabolites that can impact host physiology. Therefore, examining the colon microbiota instead of fecal microbiota is of significant interest to human health [19]. The availability of the biological relevant SIV-infected rhesus macaque (RM) model of HIV infection offsets these limitations as it enables the performance of controlled THC studies and collection of distinct brain regions (basal ganglia) and colonic contents at necropsy for in-depth molecular studies.

Accordingly, controlled studies in SIV-infected RMs showed that long-term, low-dose THC treatment that is analogous to orally administered Marinol® or Syndros®, slowed disease progression, prolonged survival, and attenuated infection-induced inflammation [20, 21]. Consistent with the role of the GIT as the central organ of endocannabinoid signaling [22], we previously demonstrated the ability of chronic low-dose THC administration to inhibit proinflammatory miRNA and gene expression [23], and the percentage of Ki67 and programmed death 1 expressing CD4^+^ and CD8^+^ T cells during acute and chronic SIV infection of RMs [24]. Interestingly, HIV-infected individuals who consumed cannabis products were found to have relatively reduced plasma HIV-1 viral load [25], circulating CD16^+^ monocytes and plasma IP-10 levels [26], frequencies of activated T cells and monocytes [27], and inflammatory markers in cerebrospinal fluid (CSF) and blood, thus confirming the findings in SIV-infected RMs [28]. More recently, we demonstrated the ability of cannabinoids to directly target the indoleamine 2–3 dioxygenase pathway via a cannabinoid receptor-2 (CB2R) mechanism in chronically SIV-infected RMs [29]. This resulted in reduced plasma concentrations of kynurenine and quinolinate, two important tryptophan metabolites that have been proposed to perturb brain functions and cause depression like symptoms. Since 90% of tryptophan is metabolized via the kynurenine pathway, we hypothesized that inhibition of the indoleamine 2–3 dioxygenase-1 (*IDO1*) may increase tryptophan availability for conversion to neuroprotective indole derivatives that are crucial regulatory factors important for the gut–brain axis. Moreover, while modulating the MGBA using probiotics has received considerable attention in neurodegenerative diseases, such studies are in its infancy in HIV infection [30].

Here, we investigated the impact of HIV/SIV infection on the MGBA as a possible key determinant of neuroinflammation/neuroimmune activation and whether MGBA dysregulation can be reversed using low-dose cannabinoids. We demonstrated that long-term, low-dose THC administration to chronically SIV-infected RMs significantly attenuated expression of genes associated with type-I interferon (IFN) response, excitotoxicity (*SLC7A11*), endoplasmic (*WFS1*) and oxidative stress (*CRYM*-pipecolate pathway) in basal ganglia (BG). Further, we show that THC can override the suppressive effects of miR-142-3p, a key neuroinflammatory miRNA markedly upregulated in BG of vehicle-treated SIV (VEH/SIV) but not in THC-treated SIV (THC/SIV) RMs, on WFS1 protein expression via a cannabinoid receptor 1 (CB1R)-mediated mechanism. Furthermore, exogenous THC significantly increased plasma endocannabinoid, glycerophospholipid, and the gut bacteria-derived entero-/neuro-/cardioprotective indole-3-propionate (IPA) levels. Finally, chronic THC positively modulated the colonic microbiome by significantly increasing the relative abundance of *Firmicutes*, *Clostridia*, *Lactobacilli*, *Bifidobacteria*, SCFA producers *Clostridium butyricum*, *Faecalibacterium prausnitzii*, and *Butyricicoccus pullicaecorum* and more importantly, IPA-producing *Clostridium botulinum*, *Clostridium paraputrificum*, and *Clostridium cadaveris*. Overall, as emphasized by Keimpema et al. [31], our findings provide relevant novel and mechanistic insights into phytocannabinoid-mediated modulation of the MGBA. These effects potentially involve both cannabinoid receptor-mediated anti-inflammatory/anti-oxidant effects in the brain and gut [24, 29], and receptor independent enhancement of the production of endocannabinoid and beneficial gut bacteria-derived neuroprotective indoles (IPA) that, with additional animal validation studies, may benefit not only PLWH but also those suffering from neurodegenerative diseases like Alzheimer’s disease (AD), Parkinson’s disease (PD), Huntington’s disease (HD), etc.

## Methods

### Animal care, ethics and experimental procedures

All experiments using rhesus macaques were approved by the Tulane Institutional Animal Care and Use Committee (Protocol Nos-3581 and 3781). The Tulane National Primate Research Center (TNPRC) is an Association for Assessment and Accreditation of Laboratory Animal Care International accredited facility (AAALAC #000594). The NIH Office of Laboratory Animal Welfare assurance number for the TNPRC is A3071-01. All clinical procedures, including administration of anesthesia and analgesics, were carried out under the direction of a laboratory animal veterinarian. Animals were anesthetized with ketamine hydrochloride for blood collection procedures. Intestinal pinch biopsies were performed by laboratory animal veterinarians. Animals were pre-anesthetized with ketamine hydrochloride, acepromazine, and glycopyrrolate, intubated and maintained on a mixture of isoflurane and oxygen. All possible measures were taken to minimize the discomfort of all the animals used in this study. Tulane University complies with NIH policy on animal welfare, the Animal Welfare Act, and all other applicable federal, state and local laws.

### Animal model and experimental design

Sixty age- and weight-matched adult Indian RMs were randomly divided into 3 groups. Group-1 (*n* = 11), (seven received twice daily injections of vehicle (VEH) and three did not) and were infected intravenously with 100TCID_50_ of SIVmac251. Group-2 (*n* = 11) received twice daily injections of THC similar to group 1 for 4 weeks prior to SIV infection until 6 months post-SIV infection. Group 3 (*n* = 38) served as controls and remained uninfected. The animals used in the current study were studied in two cohorts (Table 1). The global shortage of Indian rhesus macaques, resulting from the unforeseen demand caused by the COVID-19 pandemic [32] made it harder and extremely challenging to find a large number of uninfected control male RMs. Therefore, we included female RMs that were immediately available at our facility for use in Group 3, which contained 29 male RMs and nine female RMs, while groups 1 and 2 comprised only male RMs.

**Table 1:** Animal and viral load information for vehicle or THC-treated chronic SIV-infected and uninfected rhesus macaques

Animal ID	SIV inoculum	Sex	Duration of infection	Plasma viral loads 10^6^/mL	Brain viral loads 10^6^/mg RNA	Brain histopathology	Colon viral loads 10^6^/mg RNA
Chronic SIV-infected and vehicle-treated (Group 1) (*n* = 11)							
IH96^a,b,c,d,e,f^	SIVmac251	Male	180	0.1	2	ND	786
HV48^a,b,c,d,e,f^	SIVmac251	Male	150	4	1	ND	147
IN24^a,b,c,d,e,f^	SIVmac251	Male	180	9.4	0.4	ND	29
JC81^a,b,c,d,e,f^	SIVmac251	Male	180	0.38	0.3	ND	320
JH47^a,b,c,d^	SIVmac251	Male	180	2	0.07	ND	300
JR36^b,c,d^	SIVmac251	Male	180	0.5	0.2	ND	20
JD66^a,b,c,d^	SIVmac251	Male	180	0.04	0.2	ND	4
IV95^d^	SIVmac251	Male	180	0.02	2.0	ND	2
HB31^e^	SIVmac251	Male	180	3000	NA	SIV syncytia/encephalitis	200
GA19^e^	SIVmac251	Male	180	100	NA	ND	600
FT11^e^	SIVmac251	Male	145	500	NA	ND	2075
Chronic SIV-infected and Δ^9^-THC treated (Group 2) (*n* = 11)							
GV60^a,b,e^	SIVmac251	Male	180	18.9	40,000	SIV syncytia/encephalitis	6726
HT48^e^	SIVmac251	Male	150	260	1750	ND	9360
IA83^a,b,c,e,f^	SIVmac251	Male	180	1.5	20	ND	1261
IH69^a,b,e,f^	SIVmac251	Male	180	0.06	3	ND	93.8
HI09^a,b,c,e,f^	SIVmac251	Male	180	0.01	0.04	ND	3.0
JB82^a,b,c,e,f^	SIVmac251	Male	180	7.7	2	ND	970
IA04^a,b,c,e,f^	SIVmac251	Male	150	0.66	1	ND	35
JI45^c^	SIVmac251	Male	180	3	0.01	ND	10
JT80^c^	SIVmac251	Male	180	1	0.04	ND	300
JC85^c^	SIVmac251	Male	180	0.02	0.09	ND	1
IV90^c^	SIVmac251	Male	180	0.02	0.06	ND	10
Uninfected controls (Group 3) (*n* = 38)							
HF34, HH69, HH75^a,b,d^	NA	Males	NA	NA	NA	NA	NA
GV92, IR97^a,d^	NA	Males	NA	NA	NA	NA	NA
KG97, LH65, MF62^c^	NA	Females	NA	NA	NA	NA	NA
ML83, MM32^c^, JE51^a,b,d^	NA	Females	NA	NA	NA	NA	NA
IT18, KF42, GT18^d^, GT20^e^	NA	Males	NA	NA	NA	NA	NA
KD17^d^, EH70, EH80^e^	NA	Females	NA	NA	NA	NA	NA
EL66, HT73, HT22, HN64^e^	NA	Males	NA	NA	NA	NA	NA
LA88, LC39, LD08, LE67^f^	NA	Males	NA	NA	NA	NA	NA
KV50 LA55, LA89, LB61^f^	NA	Males	NA	NA	NA	NA	NA
LC48, LH75, LM56, LN60^f^	NA	Males	NA	NA	NA	NA	NA
LH92, LI21, LI81, LM85^f^	NA	Males	NA	NA	NA	NA	NA

*NA* not applicable, *ND* none detected

^a^Denotes animals used for RNA-seq (mRNA profiling) studies

^b^Denotes animals used for microRNA profiling studies

^c^Denotes animals used for WFS1 and CRYM immunofluorescence (confocal) studies

^d^Denotes animals used for miR-142-3p RT-qPCR validation studies

^e^Denotes animals used for shotgun metagenomic sequencing (colonic microbiome analysis)

^f^Denotes animals use for metabolomics

Chronic administration of THC or VEH was initiated by the intramuscular route 4 weeks before SIV infection at 0.18 mg/kg as used in previous studies [23, 24]. This dose of THC was found to eliminate responding in a complex operant behavioral task in almost all animals [21]. The dose was subsequently increased for each subject to 0.32 mg/kg, over a period of approximately 2 weeks when responding was no longer affected by 0.18 mg/kg daily (i.e., tolerance developed), and maintained for the duration of the study. The optimization of the THC administration in RMs accounts for the development of tolerance during the initial period of administration. Since this dose of THC showed protection in our previously published studies [23, 24], the same dose was used. The 0.32 mg/kg dose was also shown to be effective in SIV-infected RMs of Chinese origin [33]. SIV levels in plasma and BG were quantified using the TaqMan One-Step Real-time RT-qPCR assay that targeted the LTR gene [24, 29]. At necropsy, entire BG tissue was collected in RNAlater (Thermo Fisher Scientific) and Z-fix for total RNA extraction and embedding in paraffin blocks, respectively.

### RNA-seq library construction, clustering and sequencing

Transcriptome profiling by RNA-seq and data analysis were performed by Novogene (Sacramento, CA) as reported previously [29, 34] and described in Additional file 3: Additional Methods.

### Immunofluorescence for WFS1 and CRYM localization

Immunofluorescence studies for the detection of WFS1 (1 in 200 dilution) (Abcam, Cat No: ab230512) and CRYM (1 in 200 dilution) (Abcam, Cat No: ab220085), CB1R (1 in 50 dilution) (Abcam, Cat No: ab23703) and CB2R (1 in 50 dilution) (Abcam, Cat No: ab3560) was performed as described previously [29, 34, 35]. Neuronal expression of WFS1 and CRYM positive cells was confirmed using NeuN (1 in 100) (Abcam, Cat No: ab104224) and appropriate Alexa Fluor conjugated secondary antibodies (Thermo-Fisher).

### Global microRNA expression profiling

MicroRNA expression profiling was performed using TaqMan OpenArray Human MicroRNA panels (Thermo Fisher Scientific) as reported previously [29, 34, 35] and described in Additional file 3: Additional Methods.

### Quantitative real-time TaqMan and SYBR Green RT-qPCR assay for OpenArray® validation

Expression of miR-142-3p was quantified in BG tissue using the TaqMan micro-RNA predesigned and preoptimized assays (Thermo Scientific) reported previously [29, 35] and described in Additional file 3: Additional Methods.

### Cloning of 3′-UTR of WFS1 mRNA and Dual-Glo luciferase reporter gene assay

The 3′ UTR of the rhesus macaque *WFS1* mRNA contains a single predicted miR-142-3p binding site (TargetScan 7.2) [36]. Accordingly, a short 54 nucleotide sequence representing the 3′ UTR containing the predicted miR-142-3p site (5′-AGGCGGCGCACUGGCAGUGUGUCACACUGAGCACAG**CACUACA**GGCUGCCUCAU-3′) was synthesized (IDTDNA Technologies Inc., IA) for cloning into the pmirGLO Dual-Luciferase vector (Promega Corp, Madison, WI) [24, 29, 34, 37]. A second oligonucleotide with the miRNA binding site deleted (*n* = 7 nucleotides) (5′-AGGCGGCGCACUGGCAGUGUGUCACACUGAGCACAGGGCUGCCUCAU-3′) was also synthesized to serve as a negative control. PmirGLO vector cloning and dual luciferase reported assays were performed as reported previously [24, 29, 34, 37] and described in Additional file 3: Additional Methods.

### miR-142-3p overexpression studies and endocannabinoid control of WFS1 expression in primary HCN2 neuronal cells

To determine the impact of miR-142-3p on *WFS1*, we overexpressed FAM-labeled locked nucleic conjugated miR-142-3p mimics (Qiagen Inc) in primary human HCN2 neuronal cells (ATCC, USA) as WFS1 protein expression was strong and exclusively expressed in BG neurons. HCN2 cells were cultured in CnT Prime medium (CnT-PR, CELLnTEC Advanced Cell Systems AG, Bern, Switzerland) in 8 well chamber slides (Thermo-Fisher Scientific Waltham, MA USA) at 37 °C in a humidified atmosphere with 5% CO_2_. At 50–60% confluency, cells were transfected with 30 nM of FAM-LNA- miR-142-3p or FAM-LNA-negative control mimic using the Lipojet transfection reagent (Signagen, DE). Cells were fixed with 2% paraformaldehyde at 96 h post-transfection and immunostained with WFS1 and later with DAPI for nuclear localization.

To determine the impact of THC and the endocannabinoid mechanisms regulating *WFS1* expression, miR-142-3p transfected cells (cultured as described in the previous section) were either treated with 3 µM THC or preincubated with the 10 µM cannabinoid receptor 1 inverse agonist AM251 (Tocris Bioscience, Minneapolis, MN) or the cannabinoid receptor 2 antagonist AM630 (Tocris Bioscience, Minneapolis, MN) for 1 h followed by 3 µM THC treatment and incubation for 18 h. At the end of the incubation period, cells were fixed with 2% paraformaldehyde in PBS for WFS1 immunofluorescence staining.

### Quantification of endocannabinoid and tryptophan metabolites in plasma

Sample preparation was performed by Metabolon Inc (Morrisville NC), as reported previously [29], and in Additional file 3: Additional Methods.

### Shotgun metagenomic sequencing of colon microbiota

Total DNA was isolated from colon contents of VEH/SIV, THC/SIV, and uninfected control RMs using the cell-free DNA purification kit (Norgen Biotek Corp, ON, Canada) following the manufacturer’s protocol. Shotgun metagenomic sequencing and data analysis was performed by LC Sciences, Houston, Texas, as reported previously [29].

### Quantitation of mucosal viral loads

Total RNA samples from all SIV-infected animals were subjected to a quantitative real-time TaqMan One-step RT-qPCR analysis to determine the viral load in plasma and BG tissue. Briefly, primers and probes specific to the SIV LTR sequence were designed and used in the real-time TaqMan PCR assay. Probes were conjugated with a fluorescent reporter dye (FAM) at the 5′ end and a quencher dye at the 3′ end. Fluorescence signal was detected with an ABI Prism 7900 HT sequence detector (Thermo Fisher). Data were captured and analyzed with Sequence Detector Software (Thermo Fisher). Viral copy number was determined by plotting *C*_T_ values obtained from the plasma and BG tissue samples against a standard curve (*y* = − 3.33*x* + 40.3) (*r*^2^ = 0.999) generated with in vitro transcribed RNA representing known viral copy numbers.

### Quantitative image analysis

Briefly, two slides containing BG tissue sections from each animal were stained with antibodies specific for WFS1 and CRYM. No differences in staining intensity were detected between slides for each macaque. A total of ten bright-field sections for each of the nineteen RMs [Groups 1 (*n* = 8), 2 (*n* = 7) and 3 (*n* = 4)] scanned using a Zeiss LSM700 confocal microscope (Carl ZEISS Microscopy, LLC) at 20× objective was imported as digital images into HALO software (Indica Labs) for image quantitation analysis. Since whole/intact BG tissues were used for mRNA and microRNA profiling, we decided to use the area quantification module available on HALO v3.2 (Indica Labs) to quantify WFS1 and CRYM (green signal/Alexa-488) and NeuN (red signal/Alexa-568) fluorescence from the entire BG tissue section. In this new computational method, the artificial intelligence driven software identifies all cells that express WFS1 or CRYM in green, NeuN in red and nuclei in blue and also categorizes the cells based on predefined fluorescence intensity levels. Specifically, the HALO software normalizes the threshold across all images and enables quantification of the number of cells, and relative intensity of fluorescence per cell, of single channel fluorescence (green or red) corresponding to the expression of WFS1, CRYM and NeuN that was intensely expressed in the BG tissue. The output values (total area and average positive intensity) were used to calculate the total WFS1, CRYM and NeuN fluorescent intensity/tissue area. The data were graphed using Prism v9 software (GraphPad software).

### Data analysis and data availability

For RNA-seq analysis, raw reads were first processed through perl scripts to remove reads containing adapter or -N or with a base quality score lower than 20. At the same time, the Q20, Q30 and GC contents of the clean data were calculated. The clean reads were aligned to the genome assembly of *Macaca mulatta* 10 (https://www.ncbi.nlm.nih.gov/genome/215?genome_assembly_id=468623) using TopHat2 [38], and read numbers mapped to each gene were calculated using the HTSeq program [39]. The fragments per kilobase of transcript sequence per million base pairs of each gene were determined by the length of the gene and read counts mapped to this gene. Differential expression analyses of VEH or THC-treated SIV-infected RMs and control groups were performed using the DESeq R package (1.18.0) [40]. Genes with a *p*-value < 0.05 and |log2-fold change| > 0.585 were defined as differentially expressed.

QuantStudio™ run files from all groups were imported into ExpressionSuite software v1.0.2 (Thermo Fisher) and simultaneously analyzed using the uninfected control group as the calibrator to obtain relative gene expression values. The results from the ExpressionSuite software analysis containing five columns (well, sample, detector, task, and *C*_T_ values) were saved as a tab-delimited text file which was later imported and analyzed using the Omics Office StatMiner qPCR analysis software, TIBCO Spotfire, (Perkin Elmer, Waltham, MA). Omics Office StatMiner Software utilizes the comparative Cτ (ΔΔCτ) method to rapidly and accurately quantify relative gene expression across many genes and samples. MiRNA expression data were normalized using the global normalization method and analyzed using the non-parametric Wilcoxon’s rank sum test. In all experiments, the *C*_T_ upper limit was set to 28. A *p*-value of less than 0.05 (< 0.05) was considered significant. OpenArray TaqMan miRNA (https://www.ncbi.nlm.nih.gov/geo/query/acc.cgi?acc=GSE220709) and Novogene RNA-seq (https://www.ncbi.nlm.nih.gov/geo/query/acc.cgi?acc=GSE207518) has been submitted to GEO. Shotgun metagenomic sequencing data (https://dataview.ncbi.nlm.nih.gov/object/PRJNA926599) have been submitted to the SRA.

WFS1 and CRYM immunofluorescence image quantitation data were analyzed using unpaired “*t*” tests (GraphPad, Prism software v5). Shapiro–Wilk and Kolmogorov–Smirnov tests (GraphPad Prism) were used to test for data normality. Both WFS1 and CRYM were found to be normally distributed (no significant p values). RT-qPCR (miR-142-3p) data were analyzed using Mann–Whitney “*U*” test. *Firefly/Renilla* ratios and in vitro endocannabinoid regulation of WFS1 expression was analyzed using one-way ANOVA and post hoc analysis using Tukey’s multiple comparison test.

For metabolite data analysis, after log transformation and imputation of missing values, if any, with the minimum observed value for each compound, Welch’s two-sample *t*-test was used as significance test to identify biochemicals that differed significantly (*p* < 0.05) between experimental groups. Imputation for replacing missing values in the metabolite data analysis was performed by replacing the missing values with its observed minimum (performed after batch normalization). Imputing with the minimum was chosen based on Metabolon’s internal simulation studies comparing this to other methods with regard to the Type I error and power for the two-sample *t*-test. No imputation was performed for RNA-seq, microbiome and microRNA data analysis.

For shotgun metagenomic sequencing, raw sequencing reads were processed to obtain valid reads for further analysis. First, sequencing adapters were removed from sequencing reads using cutadapt v1.9. Secondly, low-quality reads were trimmed by fqtrim v0.94 using a sliding-window algorithm. Thirdly, reads were aligned to the host genome using bowtie2 to remove host contamination. Once quality-filtered reads were obtained, they were de novo assembled to construct the metagenome for each sample by IDBA-UD. All coding regions (CDS) of metagenomic contigs were predicted by MetaGeneMark v3.26. CDS sequences of all samples were clustered by CD-HIT v4.6.1 to obtain unigenes. Unigene abundance for a certain sample was estimated by TPM based on the number of aligned reads by bowtie2 v2.2.0. The lowest common ancestor taxonomy of unigenes were obtained by aligning them against the NCBI NR database by DIAMOND v 0.7.12. To determine the abundance profile of unigenes, differential analysis was carried out at each taxonomic level by Fisher’s exact test. Unigenes with a *p*-value < 0.05 and |log_2_-fold change| > 0.585 were defined as differentially expressed.

Although hypothesis-driven, we have reported genes, microRNA, plasma metabolites, and bacterial taxa that showed statistical significance at the level of *p* < 0.05 without applying multiple comparison testing (False discovery rate). It is important to note that significant changes in mRNA expression (*p* and *q* values < 0.05) do not always guarantee concurrent changes in protein expression. Similarly, differential miRNA expression does not provide information about their functional significance. For these reasons, we used the dual-labeled immunofluorescence technique to confirm changes in protein expression of two genes of interest (WFS1 and CRYM) in the same BG tissues. In addition, we used the RT-qPCR assay to confirm miR-142-3p expression (OpenArray data) and then followed up with dual luciferase and miRNA overexpression assays to validate *WFS1* as a direct target of miR-142-3p.

## Results

### Plasma, basal ganglia and colon viral loads, CD4^+^ and CD8^+^ T cell status, and brain histopathology

All VEH/SIV and THC/SIV RMs had substantial plasma (0.02 × 10^6^ to 3.0 × 10^9^/mL), basal ganglia (0.01 × 10^6^ to 4 × 10^10^/mg total RNA) and colon (1 × 10^6^ to 9.36 × 10^9^/mg total RNA) viral loads (Table 1). No difference in plasma viral RNA copies were detected between VEH/SIV and THC/SIV RMs. At least three (GV60, HT48, IA83) THC/SIV RMs had substantially high brain viral loads (Table 1). Longitudinal peripheral blood viral loads, CD4^+^ and CD8^+^ T cell dynamics from the four VEH/SIV (JH47, JR36, JD66, IV95) and THC/SIV (JI45, JT80, IV90, JC85) RMs were previously published [24]. Marked depletion of intestinal and peripheral blood CD4^+^ T cells were confirmed in both groups [24]. A significant decline in CD4^+^ T cell percentages at 2 weeks post-SIV infection, was paralleled by a concomitant increase in CD8^+^ T cell percentages [24]. Histopathological analysis revealed the presence of granulomatous encephalitis (syncytial cells) in one macaque each in the VEH/SIV and THC/SIV group (Table 1). BG viral loads were not available from GA19, FT11, and HB31 (Table 1). No statistically significant differences in BG and colon viral loads were detected between VEH/SIV and THC/SIV RMs (Additional file 1: Fig. S1).

### Genes associated with neuroinflammation driven by type-I interferon responses are markedly upregulated in BG of VEH/SIV but not in THC/SIV RMs

**Fig. 1:**
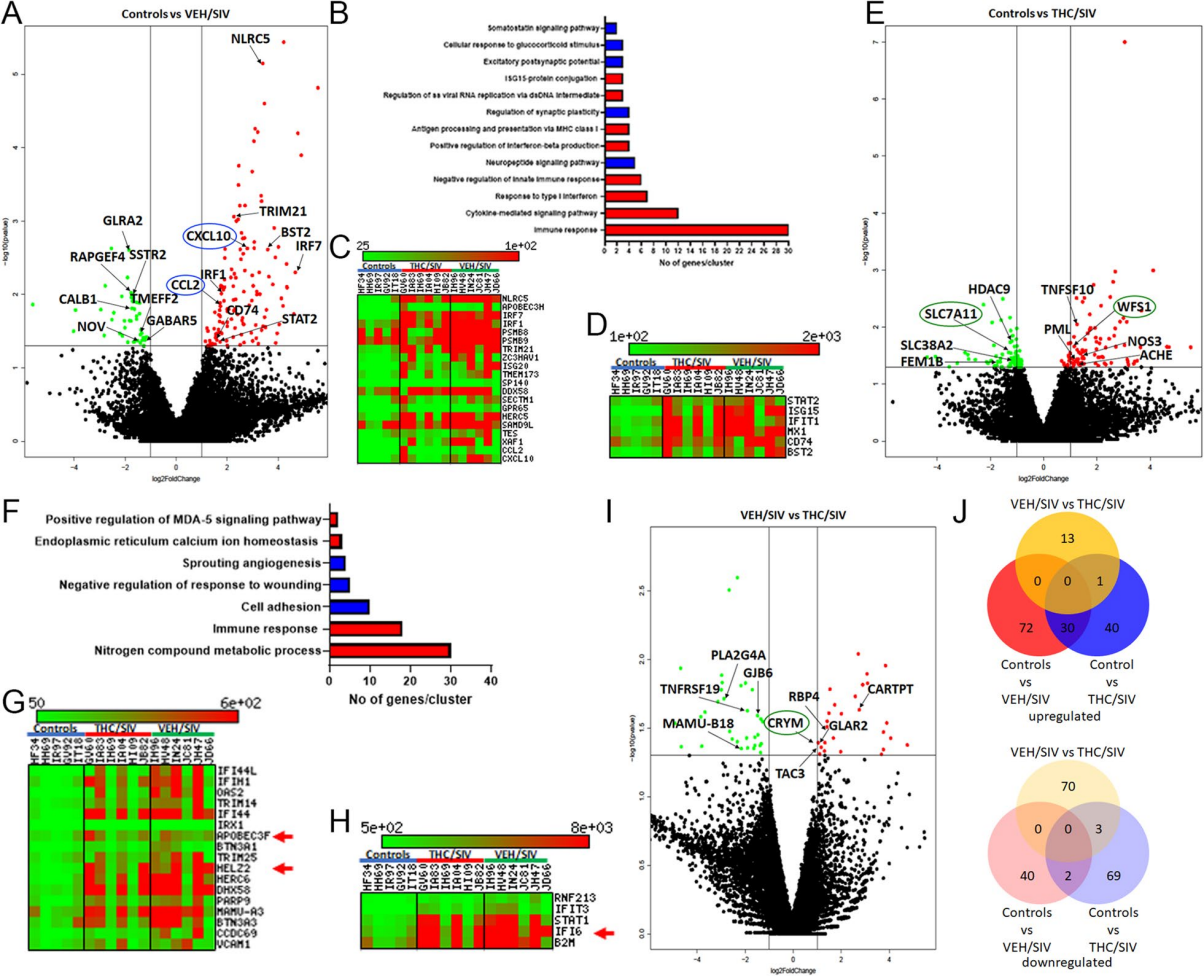
Long-term, low-dose THC administration reduced proinflammatory gene expression in basal ganglia of chronically SIV-infected RMs. Volcano plot shows the relationship between fold-change (*X*-axis) and statistical significance (*Y*-axis) of differentially expressed mRNAs in VEH/SIV (**A**) and THC/SIV (**E**) RMs relative to controls and in THC/SIV relative to VEH/SIV RMs (**I**). The vertical lines in (**A**, **E**, **I**) correspond to 2.0-fold up and down, respectively, and the horizontal line represents *p* ≤ 0.05. The negative log of statistical significance (*p*-value) (base 10) is plotted on the *Y*-axis, and the log of the fold change base (base 2) is plotted on the *X*-axis. Notable differentially expressed mRNAs are shown in the volcano plots. Gene Ontology functional annotation cluster analysis of upregulated (red bars in **B** and **F**) and downregulated (blue bars in **B** and **F**) genes in VEH/SIV and THC/SIV RMs, respectively, relative to controls. Number of genes represented in each cluster in VEH/SIV (**B**) and THC/SIV (**F**) relative to controls. Heat maps show interferon stimulated and proinflammatory genes that showed statistically significant upregulation exclusively in BG of VEH/SIV (**C**, **D**) and those that showed higher read counts and fold change in VEH/SIV than THC/SIV compared to uninfected control RMs (**G**, **H**). Venn diagrams showing the number of differentially expressed annotated mRNAs that are unique to each group or overlapped between the three groups (**J**)

To uncover the molecular pathogenesis of HIV/SIV-induced neurological disease/dysfunction and its modulation by phytocannabinoids, we performed transcriptomic profiling of BG samples collected at necropsy using RNA-seq. Relative to uninfected controls, 145 genes were found to be significantly upregulated and differentially expressed (DE) in BG of VEH/SIV RMs. Out of these, we successfully annotated 102 genes using DAVID (Fig. 1A). Gene enrichment analysis using gene ontology (GO) showed differential enrichment of biological functions involved in ISG15–protein conjugation (*n* = 3) (*p* = 5.36 × 10^–06^) [41], response to type-I IFN (*n* = 7) (*p* = 8.61 × 10^–11^), regulation of single stranded viral RNA replication via double stranded DNA intermediate (*n* = 3) (*p* = 3.46 × 10^–05^), positive regulation of interferon-beta production (*n* = 4) (*p* = 1.50 × 10^–05^), negative regulation of innate immune response (*n* = 6) (*p* = 1.27 × 10^–07^), antigen processing and presentation of peptide antigen via MHC class I (*n* = 4) (*p* = 7.03 × 10^–03^), cytokine-mediated signaling pathway (*n* = 12) (*p* = 3.49 × 10^–06^) and immune response (*n* = 30) (*p* = 2.35 × 10^–20^) (red bars in Fig. 1B). Heatmaps in Fig. 1C, D show notable type-I IFN stimulated and other proinflammatory genes that were significantly upregulated exclusively in BG of VEH/SIV RMs. Additional file 2: Table S1 lists the full names, read counts, and fold change of 28 key IFN stimulated and other neuroinflammatory genes that showed statistically significant upregulation exclusively in BG of VEH/SIV RMs. These included *NLRC5* (negative regulator of NFκB activation and type-I IFN signaling) [42], *CXCL10* and *CCL2* (neutrophil and monocyte chemoattractant) [43, 44], *STAT2* (type-I IFN signaling), *BST2/ISG15/ISG20*, *IFIT1*, *HERC5*, *DDX58*, *MX1* (IFN induced anti-viral proteins), *IRF1* and* IRF7* (innate and adaptive immune response) [45, 46], *TRIM21* (negative regulator of interferon beta production), *CD74* (immune response and receptor for macrophage migration inhibitory factor) [47], *LGALS3BP* and *SAMD9L* (anti-viral response), *PTGES* (proinflammatory PGE2 synthesis), *PARP14* (reducing STAT1 phosphorylation and inhibitor of proinflammatory cytokine production) [48], and *S100A4* (neuronal plasticity and protection) [49].

Unlike the upregulated genes, fewer genes (*n* = 49) showed statistically significant downregulation in BG of VEH/SIV compared to uninfected control RMs (Fig. 1A). Out of these, 42 genes were annotated. Gene enrichment analysis using GO showed differential enrichment of biological functions involved in somatostatin signaling pathway (*n* = 2) (*p* = 4.59 × 10^–05^), cellular response to glucocorticoid stimulus (*n* = 3) (*p* = 8.87 × 10^–06^), excitatory postsynaptic potential (*n* = 3) (*p* = 5.80 × 10^–05^), neuropeptide signaling pathway (*n* = 5) (*p* = 1.80 × 10^–07^) and regulation of synaptic plasticity (*n* = 4) (*p* = 3.30 × 10^–05^) (blue bars in Fig. 1B). Using supervised analysis, we identified important genes linked to neuronal function/survival, neurogenesis, synaptic plasticity, neurotransmission and learning/memory (*TMEFF2* [50, 51], *GDA* [52], *FEZF2* [53]), reducing neuronal excitability (*GLRA2*) [54], promoting neurogenesis, neuronal migration, development, maturation and axonal outgrowth (*RAPGEF4* [55, 56], *SSTR2* [57, 58]), glutamate receptor activation/deactivation and localization (*CNIH3*) [59], synaptic vesicle trafficking and neurotransmitter release (*SNCA*) [60], retinol transport across the BBB (*RBP4*), spatial learning and cognition (*NPY1R*)[61], neuronal cytosolic calcium homeostasis (*CALB1*)[62], inhibition of GABA-mediated neurotransmission (*GABRA5*), and reduction of monocyte adhesion and negative regulation of endothelial inflammation (*NOV*) [63] that were markedly downregulated in BG of VEH/SIV RMs (Fig. 1A). *TMEFF2*, *SSTR2*, *RAPGEF4*, *SNCA*, and *NOV* are noteworthy for their established roles in neuronal survival, neurogenesis, synaptic vesicle transmission and inhibition of monocyte adhesion that were significantly downregulated only in BG of VEH/SIV RMs. Additional file 2: Table S2 lists the full names, read counts, and fold change of the 34 differentially downregulated genes in BG of VEH/SIV RMs.

### Long-term THC significantly enhanced the expression of Wolfram syndrome-1 (WFS1) [negative regulator of endoplasmic reticulum (ER) stress] and Ketimine reductase/Crystallin mu (CRYM) [anti-oxidant] in BG of chronically SIV-infected RMs

In contrast to VEH/SIV RMs, fewer genes (*n* = 99) were significantly upregulated in BG of THC/SIV RMs compared to uninfected controls (Fig. 1E). Out of these, 71 genes were annotated. This represented a 1.4-fold reduction (102 vs 71) in the total number of upregulated genes in THC/SIV compared to VEH/SIV RMs. Interestingly, gene enrichment analysis using GO showed differential enrichment of biological functions involved in positive regulation of MDA-5 signaling pathway (*n* = 2) (*p* = 2.14 × 10^–2^), ER calcium ion homeostasis (*n* = 3) (*p* = 4.5 × 10^–5^), nitrogen compound metabolic process (*n* = 30) (*p* = 1.09 × 10^–4^), and immune response (*n* = 18) (*p* = 2.09 × 10^–11^) (red bars in Fig. 1F). While the immune response associated gene expression cluster overlapped with VEH/SIV RMs, fewer genes were present in the cluster detected in THC/SIV RMs (30 vs 18). Key genes linked to negative regulation of unfolded protein response (UPR) and neuronal survival (*WFS1*) [64, 65], apoptosis of HIV-infected macrophages (*TNFSF10*) [66], HIV transcriptional repression (*PML*) [67], neurotransmission and antimicrobial function (*NOS3*) [68], and termination of cholinergic transmission (*AChE*) were significantly upregulated in BG of THC/SIV RMs. Additional file 2: Table S3 lists the full names, read counts, and fold change of 31 key differentially upregulated genes in BG of THC/SIV RMs.

Compared to controls, 85 genes were downregulated in BG of THC/SIV RMs. Out of these, 73 genes were successfully annotated. Gene enrichment analysis using gene ontology showed differential enrichment of biological functions involved in cell adhesion (*n* = 10) (*p* = 7.09 × 10^–6^), negative regulation of response to wounding (*n* = 5) (*p* = 1.02 × 10^–6^), and sprouting angiogenesis (*n* = 4) (*p* = 1.22 × 10^–5^) (blue bars in Fig. 1F). A select number of genes with known roles in synapse formation and neuronal migration (*SEMA3E*, *SLIT2* [69], *TCF4* [70]), apoptosis (*FEM1B*), cell adhesion (*FAT4*), neuroinflammation (*HDAC9*) [71, 72], stress responses (glucocorticoid receptor or *NR3C1*), serine/glutamine transport (*SLC38A2*) [73], and glutamate transport and excitotoxicity (*SLC7A11*) [74, 75] were significantly downregulated in BG of THC/SIV RMs. Additional file 2: Table S4 lists the full names, read counts and fold change of 35 differentially downregulated genes in BG of THC/SIV RMs. Heatmaps (Fig. 1G, H) show expression patterns of 22 differentially upregulated type-1 IFN induced and immune response genes in BG of VEH/SIV and THC/SIV relative to uninfected control RMs. Note that with the exception of three (red arrowheads in Fig. 1G, H), the read counts and fold change for all genes are markedly higher in BG of VEH/SIV RMs (Additional file 2: Table S5). Although multinucleated giant cells were detected in the brain and other organs of HB31 and GV60 (THC/SIV) at necropsy (Table 1), only HB31 showed signs of AIDS progression (> 20% weight loss, head tilt, loss of appetite, etc.), which may be partly attributed to the effects of THC.

When comparing THC/SIV and VEH/SIV RMs, genes associated with apoptosis (*TNFRSF19*), prostaglandin synthesis (*PLA2G4A*) [76], immune response (*Mamu-B18*), glucose and lactate transport (*GJB6*) [77], neuronal differentiation, neurite outgrowth and tissue repair (*EMP1*, *FGFBP1*) [78, 79] were downregulated in THC/SIV RMs. Most strikingly, *CRYM* (a ketimine reductase that generates the anti-oxidant, pipecolate) [80], *CARTPT*, *RBP4*, *GLRA2* and *TAC3* were significantly upregulated in BG of THC/SIV RMs (Fig. 1I). The Venn diagram analysis of annotated genes confirmed a markedly higher number of upregulated mRNAs in the VEH/SIV compared to THC/SIV group relative to the uninfected control group (Fig. 1J). Interestingly, the opposite trend was observed with downregulated mRNAs, where the THC/SIV group showed significantly higher number of genes compared to VEH/SIV relative to the uninfected control group (Fig. 1J). The Venn diagram analysis showed that the number of differentially expressed genes in the VEH/SIV and THC/SIV groups relative to uninfected controls overlapped by only 27% (30 genes) and less than 2% (2 genes) for up- and downregulated genes, respectively. These findings clearly demonstrate the divergence of transcriptomic responses in VEH/SIV versus THC/SIV (72 and 40 up-, and, 40 and 69 downregulated genes in the two groups, respectively) compared to uninfected controls.

Furthermore, a unique set of 13 upregulated and 70 downregulated genes were identified in the THC/SIV when compared to the VEH/SIV group. Gene enrichment analysis of the 12 upregulated genes, using gene ontology (GO), showed differential enrichment of biological functions involved in neuropeptide signaling pathway (*n* = 4; *GLRA2*, *TAC3*, *CARTPT*, *GRP*) (*p* = 3.31 × 10^–08^). Similarly, GO analysis of the 70 downregulated genes showed differential enrichment of biological functions mainly involved in cytoskeleton organization (*n* = 16) (*p* = 3.46 × 10^–06^) that included about nine keratin proteins. In tune with the involvement of keratins described in chronic alcohol induced neurotoxicity [81], our findings identify a role for keratins in HIV/SIV neuropathogenesis and the ability of THC to suppress its aberrant expression. Despite not being grouped into established functional clusters, several notable genes associated with immune response (*TNFRSF19*, *MAMU-DOB*, *MAMU-B18*, *ARG1*, *IL36A*, *KLK12*, *PLA2G4A*, and *ANXA8*) were significantly downregulated in BG of THC/SIV RMs. The activation of neuropeptide signaling pathway and the downregulation of a select list of inflammation relevant and keratin genes likely represents a specific response to THC treatment independent of HIV/SIV infection as only four genes overlapped. Additional file 2: Table S6A and 6B lists the full names, read counts, and fold change of unique differentially up- (*n* = 13) and down-regulated (*n* = 70) genes, respectively, in BG of THC/SIV relative to VEH/SIV RMs.

We want to emphasize that the goal of the study is to determine the impact of THC administration in mitigating HIV/SIV-induced chronic inflammation and not the overall effects of THC outside of inflammatory diseases. For this reason, an additional group of THC-treated SIV-uninfected RMs was not included. Despite SIV infection, *WFS1* expression was significantly elevated only in BG of THC/SIV RMs (Fig. 1E), compared to uninfected controls. When compared to VEH/SIV, *CRYM* expression was significantly elevated only in THC/SIV RMs. These findings highlight the specific effects of THC in the BG during chronic HIV/SIV infection.

### Wolfram syndrome 1 (WFS1) and Crystallin mu (CRYM) protein expression is significantly increased in BG neurons of THC/SIV RMs

Because of their ability to reduce and protect against ER and oxidative stress, two pathogenic events known to drive neuronal loss/dysfunction, we next focused on *WFS1* [64, 82] and *CRYM* [83, 84] and determined whether significantly high mRNA levels in BG of THC/SIV RMs were paralleled at the protein level and which cell types in the BG contributed to its differential expression.

**Fig. 2:**
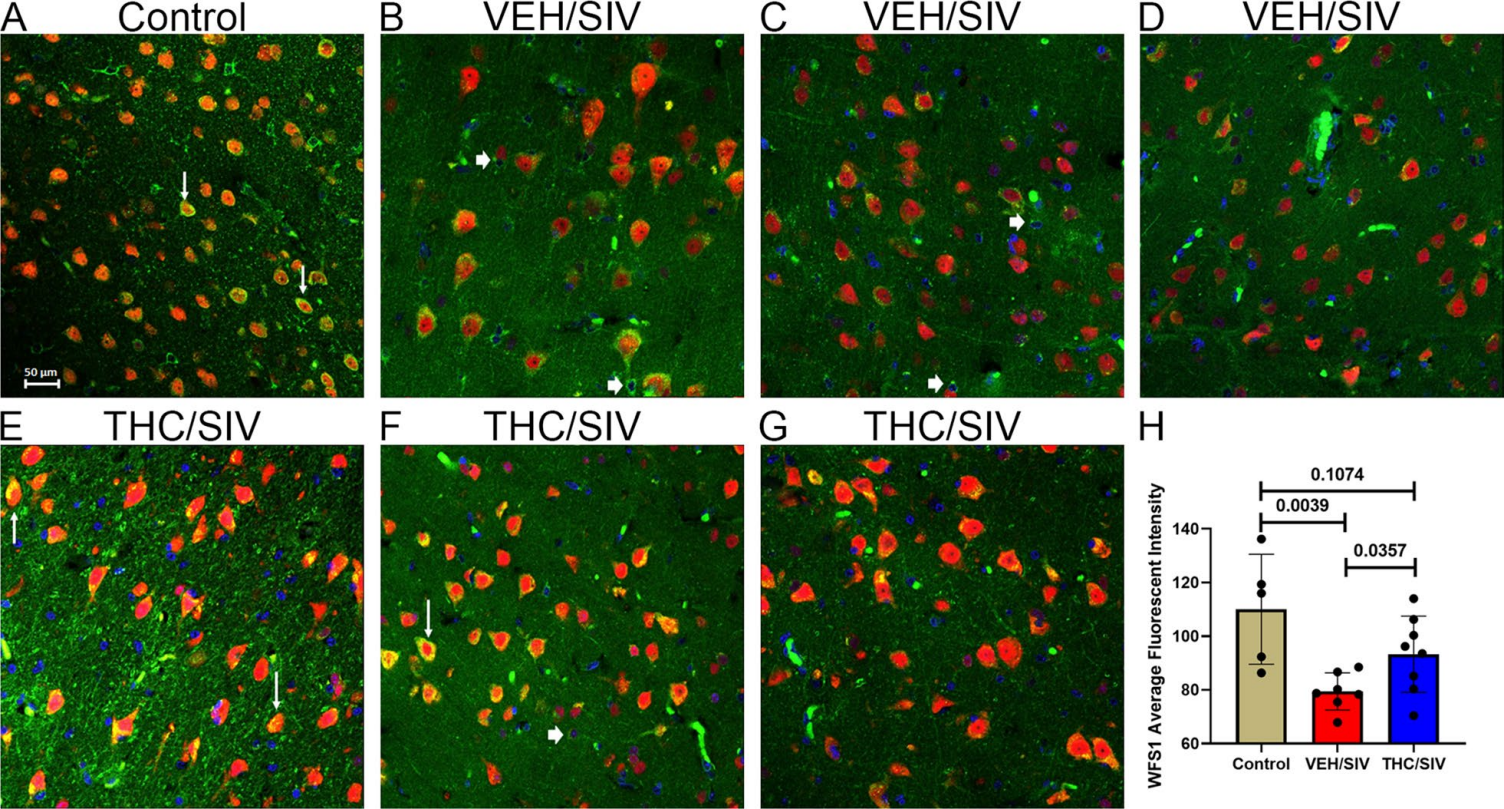
Chronic THC administration increased WFS1 protein expression in the basal ganglia of chronically SIV-infected RMs. Basal ganglia tissues of uninfected control (**A**), VEH/SIV (**B**–**D**), and THC/SIV RMs (**E**–**G**) were immunostained for WFS1 (green), NeuN (red), and DAPI for nuclear staining (blue). Note the significantly decreased WFS1 (**B**–**D**) staining in the BG of VEH/SIV RMs. In contrast, WFS1 (**E**–**G**) staining is intense in NeuN^+^ neurons in the BG of THC/SIV RMs. Representative immunofluorescence images were captured using a Zeiss confocal microscope at 20X magnification. Yellow staining (**A**, **E**, **F**) indicates colocalization of WFS1 to NeuN^+^ neurons (white arrow). A few NeuN^−^ cells expressing WFS1 protein were also detected (**B**, **C**, and **F**, white arrowhead). Quantitation of WFS1 (**H**) signal intensity was performed using Halo software. Differences in WFS1 signal intensity between groups were analyzed using unpaired “*t*” tests after confirming data assumptions (normal distribution) employing the Prism v9 software (GraphPad software). A *p*-value of < 0.05 was considered significant

Using the neuronal marker NeuN, WFS1 protein expression was localized predominantly to BG neurons (white arrows in Fig. 2A). However, WFS1 protein expression was also detected in a few NeuN-negative cells (white arrowhead in Fig. 2B, C, and F). Although no differences in *WFS1* mRNA expression were detected in BG of VEH/SIV RMs compared to uninfected controls, WFS1 protein expression (staining intensity) was significantly reduced in BG neurons of VEH/SIV (*n* = 7) (Fig. 2B–D) relative to both THC/SIV (*n* = 8) (Fig. 2E–G) and uninfected control (*n* = 5) RMs (Fig. 2A). WFS1 protein expression was significantly higher in the BG of THC/SIV RMs (Fig. 2E–G). Quantitation of WFS1 protein expression exclusively in NeuN-positive neurons confirmed significantly elevated WFS1 protein expression in BG of THC/SIV relative to VEH/SIV RMs (Fig. 2H).

**Fig. 3:**
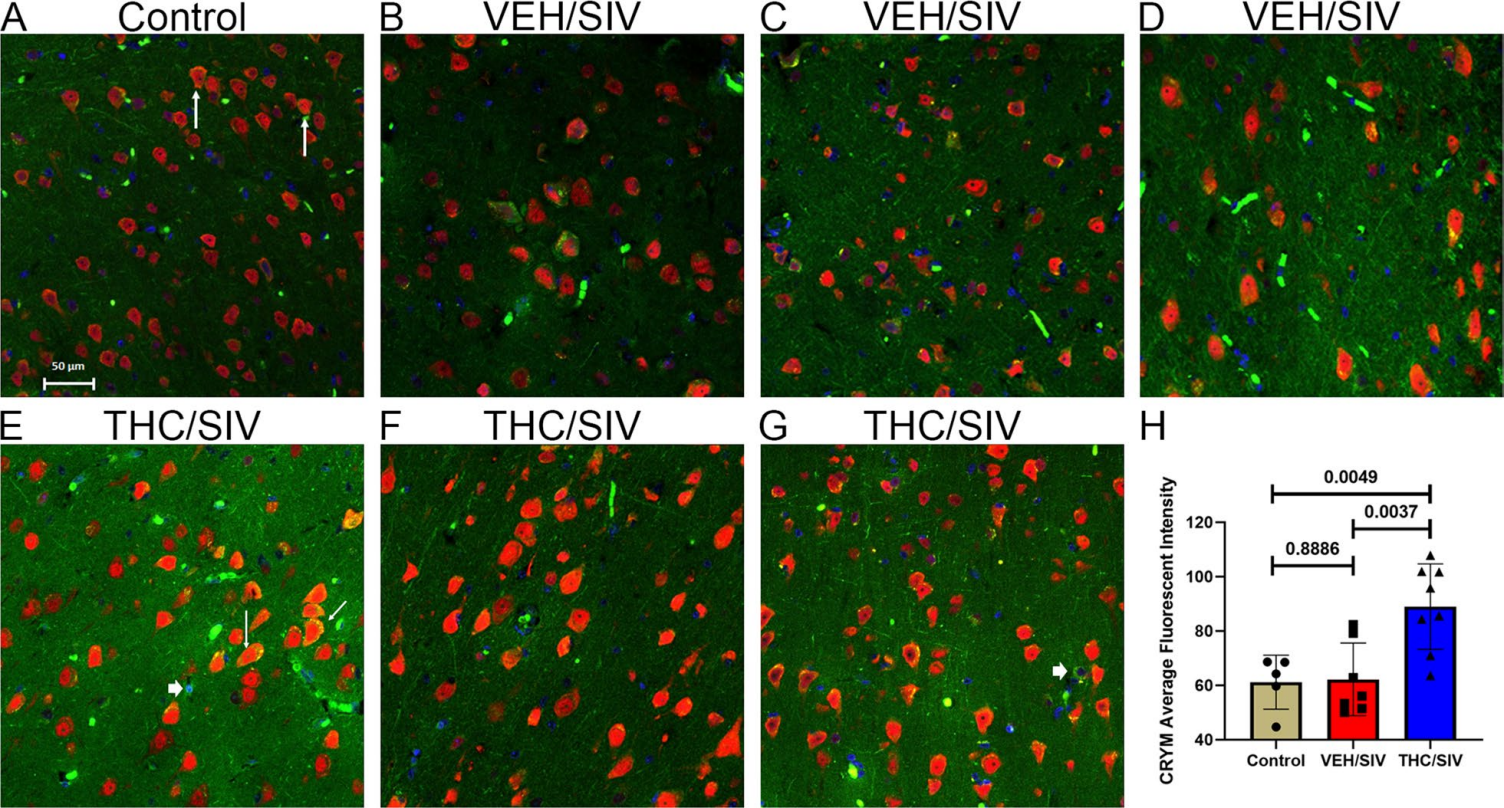
Chronic THC administration increased CRYM protein expression in the basal ganglia of chronically SIV-infected RMs. Basal ganglia tissues of uninfected control (**A**), VEH/SIV (**B**–**D**), and THC/SIV RMs (**E**–**G**) were immunostained for CRYM (green), NeuN (red), and DAPI for nuclear staining (blue). Note the significantly increased CRYM staining in NeuN^+^ neurons in the BG of THC/SIV (**E**–**G**) compared to VEH/SIV (**B**–**D**) and uninfected control RMs (**A**). A few NeuN^−^ cells expressing CRYM protein were also detected (**E** and **G**, white arrowhead). Representative immunofluorescence images were captured using a Zeiss confocal microscope at 20X magnification. Yellow staining (**A**, **E**) indicates colocalization of CRYM to NeuN^+^ neurons (white arrow). Quantitation of CRYM (**H**) signal intensity was performed using Halo software. Differences in CRYM signal intensity between groups were analyzed using unpaired “*t*” tests after confirming data assumptions (normal distribution) employing the Prism v9 software (GraphPad software). A *p*-value of < 0.05 was considered significant

The significantly high *CRYM* mRNA expression was equally intriguing as it also functions as a ketimine reductase that uses either NADH or NADPH as a cofactor to catalyze the reduction of the imine bond in 1-piperideine-2-carboxylate to produce the anti-oxidant and neuroactive l-pipecolate [80]. Like WFS1, strong CRYM protein expression was detected mainly in BG NeuN-positive neurons and a few NeuN-negative cells (white arrowhead in Fig. 3E, G) in uninfected control (Fig. 3A), VEH/SIV (Fig. 3B–D) and THC/SIV (Fig. 3E–G) RMs. Consistent with RNA-seq data, image quantification confirmed significantly high CRYM protein expression in BG neurons of THC/SIV compared to VEH/SIV RMs (Fig. 3H). Interestingly, THC/SIV RMs also showed significantly higher CRYM protein expression in BG neurons compared to uninfected controls (Fig. 3H). Finally, we want to emphasize that while both genes did not pass the false discovery rate (adjusted *p*-value), similar to RNA-seq data, significant differences in WFS1 and CRYM protein expression were detected between treatment groups. Image quantitation data for both WFS1 and CRYM were analyzed using the unpaired “t” test. The QQ plots showing normal distribution of data for both datasets are provided in Additional file 1: Fig. S2.

### miR-142-3p post-transcriptionally regulates WFS1 protein expression

**Fig. 4:**
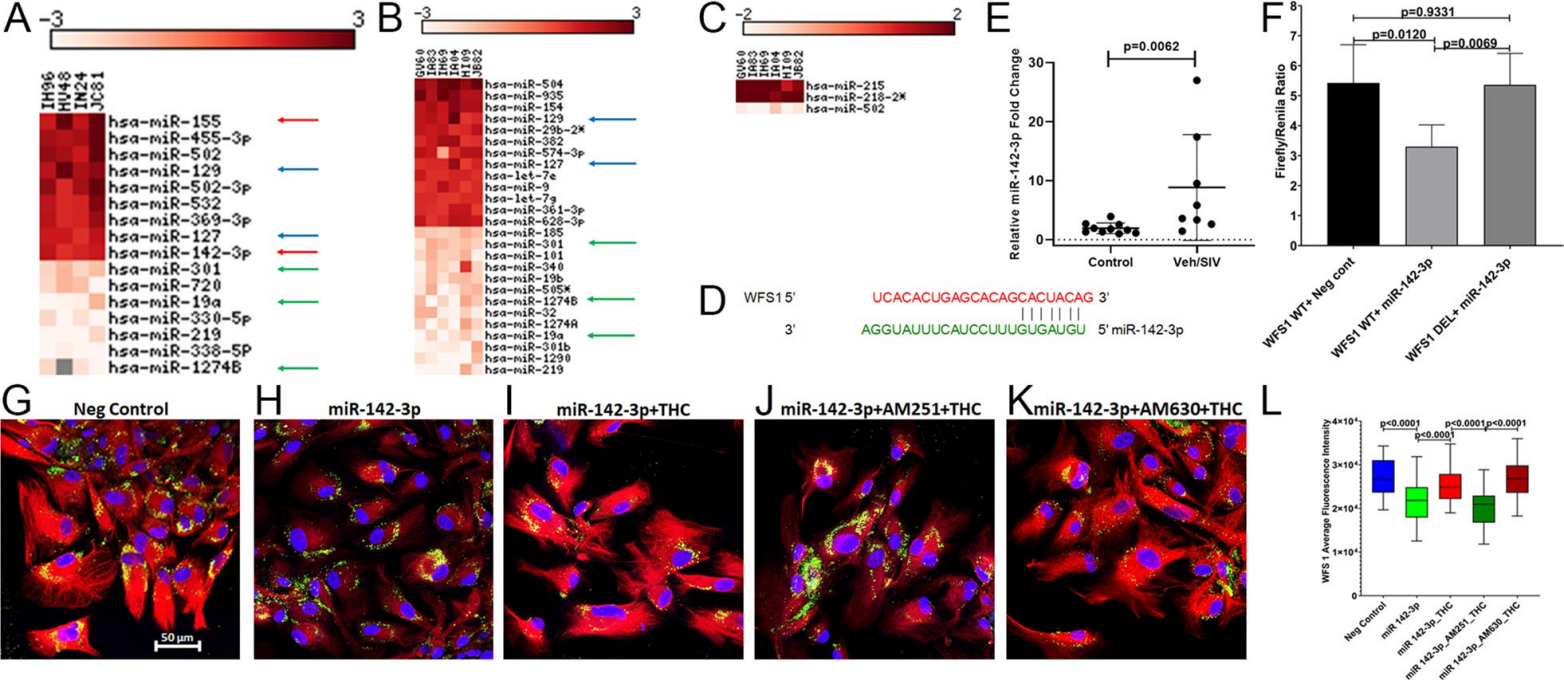
THC can counteract the post-transcriptional silencing of *WFS1* by miR-142-3p. The heat map shows all differentially expressed (*p* ≤ *0.05*) miRNAs in the BG of VEH/SIV (**A**) and THC/SIV (**B**) relative to uninfected controls RMs and in VEH/SIV compared to THC/SIV RMs (**C**). MiRNA species originating from the opposite arm of the precursor are denoted with an asterisk (*). Red arrows (**A**) indicate inflammation-associated miRNAs differentially upregulated in BG of VEH/SIV RMs. Blue and green arrows indicate miRNAs commonly up and downregulated in BG of VEH/SIV (**A**) and THC/SIV (**B**) RMs, respectively. MiRNA–mRNA duplex showing a single miR-142-3p binding site on the RM *WFS1* (**D**) mRNA 3’ UTR. RT-qPCR validation of miR-142-3p expression in BG of VEH/SIV relative to uninfected control RMs (**E**). Luciferase reporter vectors containing a single highly conserved miR-142-3p (**F**) binding site on the RM *WFS1* mRNA 3′ UTR or the corresponding construct with the binding sites deleted (WFS1 DEL) were co-transfected into HEK293 cells with 30 nM miR-142-3p or negative control mimic. *Firefly* and *Renilla* luciferase activities were detected using the Dual-Glo luciferase assay system 96 h after transfection. Luciferase reporter assays were performed thrice in six replicate wells (**F**). Representative immunofluorescence images showing the expression of WFS1 (red) protein at 96 h post-transfection of HCN2 neuronal cells with 30 nM LNA-conjugated FAM-labeled negative control (**G**) or miR-142-3p (green) (**H**) mimics and images were quantitated (**L**). miR-142-3p transfected HCN2 cells were treated with DMSO (**H**), THC (**I**), AM251 + THC (**J**), AM630 + THC (**K**) 96 h post-transfection. Cells were fixed and stained after 18 h and the expression of WFS1 (red) protein and nuclear staining using DAPI (blue) were quantitated (**L**). Experiments were performed in triplicate wells using 3 µM of THC, 10 µM of AM251/AM630, and repeated thrice. *Firefly/Renilla* ratios and immunofluorescence data were analyzed using one-way ANOVA followed by Tukey’s multiple comparison post hoc test. A *p*-value of < 0.05 was considered significant

To identify potential post-transcriptional mechanisms regulating *WFS1* mRNA expression, we profiled miRNA expression in a subset of VEH/SIV (*n* = 4) but all THC/SIV (*n* = 6) RMs used for RNA-seq studies. Unfortunately, we did not have sufficient amounts of total RNA from two animals (JH47 and JD66) in the VEH/SIV group. Relative to uninfected controls (*n* = 4), 16 (9 up and 7 down) (Fig. 4A) and 26 (13 up and 13 down) (Fig. 4B) miRNAs were found to be DE in VEH/SIV and THC/SIV RMs, respectively. Relative to VEH/SIV RMs, three (2 up and 1 down) miRNAs were DE in BG of THC/SIV RMs (Fig. 4C). A notable finding was the significant upregulation in BG of miR-142-3p, and miR-155, two miRNAs found to be upregulated in numerous neuroinflammatory brain disorders including HIV [85–87] (red arrows in Fig. 4A). Interestingly, both miR-142-3p and miR-155 did not show statistically significant upregulation in BG of THC/SIV RMs. At least, five miRNAs were commonly up (miR-129 and miR-127) (blue arrows in Fig. 4A, B) and downregulated (miR-301, miR-19a and miR-1274B) (green arrows in Fig. 4A, B) in BG of VEH/SIV and THC/SIV, respectively, relative to control RMs.

Using the TargetScan 7.2 algorithm [36], perfect miRNA seed nucleotide matches (miRNA nucleotide positions 2–7) were identified for miR-142-3p on the 3’ mRNA UTR of *WFS1* (Fig. 4D). Moreover, miR-142-3p dysregulation and its high expression in neurons has been previously demonstrated in the brains of SIV-infected RMs [86]. Using RT-qPCR, we further confirmed significant upregulation of miR-142-3p (Fig. 4E) in the BG of VEH/SIV RMs. Transfection of HEK293 cells with 30 nM LNA-miR-142-3p mimic significantly reduced firefly/renilla ratios suggesting that miR-142-3p can regulate *WFS1* expression by directly binding to its 3′ UTR and exerting post-transcriptional repression (Fig. 4F).

### THC countered miR-142-3p-mediated suppression of WFS1 protein expression in HCN2 neuronal cells and potentially basal ganglia neurons through a cannabinoid receptor-1 (CB1R)-mediated mechanism

Next, we overexpressed FAM-labeled LNA-conjugated miR-142-3p mimics in HCN2 cells to determine its regulatory impact on WFS1 protein expression. HCN2 is a human cerebrocortical neuronal cell line and has been used widely for in vitro electro neurophysiological studies. Based on the expression of tubulin, glutamate and GABA, we found HCN2 to be a well-suited neuronal cell line for in vitro validation of our in vivo findings in BG neurons. Confirming the results of the luciferase reporter assay (Fig. 4F), miR-142-3p overexpression significantly decreased WFS1 protein expression (Fig. 4H, L) in HCN2 cells compared to cells transfected with the negative control mimic (Fig. 4G, L), thus providing a potential miRNA-mediated post-transcriptional mechanism regulating BG WFS1 protein expression in chronic HIV/SIV infection. Since long-term THC significantly increased WFS1 protein expression in BG neurons of chronically SIV-infected RMs (Fig. 2H), we next determined whether THC could override the suppressive effects of miR-142-3p on WFS1 protein expression. Treatment of miR-142-3p transfected HCN2 cells (96 h post-transfection) with 3 µM THC significantly increased WFS1 protein expression after 24 h (Fig. 4I, L). The 3 µM THC concentration was determined using a dose response study published previously [29]. After confirming protein expression of both CB1R and CB2R (Additional file 1: Fig. S3A, B), HCN2 cells were preincubated with 10 µM of the CB1R antagonist AM251 or the CB2R antagonist AM630 (Tocris Bioscience, Minneapolis, MN) for 1 h followed by 3 µM THC to identify the specific cannabinoid receptor (CB1R or CB2R) involved in transducing the effects of THC on WFS1 protein expression. The optimization of 10 µM AM251 and AM630 dose was determined previously [29]. As evident in Fig. 4J, L, blockade of CB1R with AM251 significantly (*p* < 0.0001) decreased the ability of THC to increase WFS1 protein expression compared to miR-142-3p transfected and THC-treated cells (*p* < 0.0001) (Fig. 4I). In contrast, blockade of CB2R with AM630 resulted in significantly increased WFS1 protein expression (Fig. 4K, L) considerably exceeding that observed after blockade of CB1R using AM251 (*p* < 0.0001) (Fig. 4J). Overall, CB2R blockade significantly enhanced THC’s ability to override the post-transcriptional suppressive effects of miR-142-3p on WFS1 protein expression potentially via increased binding and signaling through CB1R.

### Long-term THC administration significantly increased plasma concentrations of the anti-oxidant pipecolate, entero-, cardio- and neuroprotective endocannabinoids, glycerophospholipids and indole-3-propionate in chronically SIV-infected RMs

**Fig. 5:**
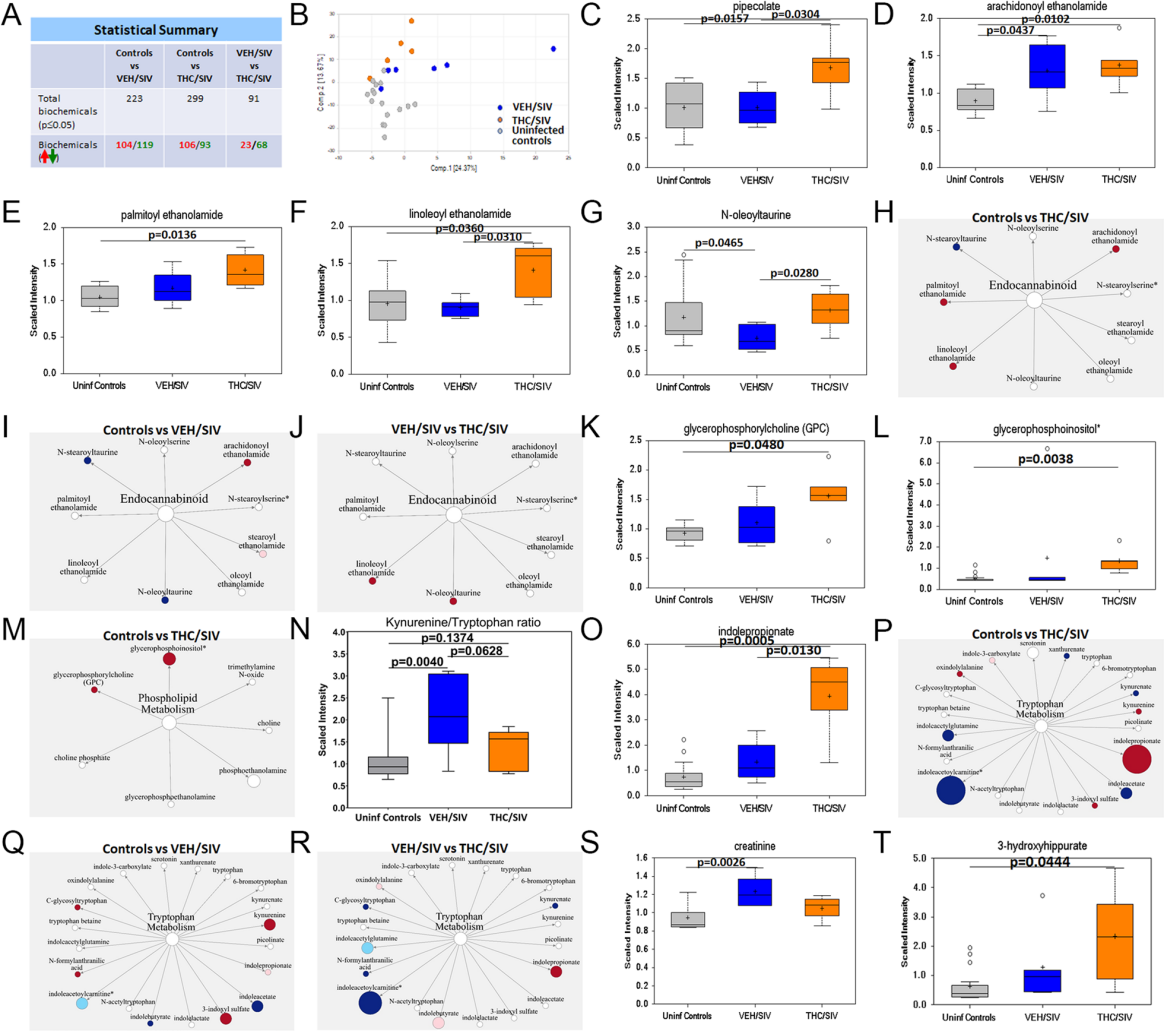
THC administration increased pipecolate, endocannabinoids, endocannabinoid-like, glycerophospholipid, and indole-3-propionate levels. Statistical summary (**A**) and PCA plot (**B**) of metabolites detected in plasma of uninfected and SIV-infected RMs administered VEH or THC. Plasma levels of pipecolate (**C**), endocannabinoids (**D**), endocannabinoid-like (**E**–**G**), glycerophospholipid (**K**, **L**), kynurenine–tryptophan ratios and indole-3-propionate (**N**, **O**), creatinine (**S**), and 3-hydroxyhippurate (**T**) in VEH/SIV (*n* = 6) or THC/SIV (*n* = 5) RMs at 5 MPI and uninfected control RMs (*n* = 16). Red and green arrows and numbers (**A**) represent increased and decreased metabolites, respectively. Pathway figures show endocannabinoids (**H**–**J**), phospholipids (**M**), and tryptophan metabolites (**P**–**R**) detected in plasma of VEH/SIV or THC/SIV relative to uninfected control RMs or THC/SIV relative to VEH/SIV RMs. Red and blue circles represent significantly increased and decreased metabolites, respectively. Light blue and pink circles represent increased and decreased metabolites, respectively, with a *p*-value between 0.05 and 0.09. Size of the circle indicates relative abundance. Open circles represent outliers for that particular metabolite. A *p*-value of < 0.05 was considered significant

To determine if increased *CRYM* mRNA and protein expression was associated with elevated l-pipecolate levels, we performed metabolomic profiling of plasma samples collected from VEH/SIV (*n* = 6), THC/SIV (*n* = 6) at 5 MPI and a separate cohort of uninfected control (*n* = 16) RMs. We want to note that macaque #IH69 from the THC/SIV group (Table 1) met Metabolon’s threshold of the definition of an outlier as this macaque was found to be an outlier for 40% of the metabolites. As a result, the total number of THC/SIV RMs used for metabolomic profiling was reduced to five. We previously reported confirming the presence of THC, its metabolite, THC carboxylic acid and the secreted form THC carboxylic acid glucuronide in the plasma of THC/SIV RMs used in the current study [29]. The table in Fig. 5A shows that a higher number of metabolites were detected in the THC/SIV group (*n* = 299) than the VEH/SIV group (*n* = 223) relative to uninfected controls. The principal component analysis showed some observed separation between uninfected controls and samples collected at 5 MPI (both VEH/SIV and THC/SIV) (Fig. 5B). Other notable observations are the THC/SIV samples were the most different from the uninfected control samples. Consistent with increased *CRYM* gene and protein expression in BG, we detected significantly (*p* < 0.05) higher pipecolate levels in plasma of THC/SIV compared to both VEH/SIV and uninfected control RMs (Fig. 5C).

Since inflammation alters endocannabinoid levels [88, 89], we next focused on plasma levels of endogenous cannabinoids [arachidonoyl ethanolamide (AEA) and palmitoyl ethanolamide (PEA)] to determine the state of endocannabinoid signaling in chronic HIV/SIV infection. Since phytocannabinoids have been reported to inhibit endocannabinoid inactivation by competing with fatty acid binding proteins [90], we hypothesized that long-term exogenous phytocannabinoid (THC) administration may increase plasma endocannabinoid levels. In agreement with this hypothesis, we detected significantly (*p* < 0.05) high concentrations of AEA (Fig. 5D), PEA (Fig. 5E), and endocannabinoid-like linoleoyl ethanolamide (LEA) (Fig. 5F) in plasma of THC/SIV compared to uninfected control RMs. Relative to controls, plasma AEA concentrations were also significantly high in VEH/SIV but at levels slightly lower than THC/SIV RMs (Fig. 5D). Interestingly, plasma concentrations of *N*-oleoyltaurine (OT) were significantly decreased in VEH/SIV compared to controls (Fig. 5G). Further, relative to VEH/SIV RMs, plasma concentrations of both LEA and OT were significantly (*p* < 0.05) higher in THC/SIV RMs (Fig. 5F, G). Metabolic pathway figures show all endocannabinoid ligands detected in the current study and those that showed significant increase (red circles) or decrease (dark blue circles) in THC/SIV (Fig. 5H) and VEH/SIV (Fig. 5I) relative to controls and in THC/SIV compared to VEH/SIV (Fig. 5J) RMs. In addition, significantly high levels of trans-urocanate were detected in plasma of THC/SIV relative to both VEH/SIV and control RMs (Additional file 1: Fig. S4A). Trans-urocanate when converted to the cis-isomer by UVB radiation serves as a chemoattractant for T regulatory cells [91], an important mechanism associated with the anti-inflammatory effects of THC.

A second important finding relevant to brain health was the presence of significantly high levels of the two neuroprotective metabolites glycerophosphorylcholine [92, 93] (Fig. 5K) and glycerophosphoinositol [94, 95] (Fig. 5L) out of seven other metabolites related to phospholipid metabolism detected (Fig. 5M) in plasma of THC/SIV compared to control RMs. Recently, we demonstrated the inhibitory effects of THC on *IDO1* mRNA and protein expression that resulted in significantly reduced plasma kynurenine, kynurenate, and quinolinate levels in the same group of THC/SIV RMs used in the current study [29]. Consistent with *IDO1* inhibition, THC/SIV RMs showed significantly lower plasma kynurenine/tryptophan (K/T) ratios (Fig. 5N), a better indicator of IDO1 functional activity than kynurenine levels. In contrast, kynurenine/tryptophan ratios were significantly high in VEH/SIV compared to control RMs. The figure for K/T ratio (Fig. 5N) was not provided by Metabolon and therefore, we calculated this value by dividing plasma concentration of kynurenine by the tryptophan concentration.

While most of the dietary tryptophan is absorbed in the small intestine, a small but significant fraction of the unabsorbed tryptophan reaches the colon where it is converted by gut bacteria into indole, skatole, and its derivatives. Since ~ 90% of dietary tryptophan is metabolized through the *IDO1* pathway, we next hypothesized that by blocking *IDO1* [29], THC may make more tryptophan available for conversion to serotonin (~ 3% of dietary tryptophan) and indole derivatives (~ 7% of dietary tryptophan). Although we did not detect a statistically significant increase in plasma levels of serotonin, concentrations of indole-3-propionate (IPA), an important tryptophan-derived indole metabolite linked to the microbiota–gut–brain [96] and microbiota–gut–heart axis [97] was significantly elevated in plasma of THC/SIV (*p* < 0.05) compared to both uninfected controls and VEH/SIV RMs (Fig. 5O). Metabolic pathway figures show all tryptophan metabolites detected in the current study and those that showed significant increase (red circles) or decrease (dark blue circles) in THC/SIV (Fig. 5P) and VEH/SIV (Fig. 5Q) relative to controls and in THC/SIV compared to VEH/SIV (Fig. 5R) RMs. Metabolites in Fig. 5P–R shown in light blue (decreased) and light pink (increased) showed a tendency to reach statistical significance (0.05 > *p* < 0.1). In addition, VEH/SIV but not THC/SIV RMs had significantly high plasma creatinine levels compared to uninfected control RMs (Fig. 5S). Unlike IPA, plasma levels of other tryptophan metabolites, namely, xanthurenate, indoleacetate, indoleacetoylcarnitine, and indoleacetylglutamine were significantly reduced in THC/SIV compared to uninfected control RMs (Additional file 1: Fig. S4B–E). Relative to controls, plasma levels of indoleacetate and indolebutyrate were significantly decreased (Additional file 1: Fig. S4C, G) while that of 3-indoxyl sulfate, *N*-formylanthranilic acid, and C-glycosyltryptophan increased significantly in VEH/SIV RMs (Additional file 1: Fig. S4F, H, I). Relative to uninfected controls, plasma 3-indoxyl sulfate levels were significantly increased in THC/SIV RMs (Additional file 1: Fig. S4F). Further, plasma levels of *N*-formylanthranilic acid and C-glycosyltryptophan were significantly reduced in THC/SIV compared to VEH/SIV RMs (Additional file 1: Fig. S4H, I). The biological significance of majority of the indole metabolites shown in Additional file 1: Fig. S4 has not been fully elucidated.

### Long-term low-dose THC increased the abundance of obligate anaerobes (*Firmicutes* and *Clostridia*) by potentially inhibiting inflammatory responses in colon of chronically SIV-infected RMs

Given that IPA is a gut bacteria-derived metabolite produced predominantly by *Clostridial* and *Peptostreptococcus* species [98], we next hypothesized that the anti-inflammatory effects of THC in the intestine [24] may reduce inflammation driven microbiota dysbiosis and better preserve and support the growth of commensal anaerobic bacteria, like *Clostridia*. The significantly high plasma levels of 3-hydroxyhippurate (3-HPA) (Fig. 5T) in THC/SIV RMs provided indirect evidence of *Clostridial* enrichment as elevated hippurate levels have been previously associated with increased abundance of operational taxonomic units within the bacterial order *Clostridiales* [99]. Accordingly, we performed shotgun metagenomic sequencing of colonic contents to obtain deeper insights into the impact of chronic THC administration on the gut microbiome.

**Fig. 6:**
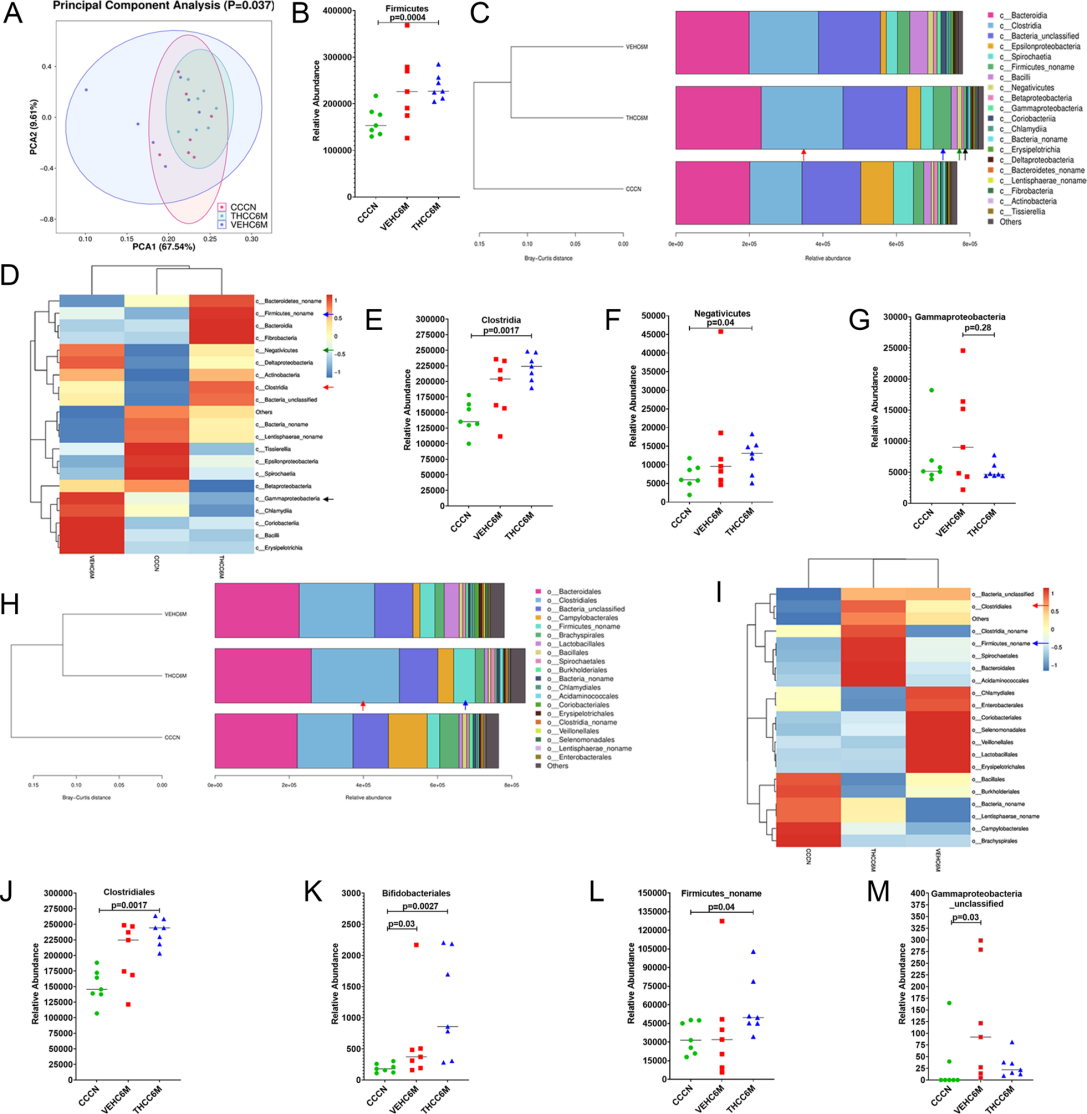
THC administration significantly increased relative abundance of *Firmicutes*, *Clostridia*, *Clostridiales* in colon of SIV-infected RMs. The Beta-diversity (PCA) (**A**), taxonomy cluster (**C**, **H**) and heat map (**D**, **I**) of top 21 colon bacterial class (**C**, **D**) and bacterial order (**H**, **I**) before and at 6 MPI in VEH or THC-treated chronically SIV-infected RMs. Dot plots show relative abundance of phylum *Firmicutes* (**B**) and class *Clostridia* (**E**), *Negativicutes* (**F**), and *Gammaproteobacteria* (**G**) in colon of VEH/SIV and THC/SIV at 6 MPI compared to uninfected control RMs. Relative abundance of order *Clostridiales* (**J**), *Bifidobacteriales* (**K**), *Firmicutes_noname* (**L**), and *Gammaproteobacteria_unclassifed* (**M**) in colon of VEH/SIV and THC/SIV relative to uninfected control RMs. *CCCN*–uninfected controls, *VEHC6M-*–VEH/SIV 6 MPI, *THCC6M-*–THC/SIV 6 MPI, MPI–months post-SIV infection. A *p*-value of < 0.05 was considered significant

It is clear from Fig. 6A that the THC/SIV group and the uninfected control group showed more convergence in beta diversity than the VEH/SIV group, suggesting THC treatment moderates SIV-induced changes in beta diversity (*p* = 0.037). Colonic contents of THC/SIV RMs at 6 MPI (THCC6M) were significantly (*p* < 0.05) enriched for phylum *Firmicutes* relative to uninfected control RMs (Fig. 6B). Notice from the Bray–Curtis distance that both SIV-infected groups (VEH/SIV and THC/SIV) were more closely related to one another in microbial composition (Fig. 6C). This pattern was observed at all six levels of bacterial classification (phylum to species). More importantly, at the level of bacterial class, *Clostridia* (red arrows in Fig. 6C–E), and *Negativicutes* (green arrows in Fig. 6C, D, F) were significantly enriched in colonic contents of THC/SIV relative to uninfected control RMs. Although statistically non-significant, levels of *Gammaproteobacteria*, a class of dysbiotic bacteria known to translocate into the systemic circulation [100] and induce systemic immune activation in HIV-infected individuals and SIV-infected RMs were comparable between THC/SIV and uninfected controls (black arrows in Fig. 6C, D, G) but markedly expanded in VEH/SIV RMs.

When looking at bacterial order, *Clostridiales* (red arrow in Fig. 6H–J), *Bifidobacteriales* (Fig. 6K), and *Firmicutes_noname* (blue arrow in Fig. 6H, I, L) showed significantly high relative abundance in THC/SIV compared to control RMs. Interestingly, unlike that observed at the level of bacterial class, the order *Gammaproteobacteria_unclassified* (Fig. 6M) was significantly increased in relative abundance in VEH/SIV but not in THC/SIV RMs relative to control RMs.

**Fig. 7:**
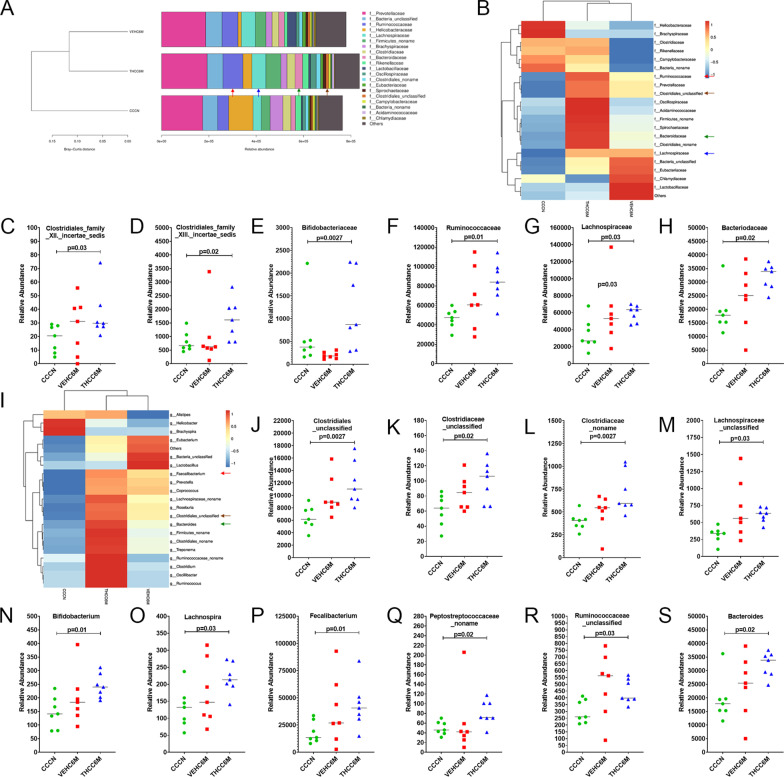
*Bifidobacteriaceae*, *Ruminococcaceae*, *Lachnospiraceae*, *Bifidobacterium*, *Lachnospira*, and *Faecalibacterium* are significantly enriched in colon of THC/SIV RMs. The taxonomy cluster (**A**) and heat map (**B**, **I**) of top 21 colon bacterial family (**A**, **B**) and genus (**I**) at 6 MPI in VEH/SIV and THC/SIV relative to uninfected control RMs. Dot plots (family) show relative abundance of *Clostridiales_Family_XII_Incertae_Sedis* (**C**), *Clostridiales_Family_XIII_Incertae_Sedis* (**D**), *Bifidobacteriaceae* (**E**), *Ruminococcaceae* (**F**), *Lachnospiraceae* (**G**) and *Bacteriodaceae* (**H**) in colon of VEH/SIV and THC/SIV relative to uninfected control RMs. Dot plots (genus) show relative abundance of *Clostridiales_unclasiffied* (**J**), *Clostridiaceae_unclassified* (**K**), *Clostridiaceae_noname* (**L**), *Lachnospiraceae_unclassified* (**M**), *Bifidobacterium* (**N**), *Lachnospira* (**O**), *Faecalibacterium* (**P**), *Peptostreptococcaceae_noname* (**Q**) *Ruminococcaceae_unclassified* (**R**), and *Bacteriodes* (**S**) in colon of VEH/SIV and THC/SIV relative to uninfected control RMs. *CCCN*–uninfected controls, *VEHC6M*–VEH/SIV 6 MPI, *THCC6M*–THC/SIV 6 MPI, MPI–months post-SIV infection. A *p*-value of < 0.05 was considered significant

At the family level, the relative abundance of *Clostridiales family XII incertae sedi*s (Fig. 7C), *Clostridiales family XIII incertae sedi*s (Fig. 7D), *Bifidobacteriaceae* (Fig. 7E),* Ruminococcaceae* (red arrow in Fig. 7A, B, F),* Lachnospiraceae* (blue arrow in Fig. 7A, B, G), and *Bacteroidaceae* (green arrow in Fig. 7A, B, H) was significantly (*p* < 0.05) higher in THC/SIV RMs compared to control RMs.

At the level of bacterial genera, *Clostridiales_unclassified* (brown arrow in Fig. 7I, J),* Clostridiaceae_unclassified* (Fig. 7K),* Clostridiaceae_noname* (Fig. 7L),* Lachnospiraceae_unclassified* (Fig. 7M), *Bifidobacterium* (Fig. 7N), *Lachnospira* (Fig. 7O), *Faecalibacterium* (red arrow in Fig. 7I, P), *Peptostreptococcaceae_noname* (Fig. 7Q), *Ruminococcaceae_unclassified* (Fig. 7R), and *Bacteroides* (green arrow in Fig. 7I, S) showed significantly (*p* < 0.05) high relative abundance in THC/SIV compared to control RMs. Overall, THC enriched the relative abundance of bacterial taxa that fall under phylum *Firmicutes*, mainly, *Clostridia*, *Ruminococcus*, *Lachnospira*, and *Faecalibacterium* in the colon of SIV-infected RMs.

### Long-term THC significantly increased the relative abundance of IPA-producing *Clostridium botulinum*, *Clostridium paraputrificum*, *Clostridium cadaveris*, and butyrate-producing *Clostridium butyricum*, *Faecalibacterium prausnitzii* and *Butyricicoccus pullicaecorum* in colon of chronically SIV-infected RMs

**Fig. 8:**
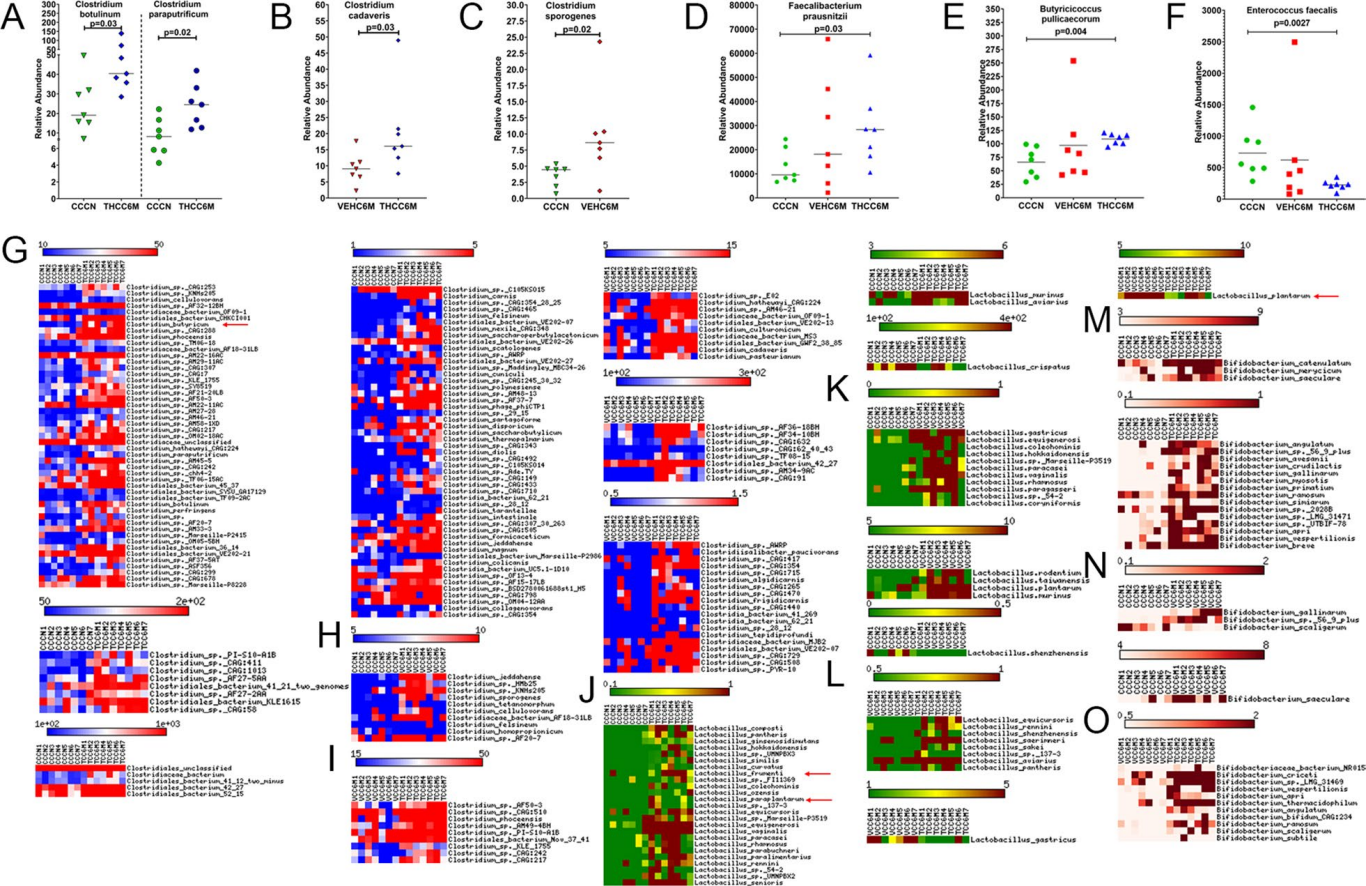
THC administration modulated relative abundance of IPA and SCFA-producing colonic species of SIV-infected RMs. Dot plots show relative abundance of Indole-3-propionate (IPA) [*Clostridium botulinum* (**A**),* Clostridium paraputrificum* (**A**), *Clostridium cadaveris* (**B**), and *Clostridium sporogenes* (**C**)] and short chain fatty acid (SCFA) [*Faecalibacterium prausnitzii* (**D**) and *Butyricicoccus pullicaecorum* (**E**)] producers, and the dysbiotic species *Enterococcus faecalis* (**F**) in colon of THC/SIV and VEH/SIV at 6 MPI relative to uninfected control RMs. Heat maps show relative abundance of commensal *Clostridia* (**G**–**I**), *Lactobacillus* (**J**–**L**) and *Bifidobacteria species* (**M**–**O**) in colon of THC/SIV (**G**, **J**, **M**), VEH/SIV (**H**, **K**, **N**) compared to uninfected control RMs and in THC/SIV (**I**, **L**, **O**) relative to VEH/SIV RMs at 6 MPI. *CCCN*–uninfected controls, *VEHC6M*–VEH/SIV 6 MPI, *THCC6M*–THC/SIV 6 MPI, MPI–months post-SIV infection. A *p*-value of < 0.05 was considered significant

While majority of the proteins ingested is degraded and absorbed in the small intestine, depending on the amount ingested, a significant amount of proteins and amino acids like tryptophan, reach the colon, where they are converted by bacteria that encode the phenyllactate dehydratase gene cluster (*fldAIBC*) or its homolog, to indole and its derivatives [98]. Consistent with high plasma IPA levels in THC/SIV RMs, we identified significantly increased relative abundance of *Clostridium botulinum* and *Clostridium paraputrificum* in colonic contents of THC/SIV relative to control RMs (Fig. 8A). When comparing THC/SIV and VEH/SIV RMs, *Clostridium cadaveris* was present at significantly higher levels in colonic contents of THC/SIV RMs (Fig. 8B). Interestingly, the relative abundance of a well-characterized IPA producer *Clostridium sporogenes* was significantly higher in VEH/SIV compared to control RMs (Fig. 8C). From the *Y*-axis values, it is clear that *Clostridium botulinum*, *Clostridium paraputrificum*, and *Clostridium cadaveris* showed greater abundance than *Clostridium sporogenes* in colonic contents (Fig. 8A–C). Three other IPA-producing *Peptostreptococcus* species were also detected, but none showed statistical significance (Additional file 1: Fig. S5). The high relative abundance of three clostridial species may partly explain the presence of significantly high plasma IPA levels in THC/SIV RMs (Fig. 5O). Further, the slight increase in IPA levels detected in plasma of VEH/SIV relative to control RMs may be attributed to the high relative abundance of *Clostridium sporogenes* (Fig. 5O). Further, and most importantly, two major anti-inflammatory butyrate (an important SCFA) producing bacteria, *Faecalibacterium prausnitzii* [101, 102] (Fig. 8D) and *Butyricicoccus pullicaecorum* [103] (Fig. 8E), showed significantly higher relative abundance in the colon of THC/SIV RMs. In contrast, *Enterococcus faecalis*, a known pathobiont enriched in stool samples of COVID-19 patients and proposed as a top predictor of severe COVID-19 disease [104] was significantly reduced in THC/SIV RMs (Fig. 8F).

In addition, we also focused on other *Clostridia*,* Lactobacillus*, *Bifidobacterium*, *Lachnospira*, and *Ruminococcu*s species because of their ability to produce indole and SCFAs that exert anti-inflammatory and neuroprotective effects on the host. In agreement with high *Clostridia* levels (class), 113 distinct *Clostridial* species showed significantly high relative abundance in THC/SIV RMs (Fig. 8G) compared to only 10 distinct species that showed differential abundance in VEH/SIV RMs (Fig. 8H) relative to control RMs. Most notably, *Clostridium butyricum* (red arrow in Fig. 8G), a commonly used probiotic species and a major butyrate producer [105] showed significantly high relative abundance in THC/SIV compared to control RMs. About 37 distinct *Clostridial* species showed high relative abundance in THC/SIV compared to VEH/SIV RMs (Fig. 8I).

Relative to control RMs, 28 distinct *Lactobacillus* species were present at significantly high levels in colonic contents of THC/SIV RMs (Fig. 8J). In VEH/SIV RMs, 16 different *Lactobacillus* species showed statistical significance compared to control RMs (Fig. 8K). When comparing the VEH and THC groups, 9 *Lactobacillus* species showed statistical significance. Out of these, 8 were significantly up in the THC/SIV group and *Lactobacillus plantarum*, an important probiotic bacterium [106] was higher in the VEH/SIV group (Fig. 8L). *Lactobacillus frumenti* [107] (red arrow in Fig. 8J) has been shown to exert beneficial effects in the GIT by improving epithelial barrier function when used as a probiotic. Similarly, 18 (Fig. 8M) and 4 (Fig. 8N) *Bifidobacterium* species showed significantly high abundance in THC/SIV and VEH/SIV, respectively, relative to control RMs. When comparing THC/SIV and VEH/SIV RMs, 11 *Bifidobacterium* species showed significantly high abundance in colonic contents of THC/SIV RMs (Fig. 8O). Additionally, we also detected a higher number of *Ruminococcus* and *Lachnospira* species that showed significantly higher relative abundance in THC/SIV than VEH/SIV compared to control RMs (Additional file 1: Fig. S6). These findings identify a novel cannabinoid-based strategy to reduce dysbiosis in HIV/SIV [108, 109] and most strikingly, the anti-dysbiotic potential of THC even in the absence of cART.

LEfSe analysis was performed to identify the ranking of abundant bacterial modules. The cladogram (Additional file 1: Fig. S7A–C) shows the taxa that differed in abundance among the three treatment group comparisons. Similarly, the plot from LEfSe analysis shows LDA scores of microbial taxa with significant differences in the VEH/SIV vs controls, THC/SIV vs controls and VEH/SIV vs THC/SIV comparisons are shown in Additional file 1: Fig. S7D–F, respectively. While we detected Class *Clostridia*, order *Clostridiale*s, and phylum *Actinobacteria* in both VEH/SIV and THC/SIV relative to the uninfected control group (Additional file 1: Fig. S7A, B, D, E), phylum *Firmicutes*, Family *Ruminococcaceae* and *Lachnospiraceae*, genus *Faecalibacterium* and species *Faecalibacterium prausnitzii*, showed significant abundance exclusively in THC/SIV group compared to the uninfected control group (Additional file 1: Fig. S7B, E). When comparing THC/SIV to the VEH/SIV group, class *Bacilli* was detected exclusively in the VEH/SIV group, while family *Clostridiales_noname*, genus *Clostridiales_noname*, and *Ruminococcus* and species *Firmicutes_bacterium_CAG-95* showed significant relative abundance solely in colonic contents of THC/SIV RMs (Additional file 1: Fig. S7C, F).

## Discussion

Although the advent of cART has transformed the HIV epidemic into a manageable chronic disease, PLWH continue to be adversely impacted by non-AIDS-associated comorbidities, co-infections, and complications. HAND, experienced by about 40–50% of PLWH, continues to pose a significant clinical problem as currently available therapeutic interventions, including cART, does not improve or prevent HAND symptoms [1–4]. While the exact pathological mechanisms contributing to HAND remain unclear, available evidence points to the consequences of neuronal injury by viral proteins, chronic release of toxic metabolites, chemokines, and proinflammatory cytokines by activated microglia, macrophages, and astrocytes [4]. The massive infection of the GIT and brain by HIV/SIV during acute infection combined with reduced penetration of cART drugs into deeper areas of both organs facilitates low level viral replication leading to chronic persistent inflammation, dysbiosis, and eventually dysfunction of the MGBA. We recently reported the potential of phytocannabinoids to successfully inhibit intestinal and gingival inflammation and reduce salivary dysbiosis in chronic SIV-infected RMs [29]. Since cannabinoids can cross the BBB due to its high lipophilicity and has shown beneficial effects in animal models of neurodegenerative (AD, PD, HD) and inflammatory diseases (MS) [110], we hypothesized that cannabinoids may exert similar effects in HIV infection (HAND) by positively modulating the MGBA.

To determine the effect of THC on the brain during chronic SIV infection, we undertook a systems biology approach by profiling genome wide changes in transcriptome and microRNA expression in whole BG of chronically SIV-infected RMs. We selected the basal ganglia because this brain region controls motor activity [111] and is severely impacted by HIV/SIV due to its susceptibility to high levels of viral replication [112–114]. In line with the host response to active viral replication, a gene expression signature dominated by chemokines (*CCL2*, *CXCL10*), neuroimmune activation (*NLRC5*), NFκB activation (*RNF31*), and more importantly, type-I IFN response (*ISG15*, *ISG20*, *HERC5*, *IFIT1*, *IRF1*, *IRF7*), and type I IFN stimulated antiviral signaling (*ZC3HAV1*, *TMRM173*, *DDX58*, *MX1*, *BST2*, *IFITM3*, *IFITM1*), was significantly upregulated in BG of VEH/SIV RMs. *CCL2* and *CXCL10* play proinflammatory roles in viral infections by stimulating the activation and migration of immune cells to the sites of viral replication including the brain [43]. Moreover, activation of the *CXCL10/CXCR3* axis contributes to microglial activation leading to persistent neuroinflammation [26, 43]. Similarly, high *CCL2* levels confirmed in plasma and CSF of cART-treated and naïve HIV individuals have been associated with an increased percentage of CD14/CD16^++^ monocytes and neuroimmune activation [44]. *IRF7* is a transcription factor that not only induces and amplifies type-I IFN responses but also regulates adaptive immune responses by inducing the expression of *PSMB9* directly, or indirectly through *IRF1* induction [115]. Interestingly, all three genes (*IRF7*, *PSMB9*, and *IRF1*) were significantly upregulated in BG of VEH/SIV RMs suggesting active type-I IFN signaling mediated by this sequential activation [115]. Despite not reaching statistical significance, expression of *CD68* (microglial activation) (*p* = 0.08) and *OSMR* (astrocyte activation) (*p* = 0.05) was markedly upregulated in BG of VEH/SIV RMs suggesting activation of both microglia and astrocytes. Consistent with our findings, in PLWH on cART, CSF levels of IFNα correlated positively with milder forms of HAND and levels of neurofilament light chain, a marker of neuronal injury [45].

Interestingly, ~ 1.5-fold fewer genes were upregulated in the BG of THC/SIV RMs compared to uninfected RMs. Although we detected enhanced expression of several IFN and antiviral genes, the read counts and fold change for most of these were significantly lower in BG of THC/SIV compared to VEH/SIV RMs. *TRIM22* was the only IFN-induced antiviral gene that showed differential upregulation in BG of THC/SIV RMs. More importantly, the upregulation of several prominent genes associated with antiviral response (*n* = 12), regulation of the IFN (*n* = 4), and inflammatory response (*n* = 6) detected in BG of VEH/SIV RMs was effectively suppressed by cannabinoid treatment even in the absence of cART. In contrast, THC significantly upregulated the expression of *WFS1* (anti-ER stress protein), *TNFSD10* (an inducer of apoptosis of HIV-infected macrophages), and *NOS3* (also known as endothelial nitric oxide synthase, that inhibits neuroinflammation). While the mechanisms by which HIV causes HAND is unclear, viral establishment in the brain before cART initiation is thought to trigger persistent inflammatory responses that are not resolved by cART. Beyond their antiviral activity, persistent type-I IFNs are thought to be key drivers of the self-perpetuating neuroinflammatory responses that contribute to neuropsychiatric and cognitive dysfunction in AD, PD [47, 116], traumatic brain injury [117, 118], and PLWH on suppressive cART [45]. Despite its success in the periphery [119], blocking type-I IFN signaling in the brain using currently available anti-IFNAR neutralizing antibodies can be challenging because the BBB prevents macromolecules like antibodies from entering the brain through normal paracellular or diffusion routes*.* In this context, the finding that cannabinoids can successfully cross the BBB [120] and reduce type-I IFN responses is a major highlight of our study and identifies its utility as a viable approach to alleviate neuroinflammation in not only HIV/SIV but also other neurodegenerative diseases like AD, PD, HD, etc., where persistent type-I IFN responses have been shown to drive neuropathology [116]. Although THC-mediated reduction in IFN stimulated anti-viral gene expression resulted in high BG viral loads in a subset of RMs, it is important to note that chronic immune activation and inflammation, not viral replication are the major determinants of HIV/SIV disease progression. Therefore, while the anti-inflammatory effects of low-dose cannabinoids are clearly evident even in the absence of cART, these beneficial effects may be maximized when used in conjunction with cART so that both viral replication and chronic inflammation can be simultaneously targeted to reduce the occurrence of inflammation driven comorbidities and improve the overall quality of life of PLWH.

While several genes (*TMEFF2* [50, 51], *GLRA2* [57], *FEZF2* [53], *CNIH3* [59], *SNCA* [60], *CALB1* [62, 121], *RAPGEF4* [55, 56]) associated with a multitude of diverse functions in neurons were downregulated in BG of VEH/SIV RMs, chronic THC administration, in contrast, significantly reduced the expression of genes regulating serine/glutamine transport (*SLC38A2*), glutamate release induced neuronal excitotoxicity (*SLC7A11*), apoptosis (*FEM1B*), and neuroinflammation via activation of NFκB and MAPK pathways (*HDAC9*). Glutamate modulates normal neural signaling but contributes to excitotoxicity in pathological conditions like HAND, where dysregulated glutamate metabolism in the CNS results in elevated extracellular glutamate and dysregulated glutamatergic neurotransmission. *SLC38A2* is a Na+-coupled transporter for neutral amino acids, like glutamine, expressed predominantly in neurons. Following *SLC38A2-*mediated uptake into the presynaptic terminals, glutamine is metabolized by the phosphate-activated mitochondrial enzyme glutaminase to glutamate, which is then released into the synaptic cleft during neuronal activation, thereby increasing extracellular glutamate levels. Expression of *SLC7A11*, a cystine/glutamate transporter is significantly increased in malignant glioma [74] and during brain ischemia [75] contributing to increased extracellular glutamate levels resulting in overstimulation of NMDA receptors, seizures and neuronal death. As seizures are common in glioma patients, sulfasalazine, an FDA-approved potent but short-term inhibitor of *SLC7A11* commonly prescribed to inflammatory bowel disease patients to alleviate colonic inflammation reduced glutamate excitotoxicity and seizures in a pilot study involving nine glioma patients [74]. Even though cannabinoids have been proposed to reduce excitotoxicity [122] the exact mechanisms remain poorly understood. Therefore, the downregulation of *SLC38A2* and *SLC7A11*, after cannabinoid administration is a clinically relevant finding as it identifies an alternative yet feasible approach and a potential mechanism by which cannabinoids may lower extracellular glutamate levels to reduce excitotoxicity. Similarly, *HDAC9* has been shown to exacerbate brain ischemic injury by activation of NFκB and MAPK signaling pathways [71] and its silencing by miR-20a resulted in neuroprotection [72]. Our data suggest that in HIV/SIV infection, cannabinoids may exert neuroprotective effects by reducing glutamine/glutamate uptake/transport, excitotoxicity and neuroinflammation partly through downregulation of *SLC38A2*, *SLC7A11*, and *HDAC9*. Overall, THC administration preserved expression of genes associated with neuronal survival, proliferation, and differentiation, while reducing the expression of those associated with glutamate cellular transport and excitotoxicity in chronic HIV/SIV infection.

Owing to the role of the unfolded protein response and its consequent induction of ER stress in neuronal dysfunction [65], we further characterized *WFS1*, a negative regulator of a feedback loop of the ER stress signaling network [64, 82] that showed significantly increased mRNA and protein expression in BG neurons of THC/SIV RMs. *WFS1* is a negative regulator of ER stress [64], a key event involved in the onset of neurodegenerative diseases [123, 124], by preventing ATF6 activation thereby blocking the expression of proteins (CHOP, ATF4, BIP, and sXBP1) that promote cellular apoptosis. Downregulation of *WFS1* in neurons inhibited mitochondrial fusion, altered mitochondrial trafficking, and augmented mitophagy thereby, delaying neuronal development [125]. More recently, *WFS1* was shown to facilitate Ca^2+^ transfer between the ER and mitochondria by forming a complex with neuronal calcium sensor 1 (*NCS1*) and inositol 1,4,5-trisphosphate receptor [126]. In *WFS1* mutant fibroblasts, ER-mitochondria interactions, and Ca^2+^ exchange was reduced leading to cell death [126]. The reduced WFS1 protein expression in BG neurons of VEH/SIV RMs may partially explain the mitochondrial abnormalities reported [127] to drive neuronal dysfunction in HIV-infected patients. Collectively, our findings suggest that by enhancing WFS1 protein expression, THC may protect neurons against ER stress and mitochondrial damage in not only HIV infection but also other inflammation-associated neurodegenerative diseases.

Apart from *WFS1*, chronic THC also significantly enhanced mRNA and protein expression of *CRYM* that functions both as a cytoplasmic thyroid hormone binding protein and ketimine reductase (*KR*). The KR activity is potently inhibited by thyroid hormones, which implies the existence of a reciprocal relationship between enzyme catalysis and thyroid hormone bioavailability [80]. A well-studied function of *CRYM*/*KR* is its role as a Δ1-piperideine-2-carboxylate (P2C) reductase in the pipecolate pathway of lysine metabolism that occurs predominantly in peroxisomes in adult brain [80]. Interestingly, *CRYM* mRNA [128, 129] and protein [130] levels were downregulated in the prefrontal cortex of schizophrenic rats and humans. Similarly, *CRYM* gene expression was significantly reduced in striatal neurons of HD patients and its overexpression using a lentiviral based strategy successfully protected neurons against mutant Huntington protein induced toxicity [131]. Interestingly, in mouse models of schizophrenia, an 8-week treatment with the anti-depressant drug Amitriptyline resulted in massive upregulation of *CRYM* gene expression (~ 24-fold) [129] indirectly suggesting that levels of pipecolate with potential neuroactive functions may be decreased in the brains of schizophrenic mice and humans. Like amitriptyline, long-term, low-dose significantly increased *CRYM* mRNA and protein expression in the brain suggesting a neuroprotective role for pipecolate. Similar to WFS1, CRYM protein also localized predominantly exclusively to neurons. Given that HIV-induced neuroinflammatory signaling can induce ER stress and vice versa, our findings suggest that cannabinoids may concurrently reduce both ER stress and neuroimmune activation through induction of WFS1 protein. Further, by enhancing *CRYM* expression, cannabinoids may positively regulate pipecolate levels in the brain during HIV/SIV infection. Overall, these findings of a previously unanticipated association between reduced WFS1 and CRYM protein expression and HIV/SIV-induced neuroimmune activation provide novel insights into the pathogenesis of HAND and the translational value of cannabinoids in attenuating HIV/SIV-mediated neuroimmune activation, ER and oxidative stress.

To gain insight into the epigenetic regulation of differential gene expression, we profiled miRNA expression in BG of a subset of VEH/SIV and THC/SIV RMs and detected significantly elevated expression of miR-155 and miR-142-3p in BG of VEH/SIV RMs. Interestingly, dysregulated miR-155 and miR-142-3p has been previously reported in both HIV and non-HIV induced encephalitic conditions like MS [85–87]. In SIV-infected RMs, significantly increased miR-142-3p expression was detected in neurons and myeloid cells in the dentate gyrus and hippocampus and follow-up studies confirmed its ability to directly target and downregulate the expression of anti-oxidant protein SIRT1 [86]. Bioinformatics analysis identified *WFS1* as a predicted target of miR-142-3p. Using luciferase reporter assays and controlled in vitro overexpression experiments in HCN2 primary cortical neurons, we confirmed the ability of miR-142-3p to physically bind the 3′ UTR of *WFS1* mRNA and significantly reduce its protein expression, suggesting the potential for epigenetic regulation in HIV/SIV infection. Taken together, the emerging theme from our findings and those reported by Chaudhuri et al. [86] is that dysregulated miR-142-3p may contribute to HIV/SIV neuropathogenesis by directly targeting genes that protect against oxidative (*SIRT1*) and ER (*WFS1*) stress. From a clinical standpoint, while previous studies have linked dysregulated miR-142-3p to enhanced neuroinflammatory responses [86, 87], we have gone one step further to demonstrate for the first time that long-term, low-dose cannabinoids can override the inhibitory effects of miR-142-3p on WFS1 protein expression in vitro and potentially in vivo via a CB1R-mediated mechanism even in the absence of cART.

The significantly increased *CRYM* gene and protein expression was intriguing and prompted us to determine if this resulted in elevated pipecolate levels. Accordingly, we performed metabolomic profiling and detected significantly high plasma pipecolate levels only in THC/SIV RMs. The pipecolate pathway plays important roles in neuronal development and function and both in vitro and in vivo evidence suggests that pipecolate has anti-oxidative properties and its high plasma levels may be to ensure the increased availability of anti-oxidant agents at sites of inflammation. Apart from pipecolate, we also detected elevated levels of entero- and neuroprotective endocannabinoids (AEA, PEA, LEA, and OT) [88, 89] in plasma of THC/SIV RMs. Another very important finding was the significantly increased levels of two entero-, cardio-, and neuroprotective metabolites, IPA and glycerophosphorylcholine [92], in plasma of THC/SIV compared to control RMs. Further, THC/SIV RMs also showed significantly higher plasma IPA levels compared to VEH/SIV RMs. IPA, a tryptophan-derived bacterial metabolite, exerts protective effects at the cellular and tissue level, by reducing inflammation, lipid peroxidation, and generation of free radicals, and its plasma levels represent a surrogate marker of microbiome diversity [132]. IPA can strengthen the intestinal mucus barrier by increasing the production of mucins (MUC2 and MUC4) and goblet cell secretion products (TFF3 and RELMβ) through activation of pregnane-x-receptors [132]. Because of its superior ability to neutralize free radicals (anti-oxidant) than melatonin and inhibit amyloid beta fibril formation, IPA (Oxigon™) is prescribed for the symptomatic treatment of AD [133]. More importantly, unlike other anti-oxidants like vitamin C and E, and melatonin, IPA does not produce pro-oxidant intermediates while neutralizing free radicals, which can counter the potential beneficial effects of the parent molecules [133]. Apart from its anti-oxidant properties, IPA can inhibit the growth of *Mycobacterium tuberculosis* (Mtb) [134] and can stimulate the secretion of incretin (GLP-1) to improve insulin secretion and protect against type 2 diabetes progression [135]. Similar to HD patients [136], PLWH also have significantly reduced plasma concentrations of IPA [137], which might partly explain the reduced ability to protect against reactive oxygen species formation in the brain and subsequent neuronal injury. Interestingly, significantly high plasma IPA and serotonin levels were also detected in THC/SIV RMs on cART (unpublished information). A major limitation of our study is the lack of parallel metabolomic (endocannabinoid, IPA) data in the CSF, which we are addressing in our ongoing studies. Taken together, our data suggest that cannabinoids can potentially be beneficial to PLWH in lowering their risk for developing inflammation driven comorbidities such as HAND, cardiovascular disease [138–140], type 2 diabetes, and potentially coinfection with *Mtb* through enhancement of IPA production even in the absence of cART. Overall, cannabinoid mediated targeting of IPA represents an alternative yet promising approach to link intestinal health with the nervous system, and by extension the cardiovascular system.

Although numerous bacterial species that carry the phenyllactate dehydratase gene cluster or its homolog are able to metabolize tryptophan in vitro [98], studies linking the relative abundances of bacterial species with circulating plasma concentrations of tryptophan metabolites like IPA, under healthy and disease states are lacking. Such studies are needed to identify the main tryptophan metabolite producers in the intestine so that interventions to modulate their relative abundances can be developed. To directly address this question, we performed shotgun metagenomic sequencing and identified increased relative abundance of *C. botulinum*, *C. paraputrificum*, and *C. cadaveris* in the colon of THC/SIV RMs relative to uninfected control and VEH/SIV RMs, respectively. Our data also suggest that *C. sporogenes* may not be the predominant IPA-producing species as reported in the literature given their levels were not statistically significant in THC/SIV RMs (Additional file 1: Fig. S5) despite significantly high plasma IPA levels. Apart from IPA, long-term THC also significantly increased the relative abundances of three neuroprotective SCFA (butyrate) producing bacteria *C. butyricum* [105], *F. prausnitzii* [101, 102], and *B. pullicaecorum* [103]. In addition, THC/SIV macaques had significantly higher relative abundance of *Firmicutes* (phylum), *Clostridia* (class), *Clostridiales* (order), *Bifidobacteriales* (order), *Bifidobacteriaceae*, *Ruminococcaceae*, *Lachnospiraceae* (family), *Faecalibacterium*, *Bifidobacterium*, *Peptostreptococcaceae*, *Lachnospira* (genus) and reduced relative abundance of class *Gammaproteobacteria* and order *Gammaproteobacteria_unclassified*. In addition, THC selectively elevated the relative abundance of 28 *Lactobacilli* species including the widely used probiotic species, *L. frumenti* and *L. paraplantarum*, and 18 Bifidobacterial species. Finally, THC also decreased the relative abundance of *E. faecalis*, a dysbiotic bacterial species shown to be present at high levels in the feces of COVID-19 patients [104]. To our knowledge, these findings provide novel and deeper insights into the microbiome modulating properties of long-term, low-dose THC that, with additional studies in combination with other minor cannabinoids, may be clinically applied to increase the production of neuro- and cardioprotective indole metabolites, like IPA in HIV/SIV infection.

## Conclusions

**Fig. 9:**
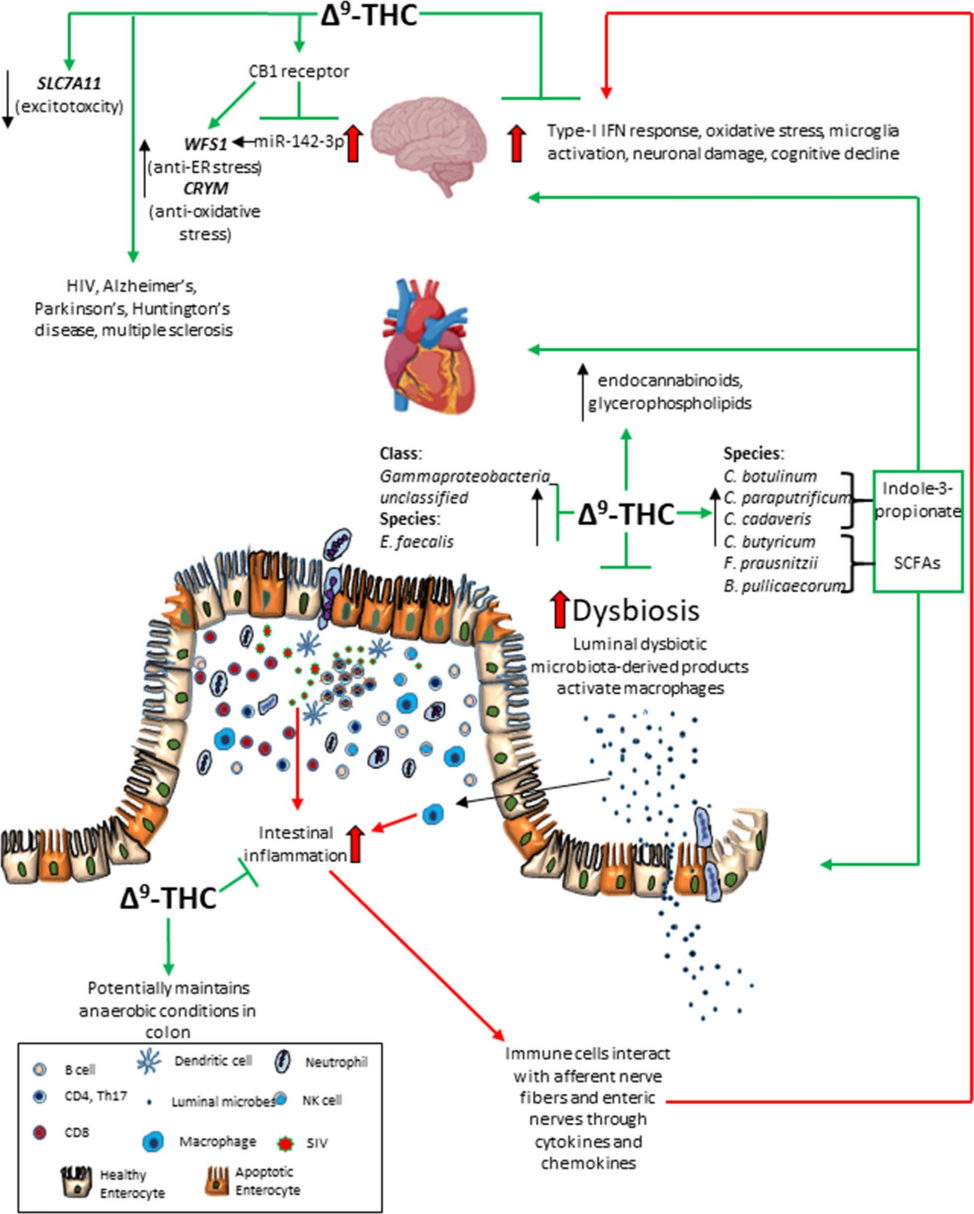
Proposed mechanisms by which long-term, low-dose THC modulates the microbiota–gut–brain axis in HIV/SIV infection. Massive infection and persistence of HIV/SIV in the gastrointestinal tract early in the disease course leads to significant structural and functional damage that is not reversed by anti-retroviral therapy. This leads to persistent GIT inflammation, dysbiosis and disruption of the intestinal epithelial barrier and microbial translocation. Activated immune cells in the GIT can interact with the afferent nerve fibers and enteric nerves through the release of cytokines and chemokines. Both cytokines and translocated microbial products (LPS) can systemically reach the brain and activate microglia resulting in increased type-I interferon responses, decreased *WFS1* and *CRYM* gene expression, and increase in miR-142-3p expression, potentially leading to endoplasmic reticulum and oxidative stress, all of which can lead to neuronal damage, and cognitive decline. Because of its high lipophilicity, cannabinoids (Δ^9^-THC) can efficiently cross the blood–brain barrier and attenuate type-I interferon responses, excitotoxicity (decreasing *SLC7A11* expression), oxidative stress through increased expression of *CRYM*, and endoplasmic reticulum stress by enhancing WFS1 protein expression through counteracting the transcriptional silencing capabilities of miR-142-3p in a CB1R-dependent fashion. Lastly, by inhibiting intestinal inflammation, THC can help maintain anaerobic conditions in the colon, which modulates the microbiota composition, resulting in a positive shift in the microbial profile, from pathobionts like *Gammaproteobacteria_unclassified* (class) and *Enterococcus faecalis*, to microbes that produce short chain fatty acids (butyrate) (*Clostridium butyricum*, *Faecalibacterium prausnitzii* and *Butyricicoccus pullicaecorum*), and more importantly, indole-3-propionate (*Clostridium botulinum*, *Clostridium paraputrificum*, *Clostridium cadaveris*). In this way, low-dose cannabinoids can reduce neuroinflammation, dysbiosis and potentially slow down cognitive decline in not only HIV but also other neurodegenerative diseases like Alzheimer’s, Parkinson’s, Huntington’s disease, multiple sclerosis, etc. The images representing brain and heart were obtained from BioRender’s list of ready-to-use images

Overall, our findings provide several lines of evidence highlighting the translational potential of cannabinoids to positively modulate the MGBA in HIV/SIV infection and potentially other neurodegenerative diseases (Fig. 9). First, owing to its high lipophilic nature, phytocannabinoids like THC can efficiently cross the BBB and block type I IFN responses, reduce oxidative/ER stress in neurons (CB1R mediated), and potentially inhibit microglial activation (likely CB2R mediated), and in doing so may prevent cognitive decline in not only HIV but also other neurodegenerative diseases like AD, PD and, HD. Second, THC can exert beneficial local and systemic effects indirectly by elevating endocannabinoid, endocannabinoid-like, glycerophosphorylcholine, and glycerophosphoinositol levels. Third, in addition to the current therapeutic approaches under consideration that include modifying or reseeding the existing gut microbiota using prebiotics, probiotics, or fecal microbial transplantation [141, 142], our findings identify a novel approach using low-dose cannabinoids, wherein inhibiting intestinal inflammation, that we previously showed in the same cohort of SIV-infected RMs [24], maintains a state of physiologic hypoxia in the colonic epithelium [143], thereby allowing the predominant obligate anaerobic commensal bacteria (99%) like *Clostridia*, *Bifidobacteria*, *Faecalibacterium*, and *Butyricicoccus* species to flourish while preventing the untoward expansion of opportunistic pathogenic bacteria like *Gammaproteobacteria*. Specifically, in combination with a high fiber diet, low-dose cannabinoids may promote the growth of commensal bacteria like *C. botulinum*,* C. paraputrificum*, *C. cadaveris*, *F. prausnitzii* and* B. pullicaecorum* that can act on tryptophan and other indigestible polymers and fermentable sugars to synthesize beneficial metabolites, like IPA and SCFAs (butyrate), which may protect against chronic inflammation driven intestinal, neurological, cardiovascular, and metabolic comorbidities in PLWH. It is very important that the well-controlled low-dose THC used in the current study should not be considered the same as marijuana smoking as smoked cannabis, in addition to numerous other phytocannabinoids and terpenes, also contains harmful smoke combustion products that may cause significant lung and cardiovascular injury. While the beneficial effects are encouraging, future NHP studies utilizing a combination of low-dose THC and other minor cannabinoids (cannabidiol [144], cannabichromene) to reduce the required dose of THC (eliminate any residual psychotropic effects) are needed. This could be an alternative strategy for the management of the microbiota to stimulate a higher production of neuroprotective tryptophan metabolites to promote brain health in PLWH. If successful, this effective and inexpensive approach may offer a new therapeutic paradigm that could add productive years of life to PLWH, 40–50% of who would otherwise develop HAND.

## Supplementary Information

**Additional file 1: Figure S1.** Basal ganglia viral loads in chronically SIV-infected rhesus macaques administered vehicle (VEH/SIV) or delta-9-tetrahydrocannabinol (THC/SIV). **Figure S2.** QQ plots showing normal distribution of WFS1 (A) and CRYM (B) confocal image quantitation data. **Figure S3.** Cannabinoid receptor 1 (CB1R) (**A**) and 2 (CB2R) (**B**) is abundantly expressed in in vitro cultured HCN2 neuronal cells. Both panels involve dual labels with CB1R (**A**) and CB2R (**B**) in red and DAPI for nuclear staining in blue. **Figure S4.** Concentrations of trans-urocanate (**A**), xanthurenate (**B**) and other tryptophan metabolites (**C**–**I**) that showed statistically significant increase or decrease in plasma of uninfected control RMs and chronically SIV-infected RMs administered vehicle or delta-9-tetrahydrocannabinol. **Figure S5.** Relative abundance of seven phenyllactate dehydratase gene cluster or its homolog encoding *Clostridia* and *Peptostreptococcus* species that were detected in colonic contents of THC/SIV (**A**) and VEH/SIV (**B**) relative to uninfected control RMs and in THC/SIV relative to VEH/SIV RMs (**C**). (*) indicates *p* < 0.05. **Figure S6.** Relative abundance of statistically significant *Ruminococcus* and *Lachnospira* species that were detected in colonic contents of THC/SIV (**A**, **D**) and VEH/SIV (**B**, **E**) relative to uninfected control RMs, and in THC/SIV relative to VEH/SIV RMs (**C**, **F**). **Figure S7.** Linear discriminant analysis effect size (LEfSe) analysis was used to generate the cladograms (**A**–**C**) and LDA scores (**D**–**F**) to show taxa differences that were detected in colonic contents of VEH/SIV (**A**, **D**) and THC/SIV (**B**, **E**) relative to uninfected control RMs, and THC/SIV relative to VEH/SIV RMs (**C**, **F**).

**Additional file 2: Table S1.** List of key immune response genes found to be upregulated exclusively in BG of VEH/SIV compared to uninfected control RMs. **Table S2.** List of downregulated genes in BG of VEH/SIV compared to uninfected control RMs. **Table S3.** List of upregulated genes in BG of THC/SIV compared to uninfected control RMs. **Table S4.** List of downregulated genes in BG of THC/SIV compared to control RMs. **Table S5.** IFN-induced and immune response genes in BG of VEH/SIV and THC/SIV relative to uninfected RMs. **Table S6.** **A** List of upregulated and unique genes in basal ganglia of THC/SIV compared to VEH/SIV RMs. **B** List of downregulated and unique genes in basal ganglia of THC/SIV compared to VEH/SIV RMs.

**Additional file 3: Table S7.** Additional Methods.

## Data Availability

The RNA-seq (Novogene), shotgun metagenomic sequencing (LC Sciences) and OpenArray microRNA raw data files have been deposited in GEO. Links to access the datasets are provided in the methods section.

## Abbreviations

3-HPA: 3-Hydroxyhippurate
AD: Alzheimer’s disease
AEA: Arachidonoyl ethanolamide
BBB: Blood brain barrier
BG: Basal ganglia
cART: Combined anti-retroviral therapy
CB1R: Cannabinoid receptor-1
CB2R: Cannabinoid receptor-2
CDS: Coding regions
CNS: Central nervous system
CRYM: Ketimine reductase/Crystallin mu
CSF: Cerebrospinal fluid
DE: Differentially expressed
GI: Gastrointestinal
GIT: Gastrointestinal tract
GO: Gene ontology
HAND: HIV-associated neurocognitive disorder
HD: Huntington’s disease
IDO1: Indoleamine 2-3 dioxygenase-1
IFN: Interferon
IPA: Indole-3-propionate
K/T: Kynurenine/tryptophan ratio
KR: Ketimine reductase
LEA: Linoleoyl ethanolamide
MGBA: Microbiota–gut–brain axis
MPI: Months post-infection
MS: Multiple sclerosis
*Mtb*: *Mycobacterium tuberculosis*
OT: *N*-Oleoyltaurine
P2C: Δ1-Piperideine-2-carboxylate
PD: Parkinson’s disease
PEA: Palmitoyl ethanolamide
PLWH: People living with HIV
RMs: Rhesus macaques
SCFAs: Short chain fatty acids
THC: Delta-9-tetrahydrocannabinol
THC/SIV: THC-treated SIV-infected
THCC6M: THC/SIV RMs at 6 MPI
VEH: Vehicle
VEH/SIV: Vehicle-treated SIV-infected
VEHC6M: VEH/SIV RMs at 6 MPI
WFS1: Wolfram syndrome-1
