# Supplementary material for: Cannabinoids modulate the microbiota–gut–brain axis in HIV/SIV infection by reducing neuroinflammation and dysbiosis while concurrently elevating endocannabinoid and indole-3-propionate levels
Source: J Neuroinflammation. 2023 Mar 8;20:62. doi: 10.1186/s12974-023-02729-6 (PMC9993397; doi:10.1186/s12974-023-02729-6)
Supplement: Supplementary file 1 — Additional file 1: Figure S1. Basal ganglia viral loads in chronically SIV-infected rhesus macaques administered vehicle (VEH/SIV) or delta-9-tetrahydrocannabinol (THC/SIV). Figure S2. QQ plots showing normal distribution of WFS1 (A) and CRYM (B) confocal image quantitation data. Figure S3. Cannabinoid receptor 1 (CB1R) (A) and 2 (CB2R) (B) is abundantly expressed in in vitro cultured HCN2 neuronal cells. Both panels involve dual labels with CB1R (A) and CB2R (B) in red and DAPI for nuclear staining in blue. Figure S4. Concentrations of trans-urocanate (A), xanthurenate (B) and other tryptophan metabolites (C–I) that showed statistically significant increase or decrease in plasma of uninfected control RMs and chronically SIV-infected RMs administered vehicle or delta-9-tetrahydrocannabinol. Figure S5. Relative abundance of seven phenyllactate dehydratase gene cluster or its homolog encoding Clostridia and Peptostreptococcus species that were detected in colonic contents of THC/SIV (A) and VEH/SIV (B) relative to uninfected control RMs and in THC/SIV relative to VEH/SIV RMs (C). (*) indicates p < 0.05. Figure S6. Relative abundance of statistically significant Ruminococcus and Lachnospira species that were detected in colonic contents of THC/SIV (A, D) and VEH/SIV (B, E) relative to uninfected control RMs, and in THC/SIV relative to VEH/SIV RMs (C, F). Figure S7. Linear discriminant analysis effect size (LEfSe) analysis was used to generate the cladograms (A–C) and LDA scores (D–F) to show taxa differences that were detected in colonic contents of VEH/SIV (A, D) and THC/SIV (B, E) relative to uninfected control RMs, and THC/SIV relative to VEH/SIV RMs (C, F). [file 12974_2023_2729_MOESM1_ESM.docx]

**Supplementary Materials for**

**Cannabinoids modulate the microbiota-gut-brain axis in HIV/SIV infection through reducing neuroinflammation and dysbiosis while concurrently elevating endocannabinoid and indole-3-propionate levels**

Marina McDew-White, Eunhee Lee, Lakmini S. Premadasa, Xavier Alvarez, Chioma M. Okeoma, Mahesh Mohan

*Corresponding author. Email: [mmohan@txbiomed.org](mailto:mmohan@txbiomed.org)

**This file includes:**

Supplementary Figures 1 to 7

**Supplemental Figures**

**Additional file 1: Fig. S1** Basal ganglia (A) and colon (B) viral loads in chronically SIV-infected rhesus macaques administered vehicle (VEH/SIV) or delta-9-tetrahydrocannabinol (THC/SIV).

**A**

**B**


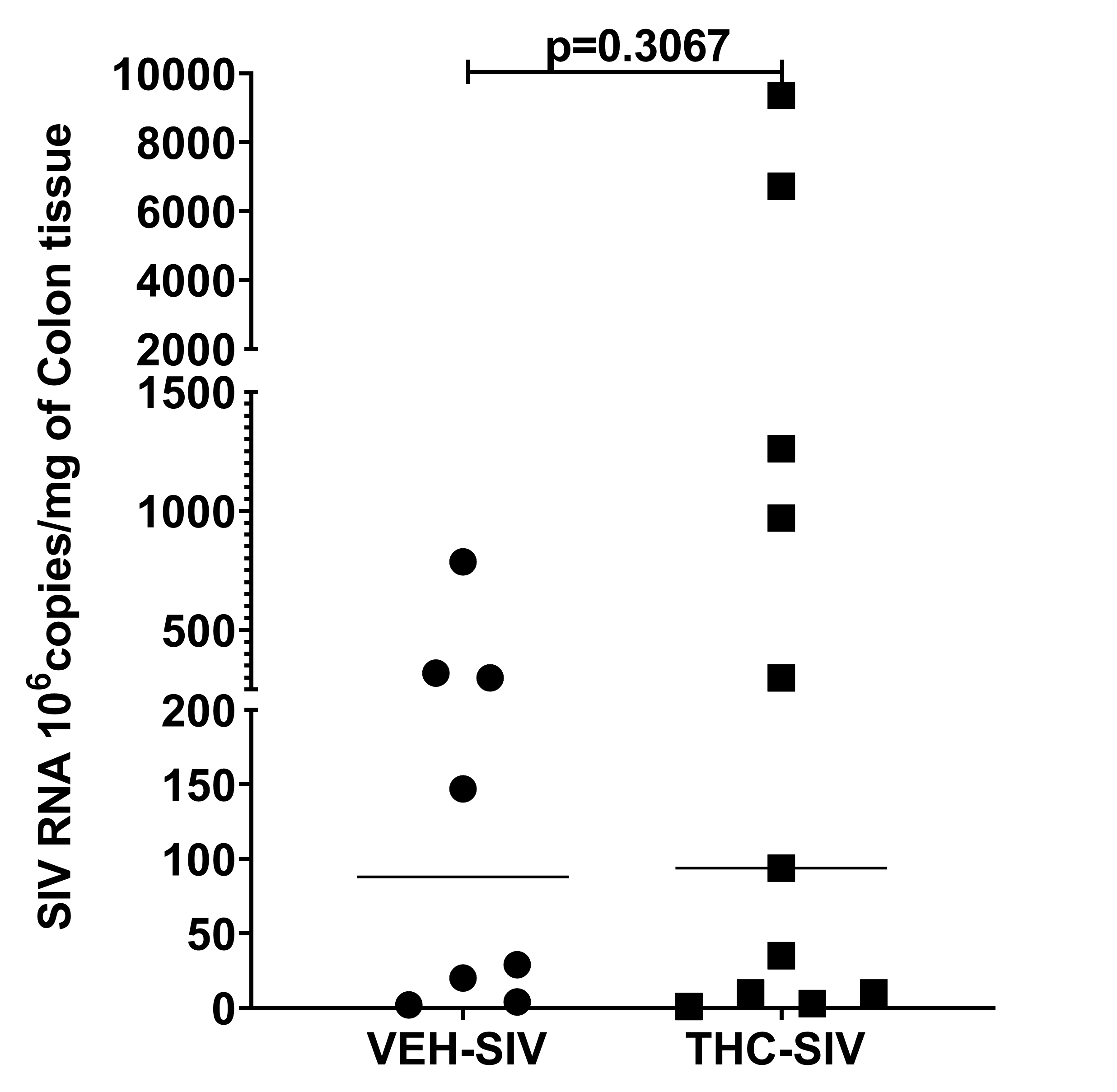


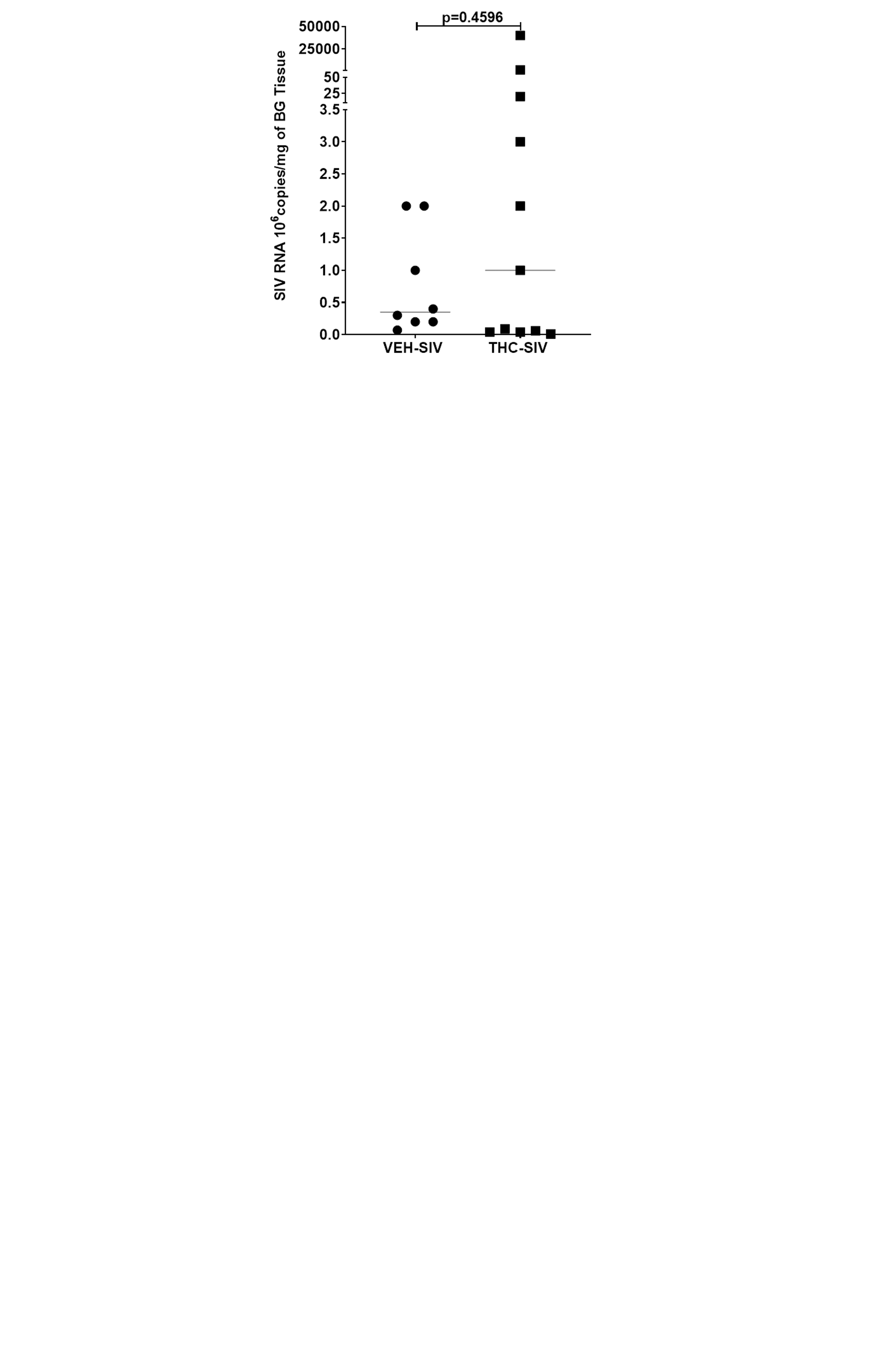


Additional file 1: Fig. S2 QQ plots showing normal distribution of WFS1 (A) and CRYM (B) confocal image quantitation data


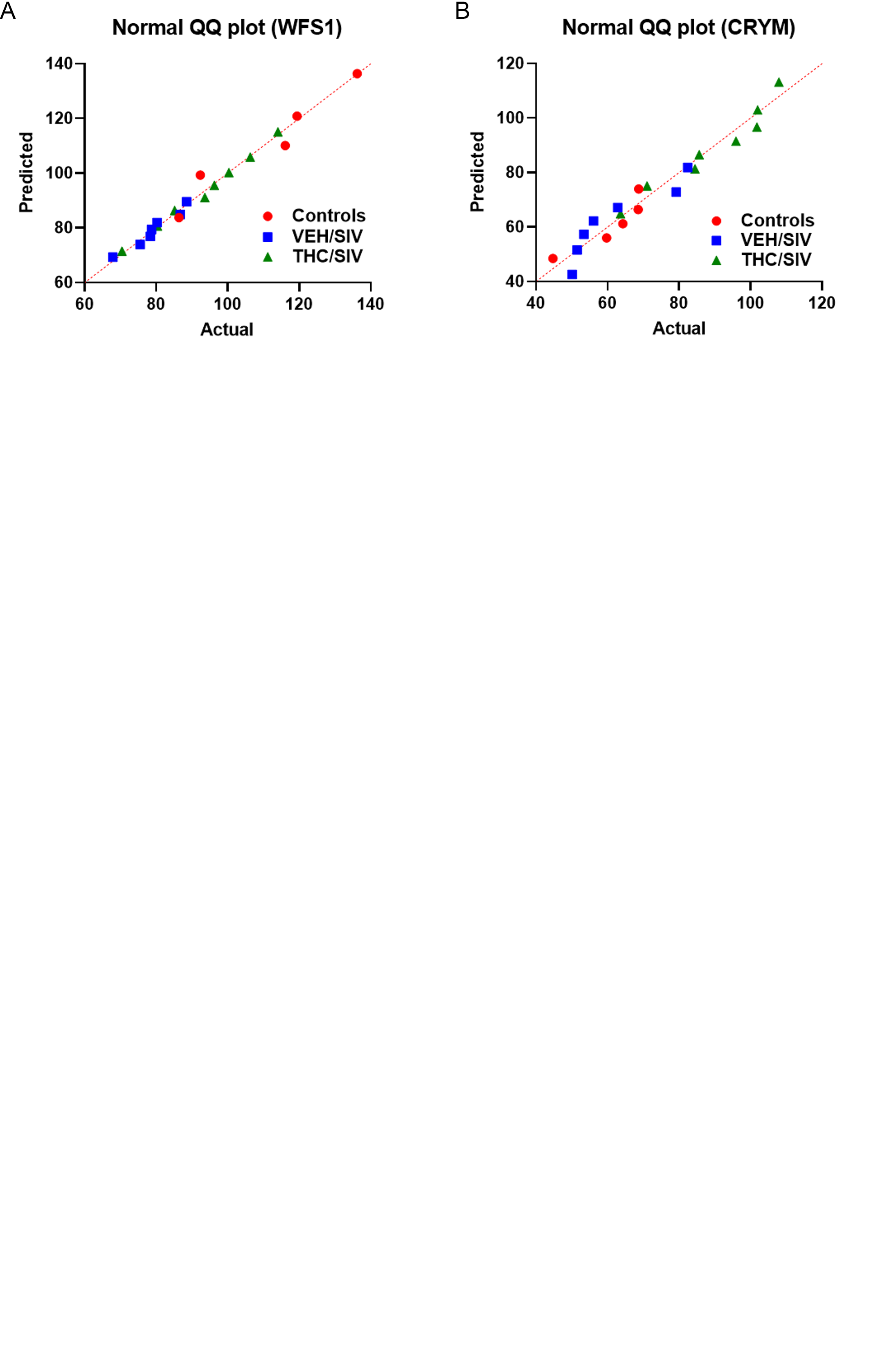


**Additional file 1: Fig. S3** Cannabinoid receptor 1 (CB1R) (**A**) and 2 (CB2R) (**B**) is abundantly expressed in *in vitro* cultured HCN2 neuronal cells. Both panels involve dual labels with CB1R (**A**) and CB2R (**B**) in red and topro3 for nuclear staining in blue.


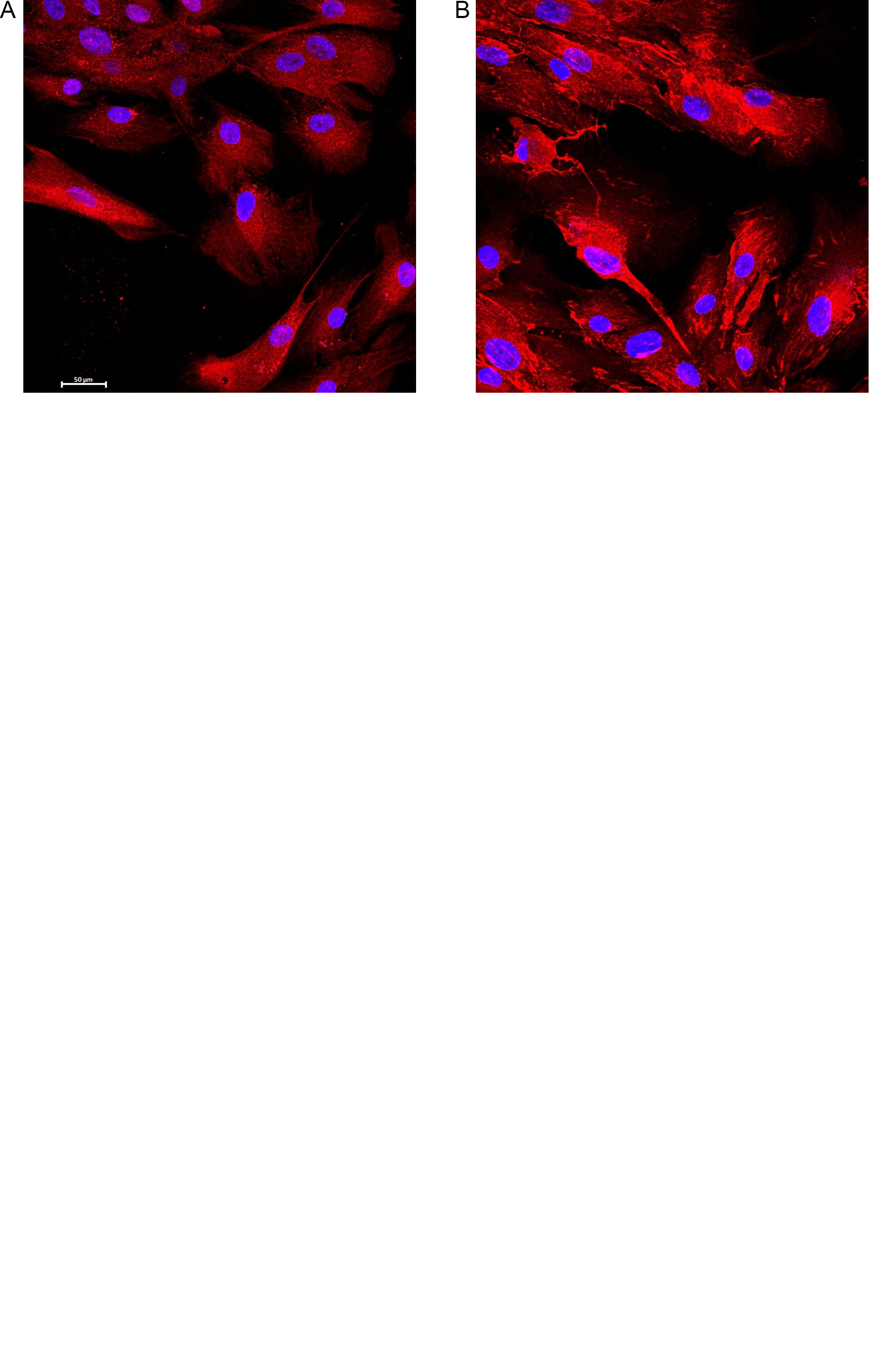


Table S1.

Type or paste caption here. Create a page break and paste in the Table above the caption.

<insert Table S1 here followed by a page break >

Table S2.

Type or paste caption here. Create a page break and paste in the Table above the caption.

<insert Table S2 here followed by a page break >

**Additional file 1: Fig. S4** Concentrations of trans-urocanate (A), xanthurenate (B) and other tryptophan metabolites (C-I) that showed statistically significant increase or decrease in plasma of uninfected control RMs and chronically SIV-infected RMs administered vehicle or delta-9-tetrahyrdocannabinol.


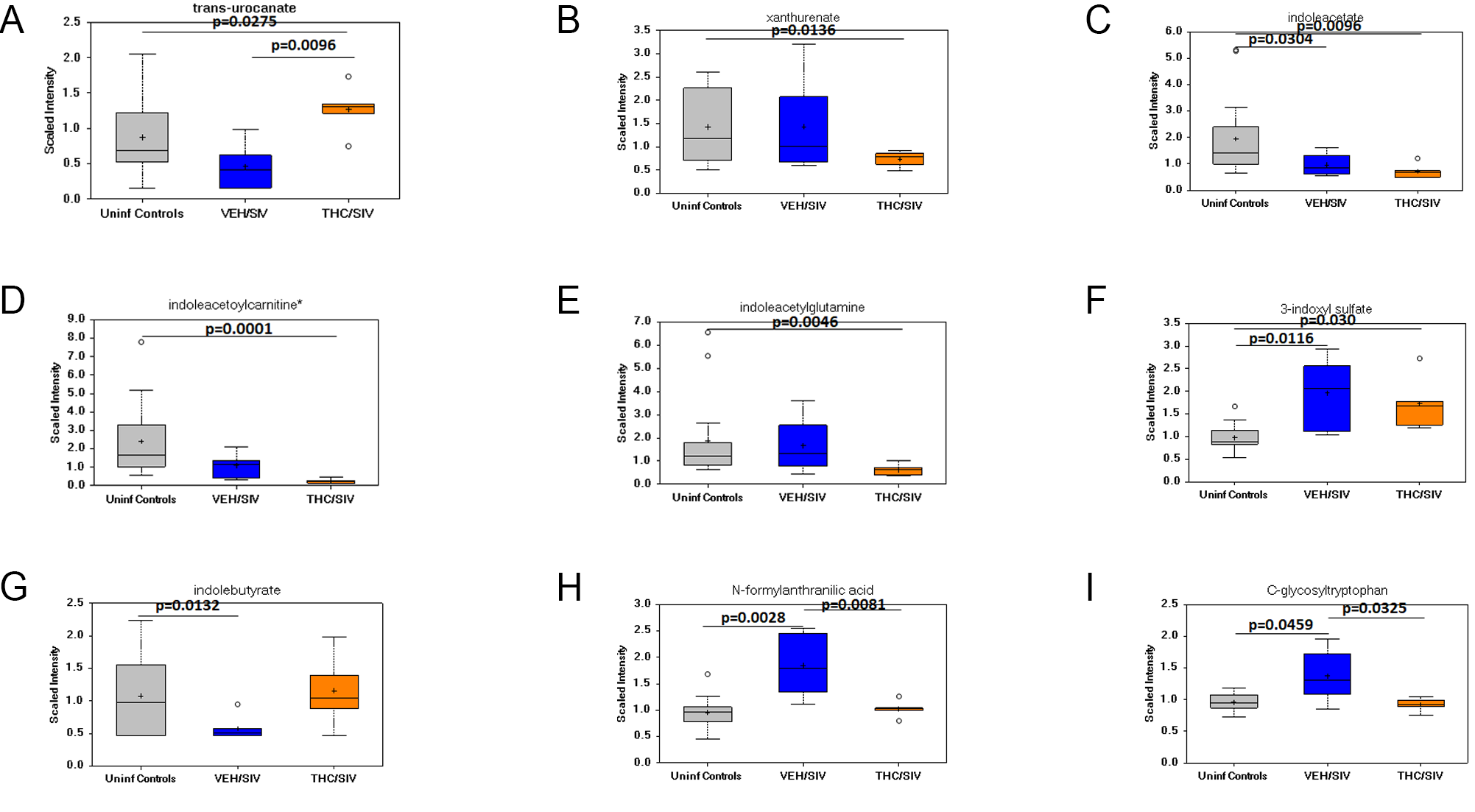


**Additional file 1: Fig. S5** Relative abundance of seven phenyllactate dehydratase gene cluster or its homolog encoding *Clostridia* and *Peptostreptococcus* species that were detected in colonic contents of THC/SIV (**A**) and VEH/SIV (**B**) relative to uninfected control RMs and in THC/SIV relative to VEH/SIV RMs (**C**). (*) indicates p<0.05.


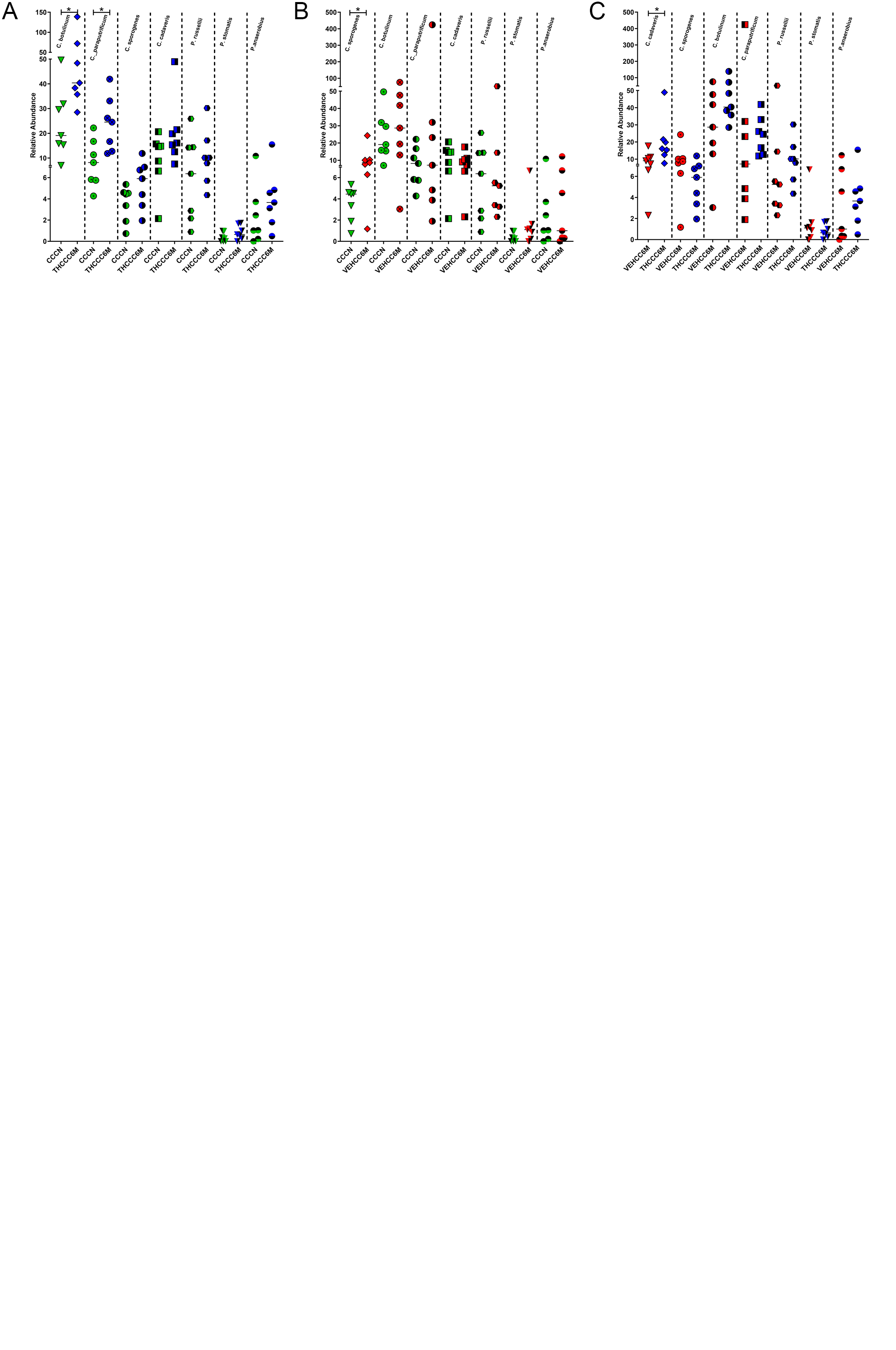


**Additional file 1: Fig. S6** Relative abundance of statistically significant *Ruminococcus* and *Lachnospira* species that were detected in colonic contents of THC/SIV (**A, and D**) and VEH/SIV (**B and E**) relative to uninfected control RMs, and in THC/SIV relative to VEH/SIV RMs (**C and F**).


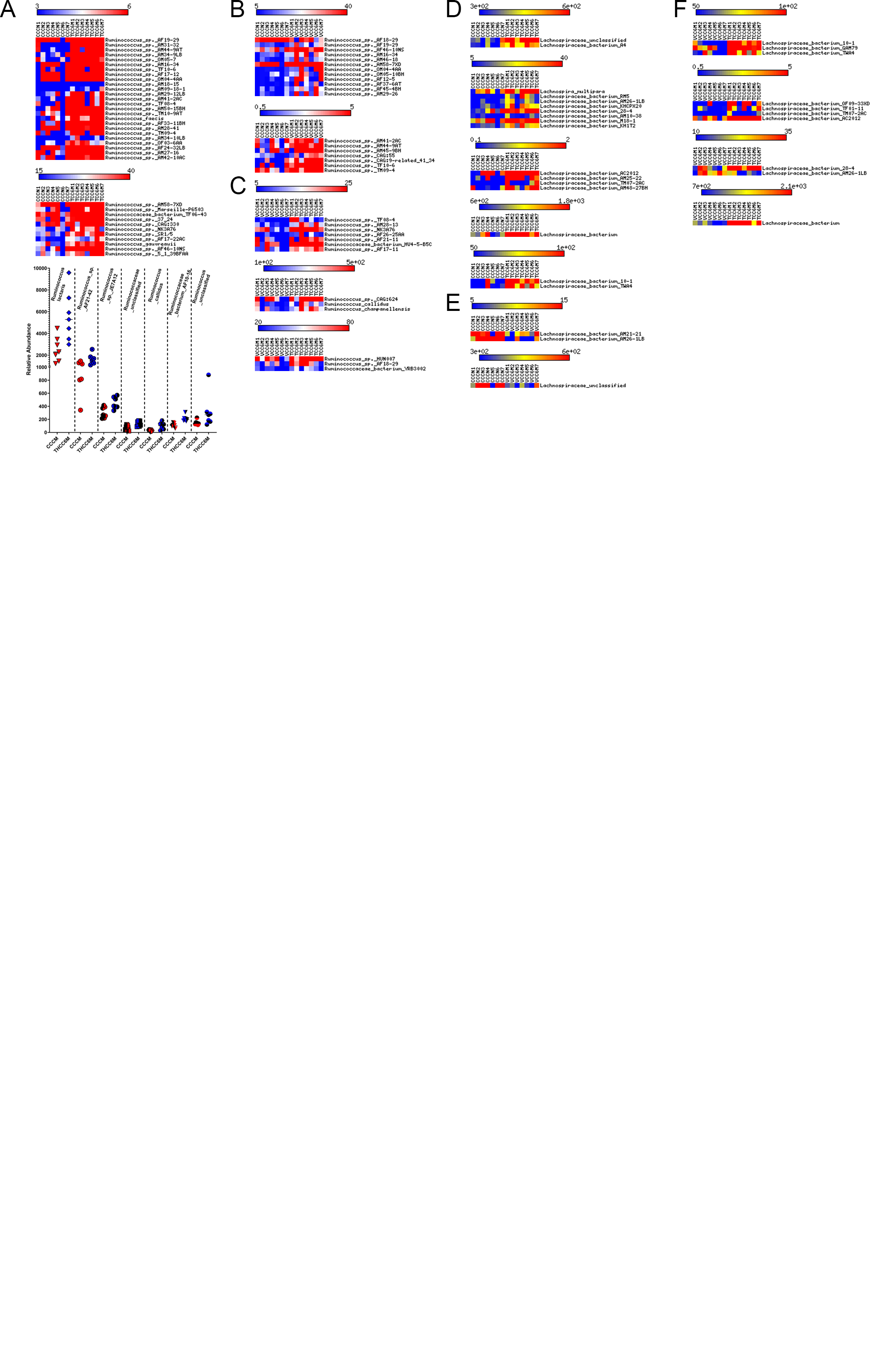


**Additional file 1: Fig. S7** Linear discriminant analysis effect size (LEfSe) analysis were used to generate the cladograms (**A**-**C**) and LDA scores (**D**-**F**) to show taxa differences that were detected in colonic contents of VEH/SIV (**A** and **D**) and THC/SIV (**B** and **E**) relative to uninfected control RMs, and THC/SIV relative to VEH/SIV RMs (**C** and **F**).

**Additional file 1: Fig. S7** Linear discriminant analysis effect size (LEfSe) analysis were used to generate the cladograms (**A**-**C**) and LDA scores (**D**-**F**) to show taxa differences that were detected in colonic contents of VEH/SIV (**A** and **D**) and THC/SIV (**B** and **E**) relative to uninfected control RMs, and THC/SIV relative to VEH/SIV RMs (**C** and **F**).


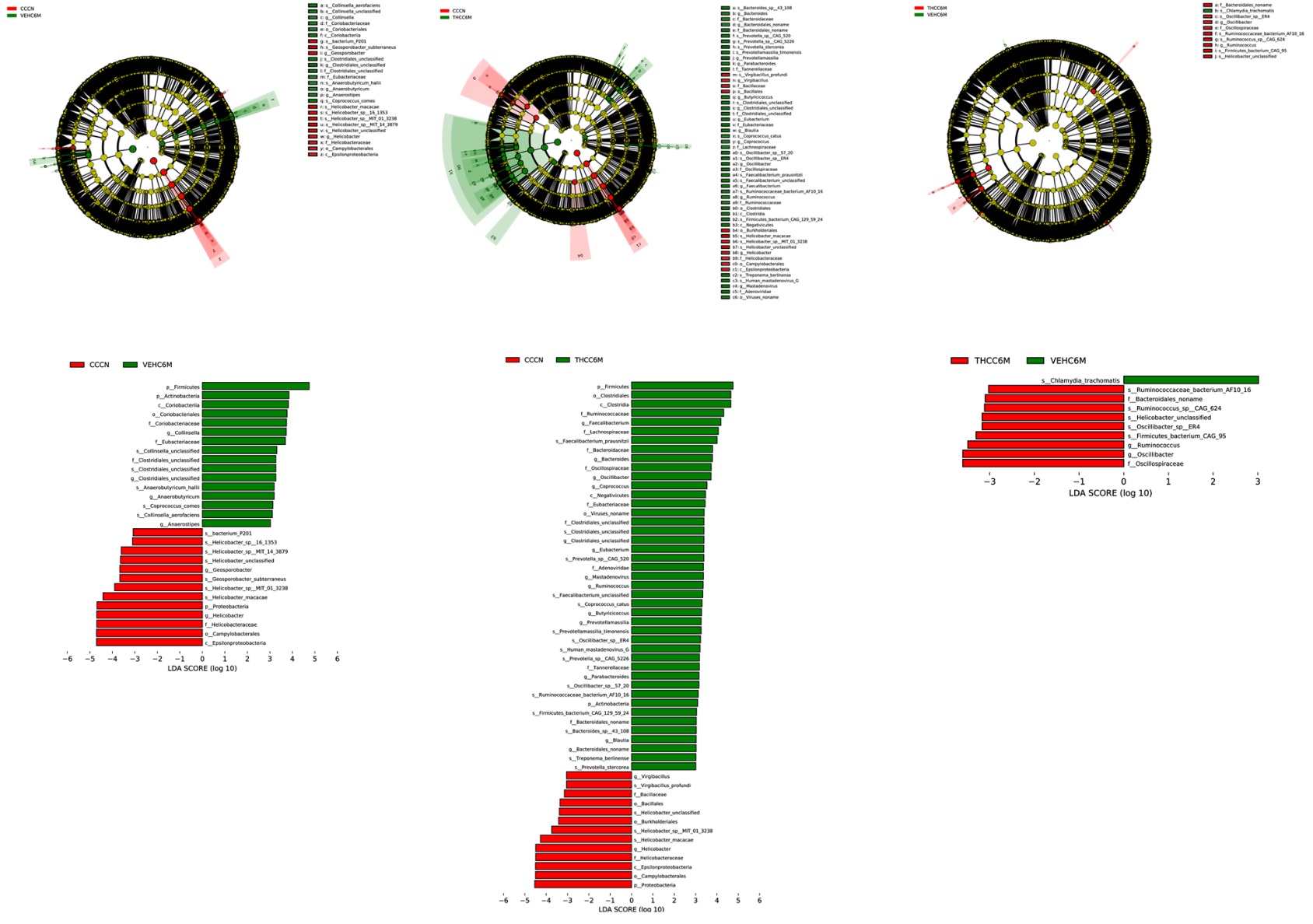


**A**

**B**

**C**

**D**

**E**

**F**
