# Supplementary material for: Cannabinoids modulate the microbiota–gut–brain axis in HIV/SIV infection by reducing neuroinflammation and dysbiosis while concurrently elevating endocannabinoid and indole-3-propionate levels
Source: J Neuroinflammation. 2023 Mar 8;20:62. doi: 10.1186/s12974-023-02729-6 (PMC9993397; doi:10.1186/s12974-023-02729-6)
Supplement: Supplementary file 2 — Additional file 2: Table S1. List of key immune response genes found to be upregulated exclusively in BG of VEH/SIV compared to uninfected control RMs. Table S2. List of downregulated genes in BG of VEH/SIV compared to uninfected control RMs. Table S3. List of upregulated genes in BG of THC/SIV compared to uninfected control RMs. Table S4. List of downregulated genes in BG of THC/SIV compared to control RMs. Table S5. IFN-induced and immune response genes in BG of VEH/SIV and THC/SIV relative to uninfected RMs. Table S6. A List of upregulated and unique genes in basal ganglia of THC/SIV compared to VEH/SIV RMs. B List of downregulated and unique genes in basal ganglia of THC/SIV compared to VEH/SIV RMs. [file 12974_2023_2729_MOESM2_ESM.docx]

**Supplementary Materials for**

**Cannabinoids modulate the microbiota-gut-brain axis in HIV/SIV infection through reducing neuroinflammation and dysbiosis while concurrently elevating endocannabinoid and indole-3-propionate levels**

Marina McDew-White, Eunhee Lee, Lakmini S. Premadasa, Xavier Alvarez, Chioma M. Okeoma, Mahesh Mohan

*Corresponding author. Email: [mmohan@txbiomed.org](mailto:mmohan@txbiomed.org)

**This file includes:**

Supplementary Tables 1 to 6

**Supplementary Tables**

**Additional file 2: Table S1** List of key immune response genes found to be upregulated exclusively in BG of VEH/SIV compared to uninfected control rhesus macaques.

| **Gene Symbol** | **Gene Name** | **Control**  **Read Count** | **VEH/SIV**  **Read Count** | **Fold Change** | **P value** |
| --- | --- | --- | --- | --- | --- |
| APOBEC3H | apolipoprotein B mRNA editing enzyme catalytic subunit 3H | 1.3 | 31.9 | 24.6 | 1.85E-02 |
| BST2 | bone marrow stromal cell antigen 2 | 181.1 | 1511.7 | 8.3 | 2.34E-03 |
| CCL2 | C-C motif chemokine ligand 2 | 4.1 | 24.8 | 6.1 | 2.08E-02 |
| CD74 | CD74 molecule | 836.4 | 2171.9 | 2.6 | 4.77E-02 |
| CXCL10 | C-X-C motif chemokine ligand 10 | 11.5 | 73.7 | 6.4 | 1.70E-03 |
| DDX58 | DExD/H-box helicase 58 | 67.8 | 607.7 | 9.0 | 9.17E-03 |
| GPR65 | G protein-coupled receptor 65 | 3.6 | 17.0 | 4.7 | 3.82E-02 |
| HERC5 | HECT and RLD domain containing E3 ubiquitin protein ligase 5 | 17.7 | 195.0 | 11.0 | 2.52E-05 |
| IFIT1 | interferon induced protein with tetratricopeptide repeats 1 | 360.8 | 2497.0 | 6.9 | 2.36E-03 |
| IRF1 | interferon regulatory factor 1 | 47.5 | 160.7 | 3.4 | 7.59E-03 |
| IRF7 | interferon regulatory factor 7 | 14.3 | 364.8 | 25.5 | 4.99E-03 |
| ISG15 | ISG15 ubiquitin-like modifier | 58.1 | 2739.5 | 47.1 | 1.53E-05 |
| ISG20 | interferon stimulated exonuclease gene 20 | 11.3 | 101.1 | 8.9 | 1.00E-02 |
| MX1 | MX dynamin like GTPase 1 | 298.3 | 5557.5 | 18.6 | 3.66E-06 |
| NLRC5 | NLR family CARD domain containing 5 | 25.1 | 264.2 | 10.5 | 7.13E-06 |
| PSMB8 | proteasome subunit beta 8 | 53.7 | 290.2 | 5.4 | 9.50E-04 |
| PSMB9 | proteasome subunit beta 9 | 79.0 | 446.1 | 5.6 | 6.16E-04 |
| S100A4 | S100 calcium binding protein A4 | 18.0 | 300.0 | 16.7 | 2.55E-02 |
| PTGES | prostaglandin E synthase | 7.5 | 22.1 | 2.9 | 3.47E-02 |
| SAMD9L | sterile alpha motif domain containing 9 like | 66.5 | 354.3 | 5.3 | 1.86E-02 |
| SECTM1 | secreted and transmembrane 1 | 15.2 | 61.1 | 4.0 | 2.72E-02 |
| SP140 | SP140 nuclear body protein | 2.2 | 12.4 | 5.7 | 4.85E-02 |
| STAT2 | signal transducer and activator of transcription 2 | 420.8 | 1063.3 | 2.5 | 4.66E-02 |
| TES | testin LIM domain protein | 22.6 | 68.4 | 3.0 | 4.17E-02 |
| TMEM173 | transmembrane protein 173 | 21.5 | 58.0 | 2.7 | 4.68E-02 |
| TRIM21 | tripartite motif containing 21 | 33.7 | 162.1 | 4.8 | 8.71E-04 |
| XAF1 | XIAP associated factor 1 | 7.0 | 103.1 | 14.8 | 3.13E-03 |
| ZC3HAV1 | zinc finger CCCH-type containing, antiviral 1 | 15.6 | 101.2 | 6.5 | 4.70E-03 |

**Additional file 2: Table S2** List of downregulated genes in BG of VEH/SIV compared to uninfected control rhesus macaques.

| **Gene Symbol** | **Gene Name** | **Control**  **Read Count** | **VEH/SIV**  **Read Count** | **Fold Change** | **P value** |
| --- | --- | --- | --- | --- | --- |
| CALB1 | calbindin | 1747.0 | 595.3 | -2.9 | 2.16E-02 |
| CCKBR | cholecystokinin B receptor | 182.9 | 70.5 | -2.6 | 3.02E-02 |
| CCN3/NOV | nephroblastoma overexpressed | 250.5 | 80.6 | -3.1 | 4.39E-02 |
| CDH9 | cadherin 9 | 167.3 | 66.7 | -2.5 | 4.20E-02 |
| CMTM8 | CKLF like MARVEL transmembrane domain containing 8 | 149.7 | 48.3 | -3.1 | 1.59E-02 |
| CNIH3 | cornichon family AMPA receptor auxiliary protein 3 | 142.8 | 54.2 | -2.6 | 4.60E-02 |
| DDC | dopa decarboxylase | 12.3 | 2.3 | -5.4 | 1.78E-02 |
| EIF4E1B | eukaryotic translation initiation factor 4E family member 1B | 13.1 | 1.7 | -7.7 | 1.61E-02 |
| FEZF2 | FEZ family zinc finger 2 | 234.6 | 79.7 | -2.9 | 1.27E-02 |
| FFAR4 | free fatty acid receptor 4 | 25.2 | 6.3 | -4.0 | 2.84E-02 |
| GABRA5 | gamma-aminobutyric acid type A receptor alpha5 subunit | 1431.5 | 562.4 | -2.5 | 3.36E-02 |
| GDA | guanine deaminase | 2390.4 | 642.9 | -3.7 | 4.47E-02 |
| GLRA2 | glycine receptor alpha 2 | 327.8 | 90.6 | -3.6 | 2.47E-03 |
| GRP | gastrin releasing peptide | 35.9 | 2.3 | -15.4 | 1.63E-02 |
| HTR3A | 5-hydroxytryptamine receptor 3A | 52.3 | 7.4 | -7.1 | 3.62E-02 |
| KCNB2 | potassium voltage-gated channel subfamily B member 2 | 70.5 | 29.4 | -2.4 | 4.97E-02 |
| MGAT4C | MGAT4 family member C | 49.5 | 14.0 | -3.5 | 2.27E-02 |
| NPAS4 | neuronal PAS domain protein 4 | 46.9 | 19.1 | -2.5 | 3.87E-02 |
| NPY1R | neuropeptide Y receptor Y1 | 319.2 | 137.2 | -2.3 | 4.18E-02 |
| NR4A3 | nuclear receptor subfamily 4 group A member 3 | 130.6 | 49.6 | -2.6 | 3.50E-02 |
| NXNL2 | nucleoredoxin like 2 | 52.1 | 15.7 | -3.3 | 1.54E-02 |
| NXPH4 | neurexophilin 4 | 140.2 | 43.5 | -3.2 | 1.00E-02 |
| OLFM4 | olfactomedin 4 | 43.9 | 13.0 | -3.4 | 1.10E-02 |
| PAH | phenylalanine hydroxylase | 23.7 | 5.8 | -4.1 | 2.28E-02 |
| RAPGEF4 | Rap guanine nucleotide exchange factor 4 | 5000.8 | 1475.7 | -3.4 | 9.50E-03 |
| RBP4 | retinol binding protein 4 | 297.3 | 110.1 | -2.7 | 1.83E-02 |
| SLC22A8 | solute carrier family 22 member 8 | 29.6 | 0.6 | -49.1 | 1.37E-02 |
| SNCA | synuclein alpha | 1435.1 | 643.7 | -2.2 | 3.79E-02 |
| SSTR2 | somatostatin receptor 2 | 232.6 | 69.9 | -3.3 | 1.03E-02 |
| SSTR4 | somatostatin receptor 4 | 33.0 | 7.5 | -4.4 | 1.06E-02 |
| TMEFF2 | transmembrane protein with EGF like and two follistatin like domains 2 | 777.9 | 328.5 | -2.4 | 3.87E-02 |
| TOX3 | TOX high mobility group box family member 3 | 110.4 | 34.2 | -3.2 | 1.12E-02 |
| UNC13C | unc-13 homolog C | 1397.9 | 373.4 | -3.7 | 5.87E-03 |
| ZDHHC23 | zinc finger DHHC-type containing 23 | 126.3 | 44.5 | -2.8 | 4.27E-02 |

**Additional file 2: Table S3** List of upregulated genes in BG of THC/SIV compared to uninfected control RMs.

| **Gene Symbol** | **Gene Name** | **Control Read Count** | **THC/SIV**  **Read Count** | **Fold Change** | **P value** |
| --- | --- | --- | --- | --- | --- |
| ACHE | acetylcholinesterase (Cartwright blood group) | 625.6 | 1069.7 | 1.7 | 4.60E-02 |
| ADCY4 | adenylate cyclase 4 | 21.2 | 58.8 | 2.8 | 3.32E-02 |
| CAVIN3 | caveolae associated protein 3 | 104.7 | 178.2 | 1.7 | 4.30E-02 |
| CDKN2A | tumor suppressor ARF-like | 4.4 | 27.8 | 6.3 | 1.41E-02 |
| COL15A1 | collagen type XV alpha 1 chain | 24.0 | 59.7 | 2.5 | 4.48E-02 |
| DDX60 | DExD/H-box helicase 60 | 21.4 | 171.2 | 8.0 | 6.92E-03 |
| FGR | FGR proto-oncogene, Src family tyrosine kinase | 60.1 | 143.6 | 2.4 | 8.95E-03 |
| GPRC5D | G protein-coupled receptor class C group 5 member D | 4.9 | 12.8 | 2.6 | 4.59E-02 |
| HRASLS2 | HRAS like suppressor 2 | 11.3 | 72.7 | 6.4 | 1.06E-03 |
| ICAM3 | intercellular adhesion molecule 3 | 125.5 | 240.3 | 1.9 | 1.95E-02 |
| NOS3 | nitric oxide synthase 3 | 103.0 | 215.9 | 2.1 | 3.53E-02 |
| PKN3 | protein kinase N3 | 34.9 | 66.4 | 1.9 | 3.18E-02 |
| PML | promyelocytic leukemia | 231.1 | 549.0 | 2.4 | 3.68E-02 |
| PRSS36 | serine protease 36 | 10.8 | 30.4 | 2.8 | 2.71E-02 |
| RHBDF1 | rhomboid 5 homolog 1 | 131.0 | 269.0 | 2.1 | 4.17E-02 |
| SCARF1 | scavenger receptor class F member 1 | 20.9 | 44.1 | 2.1 | 3.57E-02 |
| ST6GALNAC1 | ST6 N-acetylgalactosaminide alpha-2,6-sialyltransferase 1 | 17.0 | 37.5 | 2.2 | 1.48E-02 |
| TGM2 | transglutaminase 2 | 70.7 | 157.5 | 2.2 | 2.17E-02 |
| TK1 | thymidine kinase 1 | 8.2 | 34.8 | 4.3 | 3.39E-02 |
| TMEM106A | transmembrane protein 106A | 4.0 | 15.4 | 3.9 | 1.52E-02 |
| TMEM114 | transmembrane protein 114 | 2.6 | 11.0 | 4.2 | 4.76E-02 |
| TMEM156 | transmembrane protein 156 | 16.4 | 36.9 | 2.3 | 4.03E-02 |
| TMPRSS2 | transmembrane serine protease 2 | 2.0 | 53.0 | 26.0 | 2.24E-02 |
| TMPRSS9 | transmembrane serine protease 9 | 24.6 | 97.6 | 4.0 | 2.44E-02 |
| TNFSF10 | TNF superfamily member 10 | 227.3 | 457.8 | 2.0 | 1.89E-02 |
| TRIM22 | tripartite motif containing 22 | 370.7 | 737.9 | 2.0 | 2.82E-02 |
| TRIM5 | tripartite motif containing 5 | 51.2 | 138.3 | 2.7 | 4.20E-02 |
| TRIM56 | tripartite motif containing 56 | 86.6 | 161.7 | 1.9 | 4.55E-02 |
| WFS1 | wolframin ER transmembrane glycoprotein | 2817.2 | 4519.4 | 1.6 | 3.94E-02 |
| XAF1 | XIAP associated factor 1 | 7.3 | 46.8 | 6.4 | 1.05E-02 |
| APOBEC3B-like | DNA dC->dU-editing enzyme APOBEC-3B-like | 15.7 | 72.1 | 4.6 | 3.33E-02 |

**Additional file 2: Table S4** List of downregulated genes in BG of THC/SIV compared to control RMs.

| **Gene Symbol** | **Gene Name** | **Control**  **Read Count** | **THC/SIV**  **Read Count** | **Fold Change** | **P value** |
| --- | --- | --- | --- | --- | --- |
| ADD3 | adducin 3 | 2564.2 | 1304.8 | -2.0 | 3.18E-02 |
| ANGPT1 | angiopoietin 1 | 260.1 | 129.4 | -2.0 | 1.05E-02 |
| ANOS1 | anosmin 1 | 133.7 | 61.0 | -2.2 | 3.56E-02 |
| ATP8 | ATP synthase F0 subunit 8 | 79.2 | 34.0 | -2.3 | 3.48E-02 |
| CNTN1 | contactin 1 | 3577.6 | 1788.3 | -2.0 | 2.15E-02 |
| CSMD3 | CUB and Sushi multiple domains 3 | 241.8 | 124.1 | -1.9 | 4.16E-02 |
| CTNNA3 | catenin alpha 3 | 167.4 | 67.5 | -2.5 | 4.89E-02 |
| DPYD | dihydropyrimidine dehydrogenase | 102.9 | 57.0 | -1.8 | 4.97E-02 |
| FAT4 | FAT atypical cadherin 4 | 238.6 | 124.2 | -1.9 | 3.53E-02 |
| FEM1B | fem-1 homolog B | 422.1 | 218.1 | -1.9 | 3.80E-02 |
| HDAC9 | histone deacetylase 9 | 191.7 | 80.2 | -2.4 | 1.84E-02 |
| LMBRD2 | LMBR1 domain containing 2 | 196.8 | 91.5 | -2.1 | 3.39E-02 |
| LRP1B | LDL receptor related protein 1B | 390.8 | 174.6 | -2.2 | 4.14E-02 |
| MANEA | mannosidase endo-alpha | 219.8 | 114.3 | -1.9 | 4.98E-02 |
| MND1 | tripartite motif containing 2 | 1203.5 | 586.0 | -2.1 | 2.68E-02 |
| MTURN | maturin, neural progenitor differentiation regulator homolog | 933.5 | 409.4 | -2.3 | 1.75E-02 |
| ND4L | NADH-ubiquinone oxidoreductase chain 4L | 16199.4 | 6882.8 | -2.4 | 3.63E-02 |
| NR3C1 | nuclear receptor subfamily 3 group C member 1 | 368.7 | 192.9 | -1.9 | 4.76E-02 |
| NRIP1 | nuclear receptor interacting protein 1 | 185.6 | 95.3 | -1.9 | 4.52E-02 |
| PCSK1 | proprotein convertase subtilisin/kexin type 1 | 377.4 | 123.7 | -3.1 | 3.54E-02 |
| PLA2G4A | phospholipase A2 group IVA | 32.1 | 12.9 | -2.5 | 2.77E-02 |
| PRSS12 | serine protease 12 | 106.4 | 52.2 | -2.0 | 1.63E-02 |
| PTPRK | protein tyrosine phosphatase, receptor type K | 77.5 | 40.7 | -1.9 | 4.21E-02 |
| RAB23 | RAB23, member RAS oncogene family | 303.4 | 166.1 | -1.8 | 3.71E-02 |
| RASA2 | RAS p21 protein activator 2 | 104.2 | 50.8 | -2.1 | 3.30E-02 |
| SEMA3E | semaphorin 3E | 190.3 | 92.4 | -2.1 | 4.10E-02 |
| SLC38A2 | solute carrier family 38 member 2 | 373.7 | 189.1 | -2.0 | 3.81E-02 |
| SLC5A3 | solute carrier family 5 member 3 | 68.4 | 28.9 | -2.4 | 4.31E-02 |
| SLC7A11 | solute carrier family 7 member 11 | 192.8 | 82.4 | -2.3 | 2.87E-02 |
| SLIT2 | slit guidance ligand 2 | 287.0 | 161.4 | -1.8 | 4.53E-02 |
| SMAD9 | SMAD family member 9 | 217.4 | 116.6 | -1.9 | 2.60E-02 |
| SYT14 | synaptotagmin 14 | 94.2 | 32.6 | -2.9 | 3.19E-03 |
| TAB3 | TGF-beta activated kinase 1 (MAP3K7) binding protein 3 | 89.2 | 42.3 | -2.1 | 4.58E-02 |
| TCF4 | transcription factor 4 | 616.8 | 342.7 | -1.8 | 3.74E-02 |
| UNC13C | unc-13 homolog C | 1461.4 | 687.6 | -2.1 | 2.18E-02 |

**Additional file 2: Table S5** Read counts and fold change of IFN induced and immune response genes in BG of VEH/SIV and THC/SIV relative to uninfected RMs.

| **Gene Symbol** | **Gene Name** | **Control**  **Read Count** | **THC/SIV**  **Read Count** | **VEH/SIV**  **Read Count** | **Fold Change**  **THC/SIV** | **Fold Change**  **VEH/SIV** |
| --- | --- | --- | --- | --- | --- | --- |
| IFI44L | Interferon induced protein 44 like | 25 | 147 | 390 | 5.8 | 16.1 |
| IFI44 | Interferon induced protein 44 | 55 | 703 | 1060 | 12.8 | 20.1 |
| IFIH1 | Interferon induced with helicase C domain 1 | 84 | 409 | 522 | 4.9 | 6.5 |
| IFIT3 | Interferon induced protein with tetratricopeptide repeats 3 | 277 | 1569 | 2193 | 5.7 | 8.3 |
| STAT1 | Signal transducer and activator of transcription 1 | 1193 | 5331 | 6222 | 4.5 | 5.5 |
| OAS2 | 2'-5'-oligoadenylate synthetase 2 | 34 | 186 | 655 | 5.5 | 20.3 |
| IFI6 | Interferon alpha inducible protein 6 | 1730 | 42762 | 33819 | 24.7 | 20.4 |
| TRIM14 | Tripartite motif containing 14 | 88 | 208 | 317 | 2.4 | 3.8 |
| IRX1 | Iroquois homeobox 1 | 6 | 15 | 21 | 2.7 | 3.9 |
| B2M | Beta-2-microglobulin | 2828 | 9344 | 13669 | 3.3 | 5.1 |
| APOBEC3F | Apolipoprotein B mRNA editing enzyme catalytic subunit 3D | 120 | 295 | 276 | 2.5 | 2.4 |
| BTN3A1 | Butyrophilin subfamily 3-member A2 | 34 | 100 | 156 | 2.9 | 4.8 |
| RNF213 | Ring finger protein 213 | 477 | 933 | 1574 | 2.0 | 3.4 |
| TRIM25 | Tripartite motif containing 25 | 100 | 199 | 318 | 2.0 | 3.3 |
| HELZ2 | Helicase with zinc finger 2 | 57 | 504 | 503 | 8.8 | 9.2 |
| HERC6 | HECT and RLD domain containing E3 ubiquitin protein ligase family member 6 | 57 | 413 | 779 | 7.3 | 14.4 |
| DHX58 | DExH-box helicase 58 | 119 | 977 | 977 | 8.2 | 8.6 |
| PARP9 | Poly (ADP-ribose) polymerase family member 9 | 80 | 231 | 285 | 2.9 | 3.7 |
| MAMU-A3 | Macaca mulatta HLA class I histocompatibility antigen, A-11 alpha chain mRNA | 166 | 553 | 927 | 3.3 | 5.8 |
| BTN3A3 | Butyrophilin subfamily 3-member A3 | 131 | 321 | 456 | 2.4 | 3.6 |
| CCDC69 | Coiled-coil domain containing 69 | 62 | 121 | 290 | 1.9 | 4.9 |
| VCAM1 | Vascular cell adhesion molecule 1 | 92 | 217 | 249 | 2.3 | 2.8 |

**Additional file 2: Table S6A** List of upregulated and unique genes in basal ganglia of THC/SIV compared to VEH/SIV RMs.

| **Gene Symbol** | **Gene Name** | **VEH/SIV**  **Read Count** | **THC/SIV**  **Read Count** | **Fold Change** | **P value** |
| --- | --- | --- | --- | --- | --- |
| SLC7A4 | SLC7A4 | 79.3 | 196.3 | 2.5 | 4.65E-02 |
| CARTPT | CART prepropeptide | 25.1 | 169.0 | 6.7 | 2.33E-02 |
| CRYM | crystallin mu | 1748.3 | 3607.5 | 2.1 | 4.05E-02 |
| ERN2 | endoplasmic reticulum to nucleus signaling 2 | 4.2 | 16.9 | 4.0 | 2.49E-02 |
| GLRA2 | glycine receptor alpha 2 | 87.3 | 231.9 | 2.7 | 3.11E-02 |
| GRP | gastrin releasing peptide | 2.3 | 16.8 | 7.4 | 1.53E-02 |
| IRX6 | iroquois homeobox 6 | 10.7 | 28.5 | 2.7 | 2.82E-02 |
| RBP4 | retinol binding protein 4 | 106.1 | 306.2 | 2.9 | 1.65E-02 |
| RHEX* | regulator of hemoglobinization and erythroid cell expansion | 2.2 | 14.5 | 6.6 | 9.14E-03 |
| SLC34A2 | solute carrier family 34 member 2 | 3.2 | 12.4 | 3.9 | 4.70E-02 |
| TAC3 | tachykinin 3 | 71.1 | 160.3 | 2.3 | 4.36E-02 |
| TAF7L | TATA-box binding protein associated factor 7 like | 8.9 | 28.4 | 3.2 | 3.74E-02 |
| TH | tyrosine hydroxylase | 9.5 | 31.5 | 3.3 | 2.14E-02 |
| PDE11A | phosphodiesterase 11A | 36.4 | 102.1 | 2.8 | 2.47E-02 |

* denotes gene not unique to THC/SIV RMs

**Additional file 2: Table S6A** List of downregulated and unique genes in basal ganglia of THC/SIV compared to VEH/SIV RMs.

| **Gene Symbol** | **Gene Name** | **VEH/SIV**  **Read Count** | **THC/SIV**  **Read Count** | **Fold Change** | **P value** |
| --- | --- | --- | --- | --- | --- |
| AADAC | arylacetamide deacetylase | 85.60496138 | 0 | 85.6 | 4.74E-02 |
| ADAMTS5 | ADAM metallopeptidase with thrombospondin type 1 motif 5 | 21.79358576 | 3.431641209 | 6.4 | 3.13E-03 |
| ADH7 | alcohol dehydrogenase 7 | 1836.855641 | 0.74833732 | 2454.6 | 2.79E-02 |
| ARG1 | arginase 1 | 2122.1 | 3.5 | 601.8 | 4.97E-02 |
| ANXA8 | annexin A8 | 1994.2 | 2.2 | 911.5 | 4.25E-02 |
| CCDC69 | coiled-coil domain containing 69 | 279.3699752 | 109.8872004 | 2.5 | 2.72E-02 |
| CLDN17 | Claudin 17 | 261.954767 | 0 | 262.0 | 2.70E-02 |
| CLIC5 | chloride intracellular channel 5 | 34.70392043 | 6.907019663 | 5.0 | 3.98E-02 |
| CRCT1 | cysteine rich c-terminal 1 | 2303.255086 | 0 | 2303.3 | 9.31E-03 |
| CRNN | cornulin | 12625.25586 | 0 | 12625.3 | 4.00E-03 |
| CRYBG2 | crystallin beta-gamma domain containing 2 | 284.0033927 | 0 | 284.0 | 2.62E-02 |
| CSRP3 | cysteine and glycine rich protein 3 | 189.0635712 | 0 | 189.1 | 3.17E-02 |
| CWH43 | cell wall biogenesis 43 c-terminal homolog | 227.9177303 | 0 | 227.9 | 2.91E-02 |
| DSG1 | desmoglein 1 | 2087.3 | 1.6 | 1295.4 | 3.72E-02 |
| DSG3 | desmoglein 3 | 2599.163644 | 0 | 2599.2 | 8.57E-03 |
| DUOXA1 | dual oxidase maturation factor 1 | 245.3056367 | 0 | 245.3 | 2.76E-02 |
| EGF | epidermal growth factor | 15.89170936 | 2.538771281 | 6.3 | 3.36E-02 |
| EMP1 | epithelial membrane protein 1 | 19671.0 | 29.5 | 666.8 | 4.88E-02 |
| FAM83C | family with sequence similarity 83 member c | 92.56942716 | 0 | 92.6 | 4.46E-02 |
| FDCSP | follicular dendritic cell secreted protein | 5569.13874 | 0 | 5569.1 | 6.01E-03 |
| FGFBP1 | fibroblast growth factor binding protein 1 | 836.6 | 0.3 | 2584.6 | 2.86E-02 |
| FOLR1 | folate receptor 1 | 45.0 | 10.0 | 4.5 | 1.55E-02 |
| FOXE1 | forkhead box e1 | 78.14754187 | 0 | 78.1 | 4.95E-02 |
| FREM2 | FRAS1 related extracellular matrix 2 | 17.86494576 | 5.743339912 | 3.1 | 4.44E-02 |
| GABRP | gamma-aminobutyric acid type A receptor pi subunit | 944.8 | 0.4 | 2115.8 | 3.13E-02 |
| GJB6 | gap junction protein beta 6 | 460.6 | 191.5 | 2.4 | 2.85E-02 |
| GSG1 | germ cell associated 1 | 978.6 | 1.4 | 717.8 | 4.63E-02 |
| HMCN1 | hemicentin 1 | 104.6 | 40.2 | 2.6 | 4.24E-02 |
| HPSE2 | heparanase 2 | 10.26316802 | 2.297110463 | 4.5 | 4.47E-02 |
| IL36A | interleukin 36 alpha | 338.3804099 | 0 | 338.4 | 2.41E-02 |
| ITGA4* | integrin subunit alpha 4 | 19.5 | 6.4 | 3.0 | 4.24e-02 |
| KIF24 | kinesin family member 24 | 42.73032414 | 13.13435539 | 3.3 | 1.66E-02 |
| KITLG | KIT ligand | 57.51653542 | 22.51457244 | 2.6 | 4.78E-02 |
| KLK12 | kallikrein related peptidase 12 | 246.0948436 | 0 | 246.1 | 2.81E-02 |
| KRT4 | keratin 4 | 153676.5982 | 0.139535744 | 1101342.2 | 1.74E-03 |
| KRT5 | keratin 5 | 28160.18307 | 11.78579932 | 2389.3 | 2.64E-02 |
| KRT6A | keratin 6A | 2434.070653 | 0.460470967 | 5286.0 | 1.97E-02 |
| KRT15 | keratin 15 | 93192.72477 | 1.147741734 | 81196.6 | 4.63E-03 |
| KRT16 | keratin 16 | 36328.32154 | 8.365631117 | 4342.6 | 1.85E-02 |
| KRT23 | keratin 23 | 926.4135311 | 1.04293321 | 888.3 | 4.48E-02 |
| KRT24 | keratin 24 | 1328.63671 | 0.925295252 | 1435.9 | 3.53E-02 |
| KRT76 | keratin 76 | 2372.393347 | 0 | 2372.4 | 9.16E-03 |
| KRT78 | keratin 78 | 6345.953271 | 0 | 6346.0 | 5.63E-03 |
| KRT80 | keratin 80 | 663.3472668 | 0.497491742 | 1333.4 | 3.68E-02 |
| KRTDAP | keratinocyte differentiation associated protein | 2934.184732 | 0 | 2934.2 | 8.25E-03 |
| LIPN | lipase family member N | 7.950396567 | 1.025388741 | 7.8 | 1.48E-02 |
| LTBP2 | latent transforming growth factor beta binding protein 2 | 25.74229401 | 8.503165779 | 3.0 | 3.69E-02 |
| MAMU-B18 | Macaca mulatta patr class I histocompatibility antigen, A-126 alpha chain-like | 241.7 | 64.8 | 3.7 | 4.42E-02 |
| MAMU-DOB | Macaca mulatta patr class I histocompatibility antigen, DO beta | 11.39742233 | 1.446747814 | 7.9 | 1.31E-02 |
| MT4 | metallothionein 4 | 6.517076989 | 0 | 6.5 | 3.51E-02 |
| MUC20 | mucin 20, cell surface associated | 178.8 | 0.2 | 1138.4 | 4.90E-02 |
| MYH2 | myosin heavy chain 2 | 2633.2 | 0.5 | 4970.2 | 1.89E-02 |
| MYH8* | myosin-8 | 177.0 | 0.1 | 1268.2 | 4.90E-02 |
| MYL1 | myosin light chain 1 | 652.0581178 | 0 | 652.1 | 1.74E-02 |
| NKX3-1 | NK3 homeobox 1 | 22.77933832 | 5.752490198 | 4.0 | 1.49E-02 |
| PHLDB2 | pleckstrin homology like domain family B member 2 | 44.77571277 | 16.14849431 | 2.8 | 3.54E-02 |
| PITX2 | paired like homeodomain 2 | 405.3 | 0.2 | 2672.1 | 3.28E-02 |
| PLA2G4A* | phospholipase A2 group IVA | 44.5 | 11.9 | 3.7 | 2.37E-02 |
| RNF222 | ring finger protein 222 | 82.49770325 | 0 | 82.5 | 4.82E-02 |
| SCEL | sciellin | 2224.744845 | 1.925707089 | 1155.3 | 3.72E-02 |
| SERPINB11 | serpin family B member 11 | 407.8276291 | 0 | 407.8 | 2.19E-02 |
| SERPINB2 | serpin family B member 2 | 868.0 | 0.9 | 1003.5 | 4.34E-02 |
| SLC6A14 | solute carrier family 6 member 14 | 265.6 | 0.1 | 1903.8 | 3.94E-02 |
| SLC6A4 | solute carrier family 6 member 4 | 206.4773025 | 0 | 206.5 | 3.07E-02 |
| SPINK5 | serine peptidase inhibitor, Kazal type 5 | 6739.2 | 6.4 | 1057.8 | 3.80E-02 |
| SPINK7 | serine peptidase inhibitor, Kazal type 7 (putative) | 2257.2 | 1.3 | 1782.7 | 3.09E-02 |
| SPRR1A | small proline rich protein 1A | 904.4 | 0 | 904.4 | 1.48E-02 |
| THEMIS | thymocyte selection associated | 11.04386439 | 1.497665115 | 7.4 | 1.94E-02 |
| TMPRSS11A | transmembrane serine protease 11A | 417.4601293 | 0.157080212 | 2657.6 | 3.23E-02 |
| TMPRSS11E | transmembrane serine protease 11E | 200.8842379 | 0.139535744 | 1439.7 | 4.64E-02 |
| TNFRSF19 | TNF receptor superfamily member 19 | 58.1 | 20.8 | 2.8 | 2.57E-02 |
| TNNT3 | troponin T3, fast skeletal type | 531.0169813 | 0.178977999 | 2966.9 | 2.87E-02 |
| ZKSCAN8 | zinc finger with KRAB and SCAN domains 8 | 80.04278124 | 31.85312861 | 2.5 | 4.12E-02 |

* denotes gene not unique to THC/SIV RMs
