# Supplementary material for: Cannabinoids modulate the microbiota–gut–brain axis in HIV/SIV infection by reducing neuroinflammation and dysbiosis while concurrently elevating endocannabinoid and indole-3-propionate levels
Source: J Neuroinflammation. 2023 Mar 8;20:62. doi: 10.1186/s12974-023-02729-6 (PMC9993397; doi:10.1186/s12974-023-02729-6)
Supplement: Supplementary file 3 — Additional file 3: Table S7. Additional Methods. [file 12974_2023_2729_MOESM3_ESM.docx]

**Supplementary Methods for**

**Cannabinoids modulate the microbiota-gut-brain axis in HIV/SIV infection through reducing neuroinflammation and dysbiosis while concurrently elevating endocannabinoid and indole-3-propionate levels**

Marina McDew-White, Eunhee Lee, Lakmini S. Premadasa, Xavier Alvarez, Chioma M. Okeoma, Mahesh Mohan

*Corresponding author. Email: [mmohan@txbiomed.org](mailto:mmohan@txbiomed.org)

**This PDF file includes:**

Supplementary Text- Methods

Supplementary Text

**Additional explanatory text for Methods**

RNA-seq library construction, clustering and sequencing

Transcriptome profiling by RNA-seq and data analysis were performed by Novogene (Sacramento, CA). cDNA library construction and sequencing were performed by Novogene Co. Ltd, Beijing, CA (http://www.novogene.cn/). For library construction, ~3 μg of total RNA from each sample was used to enrich mRNA with the poly-T oligo-attached magnetic beads. The purified mRNA was then randomly cleaved into small fragments using NEBNEXT RNA fragmentation buffer (NEB, MA) following the manufacturer’s instructions. First-strand cDNA was synthesized using random hexamers and M-MuLV Reverse Transcriptase (RNase H-). Second-strand cDNA synthesis by nick translation was subsequently performed using *E coli* DNA polymerase I and RNase H. Remaining overhangs were converted into blunt ends via exonuclease/polymerase activities. The final cDNA library was prepared after a round of purification, terminal repair, A-tailing, ligation of sequencing adapters, size selection and PCR enrichment. The cDNA fragments of preferentially 250−300 bp in length were selected using with AMPure XP system (Beckman Coulter, Beverly, U.S.A.) and PCR amplified using Phusion High-Fidelity DNA polymerase (NEB, Beijing, China), Universal PCR primers and Index (X) Primer. PCR products were then purified using AMPure XP system (Beckman Coulter, U.S.A.), and library quality was assessed on the Agilent Bioanalyzer 2100 system (Agilent Technologies, U.S.A.). The clustering of the index-coded samples was performed on a cBot Cluster Generation System, using TruSeq SR Cluster Kit v3-cBot-HS (Illumina), according to the manufacturer’s instructions. After cluster generation, the library preparations were sequenced on an Illumina Novaseq 6000 platform and 150 bp paired end reads were generated.

*Global micro-RNA and gene expression profiling*

Micro-RNA and mRNA expression profiling were performed using TaqMan OpenArray Human Micro-RNA panels (Thermo Fisher Scientific) and RNA-seq (Novogene Inc), respectively. Briefly, total RNA from OPM tissue was isolated using the miRNeasy total RNA isolation kit (Qiagen Inc, CA) following the manufacturer’s protocol. Approximately 100 ng total RNA was first reverse transcribed using the micro-RNA reverse transcription reaction kit (Life Technologies, Grand Island, NY).

Briefly, two master mixes representing either OpenArray panel (panel A and panel B) were prepared for each RNA sample, which consisted of the following reaction components: 0.75 μL MegaPlex RT primers (10x), 0.15 μL dNTPs with dTTP (100 mM), 1.50 μL MultiScribe™ Reverse Transcriptase (50 U/μL), 0.75 μL 10x RT Buffer, 0.90 μL MgCl_2_ (25 mM), 0.09 μL RNase Inhibitor, 0.35 μL nuclease-free water (20 U/μL). Three microliters of total RNA (100 ng) were loaded into appropriate wells of a 96-well plate together with 4.5 μL of the RT reaction master mix. After a brief spin and 5 min of incubation on ice, samples in the 96-well plate were subjected to the following thermal cycling conditions on the ABI 7900 HT Fast PCR system: standard or max ramp speed, 16 °C for 2 min, 42 °C for 1 min, 50 °C 1 sec (40 cycles), 85 °C 5 min (hold), 23 °C (hold). Immediately after thermal cycling, the 96-well plate containing cDNA was stored at -80 °C.

For pre-amplification, 2.5 μL of the cDNA from each sample was mixed with a total of 22.5 μL of pre-amplification reaction master mix consisting of 12.5 μL TaqMan® PreAmp Master Mix (2x), 2.5 μL Megaplex™ PreAmp Primers (10x) and 7.5 μL nuclease-free water in a 96-well plate. After a brief vortex and spin, samples in the 96-well plate were subjected to the following thermal cycling conditions on the ABI 7900 HT Fast PCR system: standard or max ramp speed, hold 95 °C 10 min; hold 55 °C 2 min; hold 72 °C 2 min; 12 cycles at 95 °C 15 sec and 60 °C 4 min; hold 4 °C. The preamplified product was diluted 40 times by mixing 4 μL of the preamplified product with 156 μL of 0.1x TE pH 8.0 and loaded onto TaqMan® OpenArray® human micro-RNA plates for processing using the QuanStudio™ 12K Flex Real-Time PCR system (Life Technologies).

*Quantitative Real-Time TaqMan and SYBR Green RT-qPCR assay for OpenArray® validation*

Expression of miR-142-3p was quantified in BG tissue using the TaqMan micro-RNA predesigned and preoptimized assays (Thermo Scientific). Approximately 200 ng of total RNA was reverse transcribed using the stem-loop primers provided in the predesigned kit in a total volume of 20 μL. ~4 μL of cDNA was then subjected to 40 cycles of PCR on the ABI 7900 HT Fast PCR System (Thermo Fisher Scientific, Waltham, MA) using the following thermal cycling conditions: 95°C for 10 minutes followed by 40 repetitive cycles of 95°C for 15 sec and 60°C for 1 min. As a normalization control for RNA loading, parallel reactions in triplicate wells to amplify RNU48 were run in the same multi-well plate. Comparative real-time PCR was performed in duplicate wells including no-template controls and relative change in gene expression was calculated using the comparative C_T_ method.

mRNA expression of WFS-1 in BG was quantified by RT-qPCR using the Power SYBR Green RNA to C_T_ kit. (Thermo Fisher). Each RT-qPCR reaction (20 μl) contained the following: 2X Power SYBR Green Master Mix without uracil-N-glycosylase (12.5 μl), forward and reverse primer [WFS1 (For-5’-CAAGTGGCCTGTCTTCGAG -3’, Rev-5’- GTCGTGCTCGATCTTCACAT -3’), GAPDH (For-5’-CAAGAGAGGCATTCTCACCCTGAA-3’, Rev-5’-TGGTGCCAGATCTTCTCCATGTC-3’) and Beta-actin (For-5’-CAACAGCCTCAAGATCGTCAGCAA-3’, Rev-5’-GAGTCCTTCCACGATACCAAAGTTGTC-3’) (200 nM) and ~200 ng of total RNA (4 μl). The PCR amplification was carried out in the ABI 7900 HT sequence detection System (Thermo Fisher) using the default thermal cycling conditions for SYBR Green assays. As a normalization control for RNA loading, parallel reactions in the same multi-well plate were performed using GAPDH and β-Actin. Relative changes in gene expression were calculated using the ΔΔC_T_ method. PCR efficiency analysis was performed using serial 10-fold RNA dilution (500, 50, 5 and 0.5 ng of total RNA). The amplification curves for all assays were linear and based on slope values (-3.15 to -3.24) all assays had 100-102% efficiency.

*Cloning of 3’-UTR of WFS1 mRNA and Dual-Glo luciferase reporter gene assay*

The 3’ UTR of the rhesus macaque WFS1 mRNA contains a single predicted miR-142-3p binding site (TargetScan 7.2). Accordingly, a short 54 nucleotide sequence representing the 3' UTR containing the predicted miR-142-3p site (5’- AGGCGGCGCACUGGCAGUGUGUCACACUGAGCACAG**CACUACA**GGCUGCCUCAU-3’) was synthesized (IDTDNA Technologies Inc., IA) for cloning into the pmirGLO Dual-Luciferase vector (Promega Corp, Madison, WI). A second oligonucleotide with the miRNA binding site deleted (n=7 nucleotides) (5’- AGGCGGCGCACUGGCAGUGUGUCACACUGAGCACAGGGCUGCCUCAU -3’) was also synthesized to serve as a negative control. Both wild type and deleted WFS1 oligonucleotide sequences were synthesized with a *Pme1* site on the 5’ and *Xba1* site on the 3’ end for directional cloning. The pmirGLO vector was first cut with *Pme1* and *Xba1* restriction enzymes, gel purified and ligated with either wild-type sequence containing the miR-142-3p binding site (WFS1-wtUTR) or deleted sequence (WFS1-delUTR). As stated previously, HEK293 cells were used for the reporter assay as these cells abundantly express MMP8 and >95-98% of these cells can be successfully transfected with exogenous nucleic acids using Lipojet® transfection reagent (Signagen). HEK293 cells were plated at a density of 2x10^4^ cells per well of a 96 well plate. At 60-70% confluence cells were co-transfected with ~100 ng WFS1-wtUTR or WFS1-delUTR luciferase reporter vector and 20 and 40 nM FAM-miR-142-3p or negative control (cel-miR-67-3p) mimic using the Lipojet® transfection reagent (Signagen). For miR-142-3p overexpression studies, we used the HCN2 (human cortical neuronal primary cell line). After 72 h, the Dual Glo luciferase assay was performed according to the manufacturer’s recommended protocol (Promega Corp) using the BioTek H4 Synergy plate reader (BioTek, Winooski, VT). The normalized *firefly* to *renilla* ratio was calculated to determine the relative reporter activity. Experiments were performed in 6 replicates and repeated thrice.

Quantification of Endocannabinoid and trytophan metabolites in plasma

Sample preparation was performed at Metabolon Inc (Morrisville, NC). Briefly, individual samples were subjected to methanol extraction and then split into aliquots for analysis by ultrahigh performance liquid chromatography/mass spectrometry (UHPLC/MS). The global biochemical profiling analysis comprises four unique arms consisting of reverse phase chromatography positive ionization methods optimized for hydrophilic compounds (LC/MS Pos Polar) and hydrophobic compounds (LC/MS Pos Lipid), reverse phase chromatography with negative ionization conditions (LC/MS Neg), as well as a HILIC chromatography method coupled to negative (LC/MS Polar). All of the methods alternated between full-scan MS and data-dependent MS*n* scans. The scan range varied slightly between methods but generally covered 70–1000 *m*/*z*.

Metabolites were identified by automated comparison of the ion features in the experimental samples to a reference library of chemical standard entries that includes retention time, molecular weight (m/z), preferred adducts, and in-source fragments, as well as, associated MS spectra and curated by visual inspection for quality control using software developed at Metabolon. Identification of known chemical entities was based on comparison to metabolomic library entries of purified standards.
